# Salmonellosis: An Overview of Epidemiology, Pathogenesis, and Innovative Approaches to Mitigate the Antimicrobial Resistant Infections

**DOI:** 10.3390/antibiotics13010076

**Published:** 2024-01-13

**Authors:** Bibek Lamichhane, Asmaa M. M. Mawad, Mohamed Saleh, William G. Kelley, Patrick J. Harrington, Cayenne W. Lovestad, Jessica Amezcua, Mohamed M. Sarhan, Mohamed E. El Zowalaty, Hazem Ramadan, Melissa Morgan, Yosra A. Helmy

**Affiliations:** 1 Department of Veterinary Science, Martin-Gatton College of Agriculture, Food and Environment, University of Kentucky, Lexington, KY 40546, USA; 2 Botany and Microbiology Department, Faculty of Science, Assiut University, Assiut 71516, Egypt; 3 Faculty of Pharmacy, King Salman International University (KSIU), Ras Sudr 8744304, Egypt; 4 Veterinary Medicine and Food Security Research Group, Medical Laboratory Sciences Program, Faculty of Health Sciences, Abu Dhabi Women’s Campus, Higher Colleges of Technology, Abu Dhabi 41012, United Arab Emirates; 5 Hygiene and Zoonoses Department, Faculty of Veterinary Medicine, Mansoura University, Mansoura 35516, Egypt; 6 Department of Animal and Food Sciences, Martin-Gatton College of Agriculture, Food and Environment, University of Kentucky, Lexington, KY 40546, USA

**Keywords:** *Salmonella*, Foodborne pathogens, antibiotics, antibiotic resistance, antibiotic-alternatives

## Abstract

*Salmonella* is a major foodborne pathogen and a leading cause of gastroenteritis in humans and animals. *Salmonella* is highly pathogenic and encompasses more than 2600 characterized serovars. The transmission of *Salmonella* to humans occurs through the farm-to-fork continuum and is commonly linked to the consumption of animal-derived food products. Among these sources, poultry and poultry products are primary contributors, followed by beef, pork, fish, and non-animal-derived food such as fruits and vegetables. While antibiotics constitute the primary treatment for salmonellosis, the emergence of antibiotic resistance and the rise of multidrug-resistant (MDR) *Salmonella* strains have highlighted the urgency of developing antibiotic alternatives. Effective infection management necessitates a comprehensive understanding of the pathogen’s epidemiology and transmission dynamics. Therefore, this comprehensive review focuses on the epidemiology, sources of infection, risk factors, transmission dynamics, and the host range of *Salmonella* serotypes. This review also investigates the disease characteristics observed in both humans and animals, antibiotic resistance, pathogenesis, and potential strategies for treatment and control of salmonellosis, emphasizing the most recent antibiotic-alternative approaches for infection control.

## 1. Introduction

*Salmonella* is a foodborne pathogen that belongs to the family Enterobacteriaceae. It causes human gastroenteritis and can inhabit animals, amphibians, and reptiles [1,2]. The transmission of *Salmonella* to a healthy host occurs through the consumption of contaminated food and water [3,4]. *Salmonella* has been causing a significant impact on health and economics worldwide [5]. The World Health Organization (WHO) describes *Salmonella* as one of the four most important causes of diarrhea worldwide [6]. The Centers for Disease Control and Prevention (CDC) estimates that approximately 1.35 million people are infected with *Salmonella*, with about 420 deaths annually. The economic burden caused by *Salmonella* comes at the third position among a list of the annual cost of illness caused by 14 foodborne pathogens, with an annual cost of about $3.3 billion [7]. Annually, around 200 million to 1 billion cases of *Salmonella* infections are recorded worldwide, with 93 million cases of gastroenteritis and 155,000 deaths; among them, approximately 85% of the cases are associated with the consumption of contaminated food [8]. *Salmonella* outbreaks in 2022 alone in the US caused about 884 cases across 48 states between February and July, which were mainly attributed to poultry and poultry products [9,10]. *Salmonella* is classified as one of the category B pathogens with moderate morbidity and low death rates [11]. The severity of the infection in humans varies depending on the serotype of the bacteria and the immune status of the host, with the infection classified into typhoidal and non-typhoidal types [12]. Non-typhoidal *Salmonella* infections are often associated with acute onset of diarrhea, abdominal cramps, and fever [13]. It is usually self-limiting, resolving between 1 and 7 days without treatment, depending on the host status [14]. However, about 5% of people, including immune-compromised patients, infants, and older adults, may develop bacteremia or invasive infections such as meningitis, osteomyelitis, endovascular infections, and septic arthritis [10,15]. The typhoidal *Salmonella* serovars are responsible for non-specific disseminated infections, with symptoms including sustained fever (39–40 °C), headache, diarrhea or constipation, loss of appetite, and relative bradycardia [6,16,17,18,19,20].

*Salmonella* infects birds of all age groups. However, young chickens and turkeys are highly susceptible within the first two weeks of age. The disease is characterized by poor body condition, such as ruffled feathers, weakness, and anorexia. Additionally, infected birds tend to huddle together, exhibit diarrhea and a pasty vent, with decreased egg production, and post-mortem examination shows signs of a swollen liver and spleen with hemorrhages [21,22,23]. Studies have suggested that over 52% of *Salmonella* infections in poultry are caused by *S.* Enteritidis, making it one of the most prevalent serotypes of *Salmonella* in the US [24], whereas, according to the National Veterinary Services Laboratory, the most common serotype in livestock, especially cattle, was found to be *S.* Dublin (18%), followed by *S.* Cerro (16%) and *S.* Typhimurium (13%) [25].

Treating salmonellosis in humans and animals typically relies on antibiotics [26]. Broad-spectrum antibiotics are normally used to treat highly susceptible individuals with clinical complications [27]. Chloramphenicol and trimethoprim/sulfamethoxazole antibiotics were first used for the treatment of salmonellosis [28]. Currently, third-generation quinolones such as fluoroquinolones, including ciprofloxacin and ofloxacin, are the drug of choice for treating *Salmonella* infection in immunocompromised patients [29]. Due to the increasing bacterial resistance against fluoroquinolones, cephalosporins like ceftriaxone and macrolides like azithromycin are being used as empiric treatment to control *Salmonella* infections [15,30,31]. Like antibiotics, vaccines are also used to prevent and control *Salmonella* infections in humans and animals [32]. There are two vaccines for *Salmonella* approved by the Food and Drug Administration (FDA): the live attenuated Ty21a oral vaccine and intra-muscular Vi polysaccharide capsular vaccine, whereas several other vaccines such as the GMMA-based vaccine, glycoconjugate vaccine, O-antigen glycoconjugate vaccines, and new attenuated vaccines are still in development [33,34]. The effectiveness of vaccines against *Salmonella* is constrained by various factors such as the presence of asymptomatic carriers, which makes it difficult to design vaccines, complex immune evasion mechanisms, and the presence of diverse serotypes [35]. Currently, available typhoid vaccines provide only moderate and short-term protection in humans [36]. Additionally, *Salmonella* serotypes are highly variable, with significant genetic diversity within and between hosts, complicating the efforts to control the pathogen [37,38,39,40].

Therefore, there is a critical need for developing novel antibiotic alternative approaches to control *Salmonella* infections in animals and humans, including probiotics, prebiotics and bacteriophage, antimicrobial peptides, essential oils, and vaccines [40,41]. In this review, we discuss the epidemiology of salmonellosis with emphasis on transmission dynamics, host spectrum, clinical signs, the most recent outbreaks, and pathogenesis. We also provide insights on the current antibiotic treatment and emphasize the novel antibiotic alternatives developed/under development to control AMR-*Salmonella* infections in animals and humans.

## 2. Epidemiology of Salmonellosis

### 2.1. Salmonella Serotypes and Host Spectrum

Approximately 2659 *Salmonella* serovars were identified according to the White–Kauffmann–Le Minor scheme in the published supplement (no. 48–2014) [42]. *Salmonella* serovars are classified into typhoidal and nontyphoidal (NTS) according to their ability to develop specific pathogenicity in humans and animals [43]. Typhoidal serovars that cause typhoid and paratyphoid fever in humans include *S*. Typhi, *S.* Paratyphi A, B, C, and *S.* Sendai [44]. These serovars are highly host-specific and are only transmitted from infected hosts or carriers through contaminated food and water [45]. Typhoidal salmonellosis is characterized by high mortality and low morbidity [46]. However, NTS includes more than 2000 serotypes, which predominantly include *S.* Enteritidis, *S.* Typhimurium, *S.* Newport, and *S.* Heidelberg, and can infect both humans and animals [47]. Some NTS serovars like *S.* Typhimurium phage type DT2, *S*. Abortusovis, *S*. Typhisuis, *S*. Gallinarum, and *S*. Pullorum primarily infect pigeons, sheep, swine, aquatic birds, and poultry, respectively, whereas *S*. Dublin and *S*. Choleraesuis primarily infect cattle and pigs [48,49,50]. Moreover, NTS can easily adapt to a wide range of hosts and can quickly spread from infected hosts by consuming contaminated food and water [51]. The invasive nontyphoidal *Salmonella* [iNTS] are more virulent than other non-iNTS types; however, most of the iNTS serovars are similar to non-iNTS in terms of the type of illness, susceptibility to the high-risk group, and other characteristics such as the development of multidrug resistance [46]. The ability of *Salmonella* to adapt to the host’s environment and trigger clinical symptoms in that specific host is influenced by factors such as the dosage of the infecting bacteria, the host species involved, the age of the host, and its immune status [52]. For example, *S.* Choleraesuis serovar is a pig-adapted serovar, and it produces the most severe sickness in pigs compared to humans [53]. Some serotypes like *S*. *enterica* serovar Typhimurium have been listed as the prototypical broad host range serotype that can infect humans, livestock, domestic fowl, horses, swine, pigeons, rodents, and birds [51]. Other serovars such as *S*. *enterica* subspecies can be classified as host-generalist, host-adapted, or host-restricted [54]. They have developed mechanisms for surviving within the host while avoiding immune responses via colonizing the non-phagocytic cells [55]. For example, *S.* Typhi spreads from the gastrointestinal tract to the reticuloendothelial system. Moreover, it normally colonizes the surface of gallstones upon dissemination [56]. Approximately 1–6% of people infected with *Salmonella* Typhi do not display clinical symptoms after primary infections but become asymptomatic and chronic bacterial carriers [57,58]. Conversely, the pathogenesis of host-generalist serovars frequently results in gastroenteritis, and *Salmonella* shedding occurs for a very short time [59]. Because of their limited long-term shedding capability, the lifetime of host-generalist NTS is more dependent on their ability to survive in the environment [60].

### 2.2. Source of Infection and Mode of Infection Transmission in Humans and Animals

Because *Salmonella* species are thought to be part of the normal microbiota of an animal’s gut or gallbladder, these animals may also play a role in the pathogen’s indirect or direct transmission to humans [61]. The sources of *Salmonella* infection include (1) Poultry and poultry products, which are considered the primary source of *Salmonella* infection in humans [62]. Meat contamination occurs generally as a result of improper handling of the infected organs, such as the gut and liver, during carcass processing [63]. *Salmonella* infection in 44 broiler and 51 layer farms was investigated, where *Salmonella* was found in 41.3% of the broiler houses, and nearly 50% of the strains identified were capable of producing biofilm [64]. In the US, a previous report demonstrated that the prevalence of *S.* Enteritidis serovar in chicken products has grown from 0.45% to 1.5% within a period of 10 years (2002–2012), implying that poultry meat is one of the substantial risk factors for human infection [45]. Frozen raw breaded chicken products (FRBCP) have also been recognized as a *Salmonella* risk factor in Canada and the US [65]. From a list of 18 food sources, eggs and egg products were the most frequent sources of salmonellosis outbreaks [66]. (2) Ground meat: The CDC conducted a population survey which found that 82.2% of Americans consume beef weekly, with 67% explicitly preferring ground beef [10]. It was determined that chicken, pig, and beef were responsible for 34, 25, and 16% of *Salmonella* outbreaks, respectively [27,67], and 10% of human salmonellosis is attributed to beef consumption in the US [10]. A recent outbreak of salmonellosis has resulted in over 400 reported infections, with more than 100 individuals requiring hospitalization. The outbreak was attributed to antibiotic-resistant (AMR) *S*. Newport, which was traced back to the consumption of ground beef in 30 different states [68]. (3) Pets may contaminate the environment and transmit infection to other food-producing animals by sporadically shedding bacteria in their feces [69]. Pets like dogs fed on raw food diets are more likely to harbor *Salmonella* serovars such as *S.* Typhimurium, *S.* Heidelberg, and *S.* Kentucky. Moreover, the probability of *Salmonella* shedding was around 23 times higher in dogs on raw food diets than in dogs on commercial diets [70,71]. Furthermore, a case–control study on salmonellosis in children in Michigan revealed that exposure to cats is one of the major risk factors for *Salmonella* infection [72]. (4) Wild animals, including wild boar and feral pigs, play a crucial role in transmitting *Salmonella* to both domesticated animals and humans globally [73]. *Salmonella* is frequently detected in various wild mammals, such as opossums, raccoons, foxes, mink, tigers, cougars, seals, white-tailed deer, and whales, as well as wild birds [73]. Domesticated animals become infected through contact with the contaminated feces of wild animals and birds [74]. In humans, transmission commonly takes place either through direct contact with the contaminated feces from infected animals or from the consumption of contaminated meat from wild birds and other wild animals such as deer or wild boars [75]. Several studies have been conducted to determine the prevalence of *Salmonella* in wild animals. For example, Cummings et al., found that out of 442 fecal samples obtained from feral pigs across 50 counties in Texas, USA, 43% tested positive for *Salmonella*. Among these samples, the most prevalent serovars were *S*. Montevideo (10%), *S*. Newport (9.1%), and *S*. Give (8.2%) [76]. Likewise, Molino et al. demonstrated that upon analyzing tissue samples from 1041 wild boars from central–western Spain, 7.7% were positive for *Salmonella* and *S.* Newport was the most prevalent serovar [75]. Similarly, out of 225 fecal samples collected from captive wildlife and exotic animals including giraffes, cranes, and raccoons from Ohio, USA, 24.9% (*n* = 56) were positive for *Salmonella* and the most common serovars included *S*. Typhimurium (64.3%), *S*. Newport (32.1%), and *S*. Heidelberg (5.3%) [77]. (5) Insects are also one of the vectors for transmitting *Salmonella* in the farm setting. Research has demonstrated that houseflies and dump flies, namely *Musca domestica* and *Hydrotaea aenescens*, can carry *S.* Enteritidis, *S*. Heidelberg, and *S.* Infantis serotypes [78]. Similarly, larvae and adult lesser mealworms (*Alphitobius diaperinus*) have also been found to harbor AMR *S*. Enteritidis and transmit infections in farm settings [79]. Furthermore, 15 different serotypes, including *S*. Anatum, *S.* Choleraesuis var. kunzendorf, and *S*. Derby, were found in common house flies (*Musca domestica*) on a swine farm [80]. Moreover, 13 of these serotypes were found in swine fecal samples, with *S*. Anatum and *S*. Derby being the predominant ones [81]. (6) Rodents such as house mice are one of the significant sources of infection on farms. It was reported that the house mouse (*Mus musculus*) plays a crucial role in transmitting *Salmonella* Enteritidis infection among farm animals [82]. Additionally, species such as the roof rat (*Rattus rattus*) are also known sources of *S*. Enteritidis infections [83,84]. Various studies have reported that *R. rattus*, *R. norvegicus*, and *M. musculus domesticus* are all implicated as sources of several *Salmonella* serotypes in poultry and pig farms [83,85,86,87]. Similarly, the CDC defines other host species, such as reptiles and amphibians, as hosts that can harbor *Salmonella* and transmit the infection to humans and farm animals [9]. Additionally, the ability of *Salmonella* to form biofilms, enabling it to attach to and endure various environmental surfaces, vegetables, fruits, and chicken egg shells, as well as surfaces in proximity to animal living areas, like vacuum cleaner bags, sink drains, and doorknobs in households, helps in the further transmission of the bacteria to the mammalian hosts [46,88,89]. Other sources such as water, contaminated floors, carts, using contaminated water for crop irrigation, or direct contact with feces from animals carrying *Salmonella* can also transmit the infection to humans [90,91].

**Figure 1:**
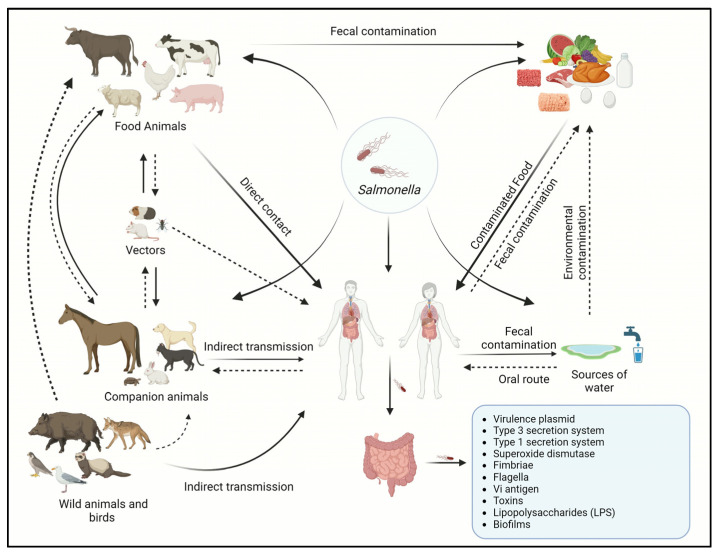
Transmission cycle of *Salmonella* between animals and humans.

The transmission of *Salmonella* serotypes often varies significantly between human and animal populations in the same geographical region [92]. Various *Salmonella* serotypes exhibit differing potentials for causing human disease [14]. However, the transmission of *Salmonella* infections can occur through direct or indirect contact at home, hospital, or farm settings; however, most of the *Salmonella*-related illnesses that occur globally each year are foodborne [93]. The transmission of *Salmonella* may occur by direct contact through direct consumption of fecal-contaminated food or water [94]. Vertical transmission occurs typically in birds and reptiles where the bacteria from the female reproductive tract obtain access to the eggs [95]. The introduction of the pathogen relies upon the thickness and permeability of an eggshell, where the reptiles’ eggshell is more thinner and permeable than avians [96], whereas indirect transmission occurs when the bacteria are transmitted through intermediate objects such as contaminated utensils and live or inanimate vectors [46]. The transmission cycle of *Salmonella* is shown in Figure 1.

### 2.3. Risk Factors and High-Risk Groups

Risk factors for a particular pathogen vary depending on the environmental stress the host and the pathogen endure [97]. According to the CDC, infections with *Salmonella* are more prevalent during the summer (June, July, and August) than in the winter [98]. Moreover, poorly breastfed infants, young children normally under the age of five years, elderly, and immunocompromised individuals are the most vulnerable to severe *Salmonella* infections [99,100]. Certain drugs, such as stomach antacids and antibiotics, can create gut dysbiosis, thus increasing the risk of *Salmonella* infections [101]. The development of clinical symptoms between animals can vary depending on various factors, including animal species, age groups, and geographical area. The risk factors for animal infections include stress, co-infection with another pathogen, and contaminated food [14]. The size of animal herds increases the risk of salmonellosis in farm animals, and bacterial shedding appears to be impacted by different factors such as production methods, housing types, general cleanliness standards, management practices, and the age of the animals [102,103,104,105]. Moreover, environmental factors such as dust, dirty surfaces, and chicken excrement are the known risk factors for acquiring the infections [106]. In humans, nail-biting, contact with animal excreta, sucking the thumb in children, and eating without properly sanitizing hands after farm work are considered potential risk factors for animal-acquired *Salmonella* infections [14,107]. Consuming contaminated food is one of the most significant risk factors in humans [108,109].

### 2.4. Clinical Signs in Humans and Animals

#### 2.4.1. In Humans

Typhoidal *Salmonella* serovars, such as *S.* Typhi or *S.* Paratyphi, are the causative agents of enteric fever, also known as typhoid or paratyphoid, respectively [110]. Globally, there are 11–21 million instances of typhoid fever and 5 million cases of paratyphoid fever each year, resulting in approximately 135,000–230,000 deaths annually. In the US, around 400 confirmed cases of typhoid fever and 5–100 cases of paratyphoid fever tested positive in cultures between 2016 and 2018. Notably, more than 85% of these cases occurred in individuals who had traveled internationally [94]. The incubation period of enteric fever is marked by a duration of one week or longer, during which individuals experience several symptoms, such as high fever, diarrhea, vomiting, and headache [92]. Throughout enteric fever, a notable fever pattern emerges. It begins with a low-grade fever (>37.5 °C to 38.2 °C) and gradually progresses to a high-grade fever (>38.2 °C to 41.5 °C) in the second week [111]. The fever can persist without appropriate treatment for a month or even longer [112]. In addition to fever, infected individuals may experience myalgia, bradycardia, hepatomegaly (enlarged liver), splenomegaly (enlarged spleen), and rose blotches on their chest and abdomen [113]. Approximately 15% of infected individuals in endemic areas experience gastrointestinal problems such as pancreatitis, hepatitis, and cholecystitis [114]. Hemorrhage is one of the most serious gastrointestinal complications caused by the perforation of Peyer’s patches, the lymphatic nodules found in the terminal ileum causing bloody diarrhea [115]. Furthermore, typhoidal *Salmonella*’s nature to live and remain in the reticuloendothelial system results in recurrence in around 10% of infected individuals [116].

Non-typhoidal *Salmonella* affects approximately 93.8 million people and causes 160,000 fatalities globally each year [6,117]. According to the current surveillance report in the US on NTS infections in humans, most of the isolated serovars are *S.* Enteritidis, *S*. Typhimurium, and *S*. Newport [118], while *S*. *enteritidis* are the most common serotype recovered from clinical samples in Asia, Europe, and Latin America [119]. The infection is typically self-limiting and the symptoms normally last for about a week [120]. The incubation period ranges from 6 h to 6 days after initial inoculation and the infection normally lasts for 4 to 7 days. Shedding of the bacteria via feces may last for a month or longer [121]. The most common human symptoms include gastroenteritis, accompanied by clinical signs including nausea, vomiting, headache, abdominal pain, non-bloody diarrhea, and muscle pain [122]. The severity of the infections increases in susceptible individuals such as babies and children under the age of five years, immunocompromised patients, and immunocompromised elderly people [123]. Conditions like cholecystitis, pancreatitis, and appendicitis may manifest and can escalate to severe levels, leading to life-threatening conditions like meningitis and sepsis [124]. Inadequate fluid balance due to prolonged loss of bodily fluids can lead to dehydration, which may be fatal in newborns and older adults [125]. Reactive arthritis, a persistent autoimmune joint inflammation, may supervene even after weeks or months of urogenital or digestive tract infections and occurs in around 20% of clinical cases reported in Europe and the US following *Salmonella* infections [126]. Furthermore, *Salmonella* infections are implicated in the development of colonic cancer in patients suffering from chronic inflammatory bowel disease (IBD) [127], the risk factor for colorectal and gallbladder cancer [128].

#### 2.4.2. In Animals

*Salmonella* infections are prevalent among various animals, encompassing both domesticated and wild species [129]. This bacterium typically affects the host gastrointestinal tracts, often without readily apparent symptoms of illness [130]. *Salmonella* can present itself at both clinical (symptomatic) and sub-clinical (asymptomatic) levels [131]. Poultry can serve as healthy carriers and the clinical signs in poultry depend on the bacteria’s serotype [132]. For instance, *S. enterica* serovar Pullorum causes anorexia, diarrhea, dehydration, and death in young poults, and adult birds demonstrate diarrhea, decreased egg production, poor hatchability, and increased mortality [133], whereas fowl typhoid can be characterized by acute diarrhea, dehydration, weakness, septicemia, and death [129]. Nevertheless, regardless of the bacterial serotype, all *Salmonella* infections in poultry are commonly characterized by pronounced symptoms, including extensive diarrhea, fever, weight loss, dehydration, and death [130]. Similarly, *Salmonella* infections in animals vary based on the age group and specific bacteria serotype, particularly in large and small ruminant animals [134]. Ruminants and pigs commonly exhibit acute enteric infections, characterized by clinical indications such as fever, reduced appetite, lethargy, and diarrhea. Conversely, systemic infections tend to be more prevalent among younger animals [135]. Notably, abortion has been extensively recorded in cattle specifically attributed to NTS serotypes *S.* Typhimurium and *S.* Dublin [136]. The infection in dogs and cats can be manifested by anorexia, fever, nausea, vomiting, acute gastroenteritis anorexia, abdominal pain, and diarrhea [137]. Similarly, horses are also considered a risk group for *Salmonella* infections, with atypical symptoms such as voluminous gastric reflux, diarrhea, and fever [138]. They can also serve as asymptomatic carriers of the bacteria, thereby shedding them into the environment and disseminating the infection throughout the farm or facility [139,140].

### 2.5. Prevalence of Salmonellosis and the Most Recent Salmonella Outbreaks

Currently, the advancement of science and technology and globalization have made international trade and travel easily accessible to the general population [43]. However, it has increased the risk of the rapid spread of infectious diseases throughout the world [119]. Controlling an outbreak of foodborne pathogens such as *Salmonella* can be challenging due to several factors, such as environmental factors and the high risk of indirect transmission through the consumption of *Salmonella*-contaminated food and water, which may originate from any source [130]. *Salmonella* infection presents significant public health concerns due to its propensity for endemicity, high rates of morbidity and mortality, and the challenge of implementing effective and timely control measures [119]. *Salmonella* causes approximately 1.35 million illnesses, with 26,500 annual hospitalizations and 420 fatalities in the US each year, as tracked by the Foodborne Diseases Active Surveillance Network (FoodNet) [140]. It was suggested that there is a substantial relationship between *Salmonella* serovar and the type and origin of the food commodity [141]. For example, outbreaks linked to poultry are generally associated with *S*. Enteritidis, *S*. Heidelberg, and *S*. Hadar, while outbreaks of *S*. Uganda have been associated with the consumption of contaminated pork and beef meat [141]. Outbreaks associated with farm products such as fruits and vegetables have also been documented [125]. Several reports suggest that improper handling of infected chicks is also responsible for a considerable number of human outbreaks of salmonellosis, mainly involving serovars such as *S.* Typhimurium, *S*. Johannesburg, *S.* Braenderup, *S.* Thompson, and *S.* Montevideo [43]. The serovars *S.* Typhimurium and *S*. Enteritidis are also linked with the zoonotic transmission of salmonellosis from companion animals such as kittens, guinea pigs, hedgehogs, and turtles [98].

*Salmonella* is a highly virulent pathogen and the presence of as low as 10 CFU/mL of bacteria typically represents a high potential for pathogenicity [142]. In 2018, about 92,000 confirmed human salmonellosis cases were documented in the US alone [143]. NTS causes over 100,000 gastroenteritis illnesses in Canada annually [144]. *S*. Enteritidis stands out as the predominant serovar, accounting for around 45% of human salmonellosis cases in Canada, followed by *S.* Typhimurium and *S.* Heidelberg, constituting 8% and 6% of such cases, respectively [145]. Similarly, *S*. Typhimurium is the most common serovar in humans in North America and Oceania, regardless of the source, followed by *S.* Enteritidis [146]. In contrast, *S*. Enteritidis ranked as the most common serovar in the European Union, followed by *S.* Typhimurium. However, *S.* Enteritidis was reported in pork only in Africa and Asia [147,148]. In Europe, a total of 1508 *Salmonella* outbreaks were included in the European Food Safety Authority (EFSA) analysis. Of these, 1040 were caused by foods, including salads, steak, and ham, whereas 468 outbreaks were caused by unknown food sources including complex foods like bakery products containing eggs, dairy products, and grains [148]. Approximately 939 outbreaks were recorded to be caused by *S*. Enteritidis, 130 by *S*. Typhimurium and its monophasic variant, 107 by other known serotypes, and 332 by unknown types in the European Union [66]. In May 2022, 324 cases were reported in 12 EU/EEA countries and the UK, including two distinct strains of monophasic *S*. Typhimurium. Most cases were in children below ten years of age, and 41% of all cases were hospitalized. Chocolate products in Belgium were reported to be a source of infection [149]. The most recent *Salmonella* outbreaks in the US, their source, and the identified serotype are shown in Table 1. Between 2012 and 2023, there were approximately 86 outbreaks, and 18,031 illnesses occurred in the US alone.

## 3. Pathogenesis and Virulence Factors

The pathogenesis of *Salmonella* serotypes starts with the adherence of the bacteria to the host cell surface [150]. After adhesion, bacteria’s internalization occurs either through the uptake of bacteria via phagocytosis or by active invasion of both phagocytic and non-phagocytic cells [27,151]. The phagocytosis process involves intricate mechanisms that rely on the engagement of multiple receptors, such as Pattern Recognition Receptors (PRRs) [152]. The PRRs include toll-like receptors (TLRs) and cytosolic nucleotide-binding receptors, which recognize pathogen-associated molecular patterns (PAMPs) like lipopolysaccharides (LPS) and flagellin located on either the cell surface or within phagosomes [27]. This recognition influences the maturation of phagosomes, triggers signaling pathways, and modulates gene expression [152,153,154]. Studies suggest that the interaction between the TLR and LPS in NTS species plays a vital role in developing septic shock [155]. However, the typhoidal serovars, including *S.* Typhi, evade recognition by TLR4, thus preventing the recruitment of neutrophils and the expression of pro-inflammatory molecules such as TNF-α and Interleukin 1β (IL-1β) and preventing a typical antimicrobial response in the host [154,156]. The level of production of the cytokines in human monocytes is, however, similar to those elicited in the NTS infections [157,158]. This is an essential stage in the invasion of *Salmonella* and occurs by infiltrating both phagocytic and non-phagocytic cells [159]. Invasion and colonization of *Salmonella* in the host cells rely on several virulence factors, including:

### 3.1. Virulence Plasmid

Virulence plasmids play a crucial role in bacteria by harboring genes related to antibiotic resistance and virulence factors such as *spvB* (ADP-ribosylating toxin) and *spvC* (inhibits pyroptosis and inflammation) [160,161]. Virulence plasmids are required to develop the systemic disease in the host and can spread through horizontal gene transfer by transformation and conjugation [162]. They are large and present in low copy numbers to minimize the strain on the host’s cell metabolism, preventing them from being retained during cell division [163]. In response, virulence plasmids have evolved to guarantee distribution, preserving their presence [163].

### 3.2. Type III Secretion Systems

Type III secretion systems (T3SSs) are responsible for translocating effector proteins from prokaryotic cytoplasm to the eukaryotic cytosol [164]. In *Salmonella*, the T3SS is encoded by two distinct pathogenicity islands, namely SPI1 and SPI2 [165]. SPI-1 encodes the T3SS1 and plays a crucial role in invading non-phagocytic epithelia [166]. SPI-2 encodes the T3SS2 effector proteins that function by regulating the dynamics of *Salmonella*-containing vacuole (SCV) membranes, placing SCVs in specific positions within host cells, influencing immune responses, modifying the cytoskeleton, and impacting the movement of infected cells [167,168]. These effector proteins combine to undermine the cytoskeleton, signal transduction pathways, and pro-inflammatory responses of the host [169].

### 3.3. Type 1 Secretion System (T1SS)

The Type 1 secretion system is responsible for delivering a wide range of molecules like lipases, surface proteins, toxins, and adenylate cyclase into the extracellular space of *Salmonella* [170]. It is also responsible for mediating adhesion and invasion into the host immune cells and biofilm formation [171]. Two distinct surface-associated proteins, *BapA*, responsible for adhering to host cells and forming biofilms, and *SiiE*, responsible for the initial attachment to host cells followed by invasion, are transported through a specialized Type 1 secretion system (T1SS) [172].

### 3.4. Superoxide Dismutase

Superoxide dismutase (SOD) is a group of enzymes that catalyze the conversion of superoxide radicals (O_2_^−^) into molecular oxygen (O_2_) and hydrogen peroxide (H_2_O_2_) [173]. Numerous host cells generate reactive oxygen species, primarily via the functioning of the phagosome NADPH oxidase, which is essential for eliminating intracellular pathogens [174]. To counterbalance this effect, *Salmonella* uses superoxide dismutases and SodCI and SodCII enzymes, which help the bacteria in cellular defense against reactive oxygen species [175]. Both of the enzymes are produced during the infections; however, SodCI relative to SodCII is tethered within the periplasm and is resistant to proteases [176]. This allows the enzyme to maintain functionality and help the bacteria survive in the phagosome’s challenging environmental conditions [177].

### 3.5. Fimbriae

Adherence to the host cells plays a pivotal role in the progression of *Salmonella* infection [178]. *Salmonella* possesses fimbrial gene clusters (FGCs) within its genome, which encodes extracellular fimbriae [178]. Among the extracellular fimbriae, one of the most prevalent adhesive structures is known as type 1 fimbriae (T1F) [179]. T1F is primarily composed of *fimA* protein and an adhesive protein *fimH*, which is critical in binding to specific receptors, preferably glycoproteins that carry terminal mannose residues [180]. The adhesive protein *fimH* is a pathogen-associated molecular pattern recognized by host TLRs and significantly influences the expression of pro-inflammatory cytokines [181]. 

### 3.6. Flagella

The motility of *Salmonella* is driven by the activity of flagella [182]. Flagella participates in adhesion, invasion, protein export, and biofilm formation [183]. Biofilm formation is regulated through the transcription factor *CsgD* [184]. *Salmonella* has two genes for flagellin, *fljB* and *fliC* [185]. Out of the two flagellin genes, the expression of *fliC* is more crucial in identifying specific sites on host cells than *fljB* [186]. In bacteria with impaired flagellar motility, there is an observable diminished adhesion and smaller colony formation in biofilms [176].

### 3.7. Vi Antigen

The *Salmonella enterica* serovar Typhi differs from NTS due to the production of the ‘Vi antigen’, a polysaccharide capsule located on the cell surface [187]. The Vi antigen inhibits phagocytosis and helps develop resistance against the host immune system [188]. It is also responsible for the translocation of *S.* Typhi to the gallbladder as it helps the bacteria to surpass the phagocyte-mediated barrier [189]. Ultimately, it prevents the binding of IgM, which gives the pathogen the ability to hinder neutrophil chemotaxis, neutrophil phagocytosis, and the neutrophil respiratory burst [190].

### 3.8. Toxins

One of the most significant features of *S.* Typhi is its ability to produce toxins resulting in typhoid fever [187]. This typhoid toxin belongs to the group of AB toxins, which include an enzymatic subunit (A) and a receptor subunit (B) [190]. *Salmonella*-containing vacuole exports toxin from infected cells into the external environment, allowing it to affect other target cells [190].

### 3.9. Lipopolysaccharides (LPS)

Lipopolysaccharides are a major component of the outer membrane of any Gram-negative bacteria responsible for eliciting innate immune response in the host [191]. It provides cell stability and acts as a permeability barrier [192]. LPS is made up of lipid A, core oligosaccharide (C-OS), and O-antigen polysaccharide (O-PS) [193]. LPS is also responsible for adherence or invasion of the host epithelial [192]. The proper distribution of O-antigen is required to express virulence in *S.* Typhimurium [194]. It is also responsible for determining antigenic specificity between and within the bacterial species [192].

### 3.10. Biofilms

Formation or the ability to develop biofilms is one of the major determinants of virulence in *Salmonella* inside the host [195]. Biofilms are the adaptive response that could alter the gene expression of the bacteria to promote resistance to both environmental stressors and antibiotics [195]. A *Salmonella* biofilm is formed by the secretion of a polymeric matrix characterized by the expression of different factors such as curli fimbriae and cellulose, which are the two predominant components [196]. Biofilm formation in *Salmonella* is regulated by *csgD*, a curli subunit gene belonging to the *LuxR* group [197]. The expression of *csgD* is regulated by various environmental signals and transcription factors such as c-di-GMP and sRNAs on a post-transcriptional level [198].

## 4. Control Strategies for *Salmonella* Infections

Various control strategies are employed to manage and prevent salmonellosis in humans. These measures encompass practices related to cleanliness and sanitation, consistent screening and diagnosis of individuals responsible for food handling, regular surveillance of potential carrier animals, and treating both carriers and those showing symptoms [20]. In animals, all stages of the production system should be regularly screened for *Salmonella* infection, including breeding facilities, vehicles, slaughterhouses, and storage facilities [199]. Several strategies can be used to prevent or control *Salmonella* infections in humans and animals, including:

### 4.1. Management and Biosecurity Measures

Control of salmonellosis in farm settings depends on good management and biosecurity practices [200]. To apply successful biosecurity programs and to control the spread of infection, the primary source of infection and the methods of transmission within the farm must be well identified [201]. Any successful biosecurity program must include isolation of sick animals, traffic limitation, disinfection, and sanitation of the farm [202]. Two types of biosecurity measures can be conducted to prevent or reduce the risk of infection flowing in and out of the farm, including external and internal biosecurity practices [203]. External biosecurity measures are pivotal in minimizing the influx of infections originating outside the farm premises. These strategies encompass the installation of perimeter fences, regulating the movement of vehicles to and from the farm and imposing restrictions on the introduction of animals from external sources [204], whereas internal biosecurity measures are designed to manage *Salmonella* transmission within the farm environment effectively. These tactics include changing footwear and clothing when transitioning from outside to inside the farm, isolating animals exhibiting symptoms from healthy ones, and routinely decontaminating the bedding material and transporting vehicles including dead animal transporters [205,206].

Farm visitors such as veterinarians, stakeholders, salespeople, and technicians are among the highest-risk visitors as a source of infection [207,208]. Furthermore, the need for more awareness among certain farmers regarding necessary safety precautions while moving in and out of the farm can potentially introduce *Salmonella* infection from neighboring farms and the environment. Failure to adequately clean or dispose of their clothing, boots, and tools can also result in contamination [208]. Several farm safety guidelines can be implemented to decrease the risk of infection disseminating from personnel, which includes (1) the movement of the visitors should be strictly restricted [209]; (2) visitors and workers must be supplied with clean outer clothes and boots [210]; (3) regular organic matter removal and provide footbaths with disinfectants, especially during working inside the farm [211]; (4) caring of the animals should always start with the healthy and the young stock and move to the sick and adult stocks [212]; (5) workers must not use the same tools for handling both food and manures or at least must be disinfected between use; (6) tools must not be borrowed from neighboring farms; (7) access to vehicles must be limited, especially in the farm premise, and vehicles must be cleaned and disinfected before entering the farm [208,213,214].

### 4.2. Vector Control and Eradication

According to the World Organization for Animal Health (WOAH), vectors are living organisms that are not only capable of transmitting a pathogen but also help disseminate the associated diseases in the population [215]. Some insects, rodents, and wild birds have been reported as sources of infection incidence, transmission, spread, and maintenance [216]. Rodents and wild birds can harbor the infections from different sources and transmit the infections to other farm animals through their feces on any part of the farm, including food and water; therefore, repeated disinfection is required with rodent control [217]. A high degree of sanitation must be applied, including litter and garbage disposal and proper filling up of any holes or openings to prevent access for mice. Moreover, supplies must be stored well in a clean area apart from the main building to avoid rodent access [218]. For the control of the carrier insects, a high level of sanitation must be maintained in animal farms and holdings, including regular and fast litter and waste removal, keeping the place well-ventilated and dry without any stagnant water [218,219]. Synthetic chemical insecticides and organophosphates can also be used regularly. These include permethrin, fenvalerate, tetrachlorvinphos (TCVP), dichlorvos organophosphate (DDVP), methomyl, benomyl, cyromazine, and dimethoate, but most of them have serious toxic effects on humans and animals, so specific instructions must be followed during their application [220]. Natural extracts such as essential oils with insecticidal or insect repellent activities and bioinsecticides formed of natural constituents can be used as a healthier and more eco-friendly, economical, and effective alternative [221]. Pyrethrin, a natural extract from chrysanthemum flowers, can be used with a lower level of toxicity [222]. Some essential oils like thyme, cinnamon, rosemary, clove, mint, orange, eucalyptus, and tea tree are considered to have established insecticide activity with lower toxicity and are registered to be among the commercially available constitutes of natural pesticides [218,219,223].

### 4.3. Isolation and Quarantine

The principle of quarantine mainly focuses on two primary goals: prevention of infection transmission to healthy animals and prevention of transmission in the hospital setting to vulnerable individuals such as immune-compromised patients, children, and the elderly [224]. Isolation of a sick person or animal and limiting contact with such individuals will significantly reduce the risk of contamination and the spread of the disease between humans and animals. The isolation units should be away from the healthy sheds and should have a proper manure disposal facility [225]. Regularly cleaning the farm equipment, utensils, feeders, and drinkers and relevant safe transportation and disposal procedures for contaminated carcasses are urgently required [226]. The duration of the quarantine period varies according to the type of pathogen and the status of exposure to the pathogen [112]. For individuals who are healthy and have been exposed, the quarantine period should align with the pathogen’s incubation period. Conversely, for infected animals, the quarantine duration should be determined by the time it takes for symptoms to manifest, along with confirmation through laboratory diagnosis [227]. Together, applying these control measures as “biosecurity and hygienic management” can positively impact food safety, and animal, and human health.

### 4.4. Antibiotics Used for Salmonella Treatment and Antimicrobial Resistance

The treatment of *Salmonella* infections typically relies on supportive therapy [228]. The infection is normally self-limiting, and the individuals do not require therapeutic treatment. However, individuals with weakened immune systems, underlying health conditions, or severe infection might require antibiotics [229]. In the past, chloramphenicol was utilized for treating *Salmonella* infections. The preferred antibiotic choices include ampicillin, third-generation quinolones such as ciprofloxacin and levofloxacin, third generation cephalosporins like ceftriaxone, and macrolides [230]. Unfortunately, bacterial resistance to these important antibiotics has been growing, posing a challenge to effective treatment [231]. Antibiotic resistance has become a global concern in both non-Typhoidal and Typhoidal *Salmonella* strains [232]. The emergence of antibiotic resistance has exhibited an escalating trend of 20–30% per decade [233]. The extent of resistance, however, varies across different antibiotics and serotypes of the bacteria, highlighting the delicate interplay between microbial genetic factors, environmental conditions, and the selective pressures that contribute to the diverse spectrum of AMR strains observed within bacterial populations [234]. It is noteworthy that serotypes with higher prevalence tend to develop resistance against commonly prescribed antibiotics more frequently [232]. It was reported that 30.9% of isolated *Salmonella* strains from broiler farms exhibited resistance to streptomycin, with 13.9% resistant to tetracycline, 12.6% resistant to gentamycin, and 8.6% resistant to sulfamethoxazole-trimethoprim [26]. Similarly, a substantial level of resistance was noted towards ceftriaxone (75%) and ceftiofur (44%) [235]. Multidrug-resistant *Salmonella* was also identified in several studies before. For instance, MDR was detected in 17% of broiler chickens in Egypt, with the highest resistance against neomycin (100%), nalidixic acid and cefoxitin (95%), norfloxacin (86.3%), cefotaxime (77.2%), amikacin (72.7%), erythromycin (68.1%), and chloramphenicol (40.9%) [236]. Similarly, 19.6% of *S*. Infantis isolates from animals in the US possessed MDR, with the highest resistance observed against aminoglycosides, chloramphinecol, beta-lactams, and tetracyclines [237]. Furthermore, in *Salmonella* isolated from equines between 2007 and 2015, 10.2% of the samples were MDR strains, with the highest resistance against aminoglycosides (gentamycin and streptomycin), followed by beta lactam inhibitors including penicillin (amoxicillin-clavulanic acid and ampicillin), cephems (cefoxitin, ceftiofur, and ceftriaxone), and folate pathway inhibitors (sulfisoxazole and trimethoprim), respectively [238]. Furthermore, MDR has also been demonstrated in wild animals and birds. For example, Cilia et al. found that AMR strain prevalence in European wild boar hunted in Central Italy possessed 55.6% resistance to streptomycin, 11.1% to cephalothin, and 5.6% to imipenem. Notably, a single isolate (*S*. Infantis) displayed multidrug resistance (MDR) to tetracycline, enrofloxacin, nitrofurantoin, nalidixic acid, and streptomycin [73].

Additionally, another investigation established a significant link between the isolation of ceftiofur-resistant *S*. Heidelberg from chickens and subsequent clinical infections in humans caused by the same bacterial strain [239]. Likewise, another study underscored the elevated prevalence of AMR strains, including *S.* Bredeney, *S.* Kentucky, and *S.* Enteritidis, as prominent AMR variants identified in chicken meat. These strains displayed resistance against rifampicin, tetracycline, and oxyclozanide [240].

### 4.5. Novel Antibiotic Alternatives

#### 4.5.1. Probiotics

**Figure 2:**
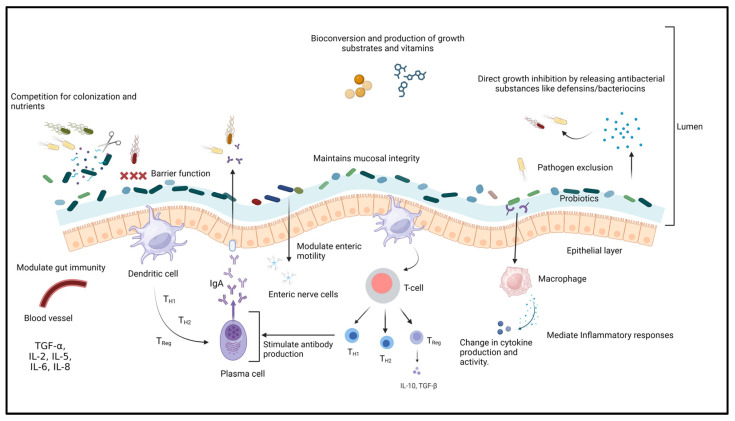
Mechanism of action of probiotics.

Probiotics are a group of non-pathogenic microorganisms that can confer health benefits to the host when administered sufficiently [241,242]. According to FAO/WHO regulations, for probiotics to be used as therapeutic or prophylactic agents, they are required to fulfill specific criteria such as safety margin, efficacy, immunomodulatory capabilities, ability to effectively colonize the intestinal epithelium, resistance to bile salts and low pH conditions, as well as maintaining phenotypic and genetic stability [243,244]. Probiotics have different mechanisms of action (Figure 2) that include (i) improving the intestinal barrier and gut mucosal integrity, (ii) enhancing intestinal immunity, (iii) reducing the colonization of intestinal pathogens, (iv) maintaining the balance between pathogenic and beneficial microbes in the gastrointestinal tract, and (v) competitive exclusion and secretion of antibacterial substances or metabolites such as bacteriocins that suppress the growth of pathogenic microorganisms, stimulating mucous secretion by intestinal goblet cells to limit epithelial invasion by pathogens and the production of minerals, enzymes, and trace elements [194,245,246,247,248,249]. Each probiotic strain has different properties and clinical effects on the host [250]. Probiotics are classified as mono-strain or single-strain probiotics (SSP), multi-strain probiotics (MSP), and multi-species probiotics [251,252]. Single-strain probiotics (SSP) can provide limited health benefits to the host [253]. Probiotics containing multiple groups of bacteria with different mechanisms of action tend to have synergistic effects on each other and have a broad spectrum of activity [252,254]. For insistence, the MSP of *Bacillus amyloliquefacrem*, *Enterococcus hirae*, and *Lysinibacillus fusiformis* was able to significantly inhibit the growth and biofilm formation of *Aeromonas hydrophila* compared to the individual probiotics [255]. Multi-species probiotics (*L. reuteri*, *E. faecium*, *B. animalis*, *P. acidilactici*, and *L. salivarius*) greatly reduce *S.* Enteritidis infections (up to 2.7 log reduction) in poultry [256].

Several studies have shown that probiotics can profoundly affect the growth and virulence of *Salmonella* in humans and animals [257]. These effects include preventing adhesion and invasion of the bacteria into the intestinal epithelial cells, alteration in the expression of virulence genes, modulation of the host immune system through enhancing the cytokines’ expression, intestinal permeability, and increasing intestinal villi height [258,259]. It was reported that *Lactobacillus* and *Bifidobacteria* are the most common probiotics used against *Salmonella* and are present as normal gut microflora in the host [260,261]. Many studies have demonstrated that using single strains of probiotics individually and in combination can show high efficacy in *Salmonella*-infected hosts (Table 2). For example, *L. salivarius* CTC2197 alone completely inhibited *S.* Enteritidis C-114 from the gut of a leghorn chicken 21 days post-infection [262]. Similarly, *L. reuteri* R-17485 alone demonstrated more than 1 log reduction, whereas *L. johnsonii* R-17504 demonstrated a 2 log reduction in cecal *Salmonella* count in Lohmann White laying hens [263,264]. Another study demonstrated that probiotic *L. plantarum* caused a 2.1 log reduction in cecal *S.* Heidelberg in broiler chicken 168 h post-infection [265]. In addition to this, other probiotics such as *E. faecium* NCIMB 11181 demonstrated a reduction in colonization and translocation of *Salmonella* in liver tissue by 2.2 log and cecal content by 4.2 log in infected birds pretreated with *E. faecium* [266]. Similarly, *S.* boulardii demonstrated enhanced survival of the probiotic-treated mice (70%) compared to 40% in untreated ones, with reduced translocation of *Salmonella* to the liver [267].

On the other hand, the combination of different *Lactobacillus* strains, including *L. murinus*, *L. salivarius*, *L. pentosus*, and *P. pentosaceous,* demonstrated up to 99% inhibition in *Salmonella* colonization in pigs, whereas the combination of other strains, such as *L. reuteri* R-17485, *L. johnsonii* R-17504, and *L. vaginalis* R-17362, demonstrated up to two-fold reduction in *Salmonella* cecal counts in chickens. Furthermore, the combination of the probiotic *Lactobacillus* with other species such as *Enterococcus faecium*, *Bacillus subtilis*, *Bifidobacterium animalis*, *Clostridium butyricum*, or *Saccharomyces cervisae* has a synergistic action with a significant inhibition (up to 95%) of *Salmonella* colonization in poultry and mice [256,268,269]. Several different experiments demonstrate that the combination of either two or more probiotics for treating *Salmonella* can have synergistic effects and may become more effective in inhibiting growth and colonization in the host (Table 2) [270].

However, the use of probiotics for the treatment of infectious diseases, including *Salmonella*, presents itself as a multifaceted approach, and further studies need to be conducted to determine whether their efficacy is contingent upon strain-specific factors of the pathogen or influenced by variables such as probiotic dosage, administration method, treatment duration, host characteristics (including age), and other management-related factors [258]. Moreover, there is a pressing concern regarding the clinical applications of probiotics, which includes issues such as the shelf life that may impact the viability of probiotic strains, their ability to withstand the conditions of the gastrointestinal tract, the potential for acquiring virulence or resistance genes from pathogenic or opportunistic organisms, the capacity of specific probiotic strains to transfer antibacterial resistance genes within the gastrointestinal tract, and the possibility of some probiotic strains like *B. subtilis* secreting toxic substances which can potentially induce food poisoning [271].

**Table 2:** Probiotics and their therapeutic uses against *Salmonella* serotypes in different hosts.

Probiotics	Dose	Animal Host	*Salmonella* Serotype	Dose	Results	References
*L. alvi* An810, *L. ingluviei* An777, *L. reuteri* An769, and *L. salivarius* An63	10^7^ cfu/mL	Chicken (male ISA Brown)	*S.* Enteritidis	10^5^ cfu/mL	No protective effect against *S.* Enteritidis in the host.	[272]
*L. acidophilus* LAP5, *L. fermentum* P2, *Pediococcus acidilactici* LS, and *L. casei* L21	10^7^ CFU/mL	Broiler chicken	*S*. *enterica* subsp. Enterica ST19	10^8^ Cfu/mL	Modulation of intestinal microbiota, increases intestinal villi height and short-chain fatty acids, restoring intestinal permeability by preventing tight junction damage.	[258]
*L. reuteri*, *E. faecium*, *B. animalis*, and *P. acidilactici*	0.5 g/kg feed	Cobb broiler chickens	*S*. Enteritidis	10^9^ Cfu/mL	The growth and proliferation of *S.* Enteritidis decreased to 87.4–99.5% in vitro, and *Salmonella* load decreased by 0.85 and 1.5 log units/mL for cecal and carcass contents, respectively.	[268]
*B. subtilis*, *B. licheniformis* and Mannan oligosaccharide	1.5 lbs/ton of feed	Hy-line layer hens	*S.* Enteritidis	3 × 10^6^ cfu/bird	A significant decrease (1.94 log reduction) in *Salmonella* colonization in the ceca.	[269]
*E. faecium* NCIMB 11181	4 × 10^8^ cfu/kg of diet	Broiler chickens (Arbor	*S.* Typhimurium CVCC 2232	10^9^ cfu/mL	Significant reduction in colonization and translocation of *Salmonella* in liver tissue (2.172 logs) and cecal content (4.2 logs) of infected birds pretreated with *E. faecium.*	[266]
*L. salivarius* CTC2197	10^5^ cfu/mL	Leghorn chickens	*S*. Enteritidis C-114	10^8^ cfu/mL	Complete clearance of *Salmonella* in chicken’s gut 21 days post-infection.	[262]
*L. fermentum* IKP 23, *L. fermentum* IKP 111 and *L. salivarius* IKP 333)	10^7^ cfu/mL	Broiler chickens	*S.* Enteritidis	10^6^ cfu/mL	Intestinal villus height was improved. Significantly high concentration of D-xylose in the plasma of broilers.	[273]
*L. plantarum*	1.8 × 10^8^ cfu/mL	Cobb broilers	*S.* Heidelberg	2.5 × 10^8^ cfu/mL	*S.* Heidelberg count was decreased in the caeca (2.1 log reduction).	[265]
*L. salivarius* L38 and *L. acidophilus* L36	10^9^ cfu/mL	Swiss NIH mice	*S*. Typhimurium	10^7^ cfu/mL	No indication of protection against *Salmonella* isolates after pre-treatment with L36 or L38 probiotic strains.	[274]
*L. reuteri* R-17485, *L. johnsonii* R-17504 and *L. vaginalis* R-17362	2 × 10^8^ cfu/mL	Lohmann White laying hens	*S*. Enteritidis	10^4^ cfu/mL	One-fold reduction in the cecal *Salmonella* count by *L. reuteri* R-17485, whereas significant (2-log) reduction by *L. johnsonii* R-17504.	[263,264]
*L. reuteri*, *E. faecium*, *B. animalis*, *P. acidilactici* and *L. salivarius*	2 × 10^9^ cfu/kg diet	Cobb broilers	*S.* Enteritidis	6 × 10^5^ cfu/mL	Administration of probiotics to birds resulted in 2.7 log reduction in *Salmonella* in the cecum.	[256]
*L. acidophilus*, *B. bifidum*, and *Streptococcus faecalis*	1 × 10^5^ to 1 × 10^6^ cfu/mL	Female crossbred broiler	*S.* Typhimurium	10^4^ cfu/mL	Low- and high-dose treatment with probiotics resulted in 1.2 and 3 log reductions in *S.* Typhimurium load in chickens’ cecum, respectively, and decreased IFN-γ gene expression in the cecal tonsils of the treated chickens.	[275]
*L. murinus*, *L. salivarius*, *L. pentosus*, and *P. pentosaceous*	4 × 10^9^ cfu/mL	Pigs	*S.* Typhimurium	10^8^ cfu/mL	2.4 log reduction (from 3.68 to 1.4 log CFU) in the fecal count of *Salmonella*.	[276]
*L. fermentum* and *L. acidophilus*	10^8^ cfu/mL	Mice	*S.* Typhimurium	10^5^ cfu/mL	No significant difference between treated and nontreated mice.	[277]
*L. plantarum* Z01	10^8^ cfu/mL	Broiler chicken	*S.* Typhimurium	10^8^ cfu/0.2 mL	Significant reduction in *Salmonella* from the cecal content of treated chicken (5.24 out of 252 cfu × 10^5^/g).	[278]
*B. subtilis*	10^8^ cfu/mL	Intestinal epithelium	*S.* Enteritidis, *S.* Typhimurium	10^8^ cfu/mL	High inhibition of *S.* Enteritidis (11–12 mm) and *S.* Typhimurium (11–15 mm zone of inhibition).	[257]
*E. faecalis*, *C. butyricum*, and *B. mesentericus*	3.48 × 10^8^, 2.0 × 10^7^, 1.1 × 10^7^ cfu/mL	Hospitalized infants and children	*Salmonella* spp.	-	Significant reduction (*p* < 0.0001) in diarrheal symptoms and severity of diarrhea significantly improved (*p* < 0.01) 3 days and no diarrhea was observed 5–7 days post-treatment.	[279]
*B. subtilis* RX7 and *B. methylotrophicus* C14	10^9^ cfu/g	Weaned pigs	*S.* Typhimurium	10^11^ cfu/mL	*Salmonella* counts in piglets after *B*. *subtilis* and *B. methylotrophicus* treatment have been reduced to 3.57–3.69 log cfu/g compared to the control group.	[280]
*L. plantarum*, *L. casei*, *L. acidophilus*, and *E. faecium*	10^7^ cfu/g	Horses	*S.* Typhimurium	-	Up to 65% reduction in fecal *Salmonella* shedding.	[281]
*S. boulardii*	10^9^ cfu/mL	Mice	*S.* Typhimurium	10^5^ cfu/mL	Enhanced survival up to 70% in treated mice as compared to 40% in untreated ones. Decreased *Salmonella* translocation, reduced liver damage, and decreased inflammatory cytokines	[267]
*E. coli Nissle* 1917 (EcN)	10^9^ cfu/mL	Day-old laying chicken	*S.* pullorum	10^7^ cfu/mL	Reduction of 2 log in the invasion of *Salmonella* in chicken fibroblast cells and 60% survival rate in EcN-treated group compared to 40% in the untreated ones.	[282]
*L. lactis* IBB 500, *L. casei* ŁOCK 0915, *L. plantarum* ŁOCK 0862 and *S. cerevisiae*	10^9^ cfu/mL	Ross-308 broiler chickens	*S.* Enteritidis	10^5^ cfu/mL	Reduction of 2-fold in cecal *Salmonella* 14 days post-infection followed by 0.5-fold reduction (*p* < 0.05) at 42 days post-infection.	[283,284]

#### 4.5.2. Prebiotics

Prebiotics are defined as the non-digestible components that undergo selective fermentation, resulting in targeted modifications to the composition and behavior of the gastrointestinal microbiota. When the microbiota utilizes these components, they contribute to beneficial effects on the health of the host [285]. Prebiotics are usually combined with probiotics in commercial products and are known as: “Synbiotics, beneficial microorganisms with selective substrates”. This combination has great therapeutic efficacy against various animal and human diseases [286,287,288]. Several prebiotic compounds are available, including fructooligosaccharides (FOS), galactooligosaccharides (GOS), mannan-oligosaccharides (MOS), xylooligosaccharides (XOS), transgalactic-oligosaccharides (TGOS), arabinoxylo-oligosaccharides, lactulose, and inulin [289,290]. The human digestive enzymes do not normally digest these compounds but they could be introduced into the diet in certain quantities to stimulate the gut microbiota, which, in turn, can provide the host with the essential nutrients and energy [291]. The mechanism of action of prebiotics can be summarized into direct and indirect pathways [292,293,294,295,296]. The indirect pathway is through nourishing beneficial gut flora and maintaining gut health, thereby conferring health benefits to the host, whereas the direct pathway acts through the inhibition of pathogenic microorganisms and reduces the risk of infection with infectious pathogens [297]. Several studies have been conducted to determine the protective effects of probiotics and prebiotics in experimental animals, including poultry infected with *Salmonella* [298]. For example, it was reported that the administration of *B. subtilis, Bacillus licheniformis*, and mannan-oligosaccharide revealed a significant (up to 2 logs) reduction in *S.* Enteritidis colonization in layers of ovaries and the intestine [269]. Similarly, chickens administered with inulin and oligofructose had up to a four-log reduction in cecal *Salmonella* counts possibly due to the effect of administered prebiotics on the pH and the level of produced volatile fatty acids [299]. Similar results were reported on the supplementation of broiler chickens with 0.75% oligofructose, where a four-fold reduction in cecal *Salmonella* counts was demonstrated [300]. Furthermore, the dietary supplementation of broiler chickens with fructooligosaccharides demonstrated a log reduction in intestinal colonization and count of *S.* Typhimurium [301]. Similarly, feeding broiler chickens with a combination of prebiotics (fructooligosaccharides) and probiotics (*B. animalis*, *L. reuteri*, *P. acidilactici*, and *E. faecium*), as well as prebiotics with antibiotics, resulted in decreased *S.* Enteritidis load by 1.4 and 1.5 log units/mL of carcass rinse and 0.90 and 0.85 log units/g of cecal contents, respectively [268]. In another study, the treatment of *S.* Enteritidis challenged turkey poults with *Lactobacillus* spp. And dietary lactose (0.1%) revealed significant improvement in body weight and feed conversion ratio with a 2 log reduction in cecal *S.* Enteritidis count [302]. Furthermore, a study for the evaluation of the effectiveness of synbiotics alone or in combination with organic acids on carcass and cecal *Salmonella* load in challenged one-day-old broiler chicks revealed a 0.34 to 0.58 log reduction in the *Salmonella* cecal contents compared to the controls, whereas no difference was observed between the dietary treatments [303]. Similarly, the treatment combination of synbiotics with organic acids revealed a 1.7 log reduction in carcass bacterial count, whereas a 1.3 and 0.53 log reduction was observed in *Salmonella* loaded with synbiotics alone and synbiotics combined with organic acids, respectively [303]. Nevertheless, some studies have demonstrated no effect of prebiotics in protective efficacy against and susceptibility to pathogenic infections. For example, *S.* Typhimurium translocation in the liver, mesenteric lymph nodes, spleen, and intestine have been increased, with an approximate 1.6–1.8 mean CFU in mice fed on a diet containing 10% of fructooligosaccharide, xylooligosaccharides, or apple pectin [304]. Similarly, no effect was observed in the production of anti-*Salmonella* antibodies in birds challenged with *S.* Enteritidis or broiler chickens fed with a combination of probiotics (Lac XCL 5x™) and prebiotics (MOS) [305]. Furthermore, another study demonstrated that no anti-*Salmonella* effect was seen in birds tested for the symbiotic effect of *B. longum*, and *L. rhamnosus* combined with oligofructose-enriched inulin on *S.* Typhimurium-challenged pigs [306]. In addition to this, evaluation of the efficacy of probiotics alone (*L. acidophilus*, *B. subtilis*, *L. casei*, *B. longum*, and *E. faecium*), prebiotics alone (fructooligosaccharide, inulin, oligosaccharide, and mannanoligosaccharide), and synbiotics (combined pro- and prebiotics) on *S.* Enteritidis challenged one-day-old layer and broiler chicks and concluded that the group of chicks supplemented with prebiotics only demonstrated a higher reduction in SE colonization (3 log reduction) when compared to groups supplemented with probiotics alone or synbiotics alone [307]. Hence, prebiotics play a vital role in maintaining gut health, linked to a range of health advantages like better digestion, synergistic actions along with the gut microbiota and supplemented probiotics, exclusion of pathogens, and improved growth performance [303].

#### 4.5.3. Antimicrobial Peptides

Antimicrobial peptides (AMPs) are a diverse group of small peptides that are an essential part of the innate immune system of different organisms [308]. The updated AMPs database reports more than 3569 AMPs identified, most of which originated from bacteria, followed by animals, plants, fungi, protists, and archaea [292]. There are several types of AMPs with various numbers of amino acid residues ranging from 10 to 60 amino acids, most of which are cationic, and some are non-cationic AMPs [309,310]. AMPs have two mechanisms of action: membrane-targeting and non-membrane-targeting mechanisms [309]. The membrane-targeting mechanism can be classified into three models: (1) carpet-like model in which AMPs are arranged parallel to the cell membrane like a carpet and destroy the pathogen’s membrane [311], (2) the barrel-stave model in which AMPs aggregate with each other and penetrate the membrane bilayer, forming channels that cause cytoplasmic leakage, thus resulting in cell death [312] and, (3) the toroidal pore model through which AMPs are vertically embedded in the cell membrane and bend to form a ring hole [310].

The non-membrane-targeting mechanism can be classified according to the targets by which AMPs act after entering the cytoplasm, which includes (i) protein biosynthesis inhibition [313,314], (ii) nucleic acid biosynthesis inhibition [315], (iii) inhibition of metabolic activities [316], and (iv) inhibition of DNA replication and cell division [317]. In addition to the broad-spectrum antimicrobial properties of AMPs, they are potential antibiotic substitutes with a low probability of developing AMR strains [318].

Several studies have evaluated the antimicrobial efficacy of AMPs against foodborne pathogens including *Salmonella* [318]. These studies evaluate their efficacy on immune regulation, growth performance, and intestinal microbiota in different animal species. For example, Festa et al. demonstrated the in vitro effect of peptide 1018-K6 against *S*. *enterica* (1 × 10^3^ cfu/mL) with a Minimum inhibitory Concentration (MIC) and Minimum Bactericidal Concentration (MBC) of 8–64 μg/mL and 16–128 μg/mL, respectively, and the sub inhibitory dose significantly reduced the biofilm formation of *S.* Enteritidis [319]. In vitro, evaluation of the effect of novel AMP “A11” modified from acidocin J1132β against *S.* Typhimurium demonstrated a complete inhibitory effect of A11 against *S.* Typhimurium, with an MIC ranging from 15.6 to 125 µg/mL [320]. Similarly, the screening of six different AMPs (KLK and KLK1) derived from flesh fly larva, BmKn-2, and BmKn-22 derived from scorpion venom, and Pug-1 and Pug-4 derived from pomegranate fruit), against eight multidrug-resistant *Salmonella* isolates showed that BmKn-2 derived from scorpion venom has the highest and most potent antibacterial activity against all isolates and the highest inhibition of *Salmonella* biofilm formation compared with other peptides [321]. Another in vitro study determining the antimicrobial activity of the modified thermostable cathelicidin-derived peptide, P7, against drug-resistant *S.* Typhimurium showed that P7 decreased the *S.* Typhimurium viable cells to more than 10^3^ and 10^4^ cfu/mL within 2 to 4 h, respectively, and also demonstrated complete clearance of *Salmonella* 24 h post incubation [322]. Furthermore, it was reported that AMP (Microcin J25) could significantly reduce the infection rate of *Salmonella* CVCC519 by approximately 30% in challenged Arbor Acres male broiler chickens at day 42 compared to non-treated birds [183]. Moreover, the AMP (Microcin J25) secreted by ECN has been recorded to lower the in vitro growth of *S.* Enteritidis on agar plates and also resulted in a 25× reduction in *S.* Enteritidis count and colonization in turkey cecum [323]. Similarly, an investigation determining the efficacy of AMP (HJH-3) in challenged chickens with *S.* Pullorum demonstrated a more than 48-fold reduction in total bacteria in the spleen of the HJH-3 group compared to the non-treated group [324]. Yeom et al. also reported that AMP (C-terminally hexahistidine-tagged A3-APO) loaded onto gold nanoparticle DNA conjugate completely inhibited and eliminated intracellular *S.* Typhimurium in challenged mice, resulting in 100% survival of infected mice [325]. Two AMPs (IK12 and TS10) were compared for their efficacy against *S. enterica* in fish, and the results demonstrated that AMP (IK12) exhibited a significant inhibition zone (20 ± 1 mm) against *S. enterica* at a concentration of 625 μg/mL, while concentrations of 1000 μg/mL and 2500 μg/mL decreased the *Salmonella* load up to 6 log [326]. In addition to this, the treatment of *S.* Typhimurium-challenged mice with AMP (Css54) demonstrated that Css54 can inhibit *S.* Typhimurium growth at a concentration of 6.25 μg/mL, while complete bactericidal activity can be obtained at a concentration of 25 µg/mL [327]. Similarly, supplementing *S.* Enteritidis-challenged female laying chicks with two different doses of AMP (Ctx(Ile^21^)-Ha) at the rate of 20–40 mg/kg feed for 28 days revealed a reduced mortality rate in young chickens by 69%; however, no difference in the mortality rate was observed even after increasing the Ctx(Ile^21^)-Ha dose concentration [328]. Maiti et al. demonstrated that avian defensin 7 (AvBD7) significantly reduced *S.* Typhimurium load in the liver (80% reduction) of treated mice 24 h post-infection in intraperitoneally challenged mice [329]. In addition to this, evaluating the efficacy of two human β-defensins (hBD-1 and hBD-2) in mice challenged intraperitoneally with *S.* Typhi demonstrated the 50% lethal doses of hBD-1 and hBD-2 to be 0.36 and 0.38 μg/μL respectively, with a significant reduction in *Salmonella* load in the peritoneal fluid, spleen, and liver of treated mice whether hBD-1 and hBD-2 is delivered individually or in combination [330]. A study to evaluate the antimicrobial activity of three gallinacin AMPs (GALL 4, 7, 9) against *S.* enteritidis revealed that the antimicrobial potency was in the following peptide order (Gall 9 > 4 > 7), with a synergistic action observed between Gall 7 and 9, whereas an in vivo study in non-domesticated fowl demonstrated no significant effect in the expression of gal4 or gall9 [331].

The capacity of AMPs to effectively target a wide range of bacterial pathogens, including *Salmonella*, holds significant promise in addressing the persistent problem of AMR strains. Nonetheless, despite their immense potential, several obstacles, such as the bacteria’s ability to develop resistance to these compounds and potential toxicity to host cells, pose significant challenges in developing them as alternatives to antibiotics. Thus, it becomes imperative to embark on further research to gain a more profound understanding of the precise mechanisms by which AMPs operate, improving their bioavailability and allowing us to devise cost-effective methods for their production. This holistic approach is essential to harnessing the full potential of AMPs as alternatives to traditional antibiotics and addressing the pressing global concerns surrounding AMR strains.

#### 4.5.4. Bacteriophages

Bacteriophages, also known as phages, are viruses with the unique capability to infect bacteria [332]. A bacteriophage consists of a protein capsid housing containing DNA or RNA as its nucleic acid core [333]. They can undergo replication within the bacterial cell through two distinct cycles: the lytic cycle and the lysogenic cycle [332]. (1) The lytic cycle is when a bacteriophage takes control of a bacterial cell and replicates itself, causing the lysis of the bacteria [334]. This process involves the bacteriophage reprogramming the host cell, transforming it into a phage-replicating unit, leveraging its ribosomes and ATP resources normally employed for the host’s benefit to its advantage [335]. Phage-specified proteins that are translated after the host cell infection from phage mRNA can reprogram these energetic pathways in the bacteria [335]. When the host cell is lysed, all of the bacteriophages are released into the environment, allowing them to infect a new host cell [333]. (2) A lysogenic cycle is very similar to the lytic, except that the phages replicate and pass themselves onto the bacterial daughter cells without killing the bacteria [333]. Bacteriophages cannot infect and replicate within human or animal cells; instead, they exclusively target bacterial cells [333].

The effectiveness of phages in the treatment of bacterial infections depends on factors such as the phage’s form and type, the level of lytic activity, and the method and timing of administration [336]. Different researchers have demonstrated that the use of phages over a long period has been effective in reducing *Salmonella* in the digestive tract [337]. The mode of administration includes oral administration by mixing with water or feed, spraying the surface of the eggs, or by the addition of bacteriophage suspension directly into infected products [338]. Henriques et al. demonstrated that administrating phages through aerosol spray during the transfer of eggs from incubators to hatchers can be a cost-effective and efficient method to reduce the horizontal transmission of *Salmonella* in poultry [339]. Bacteriophage treatment is a novel strategy for providing prophylactic treatment against poultry pathogens including *Salmonella* [340]. It can be used safely without altering the gut microbiota [341]. Wattana et al. demonstrated that the novel *Salmonella* phages showed a significantly high bacterial lysis effect (93.3%) on ciprofloxacin-resistant *Salmonella* strains in broilers [342].

Furthermore, Spricigo et al. demonstrated that the use of three *Salmonella*-phage cocktails (UAB_Phi 20, UAB_Phi78, and UAB_Phi87) showed two log reductions in pig skin and lettuce and one log reduction in *Salmonella* count in chicken breast contaminated with the bacteria [343]. In addition, the administration of five *Salmonella*-phase cocktails demonstrated an up to 3.1 log-reduction reduction in *Salmonella* count in contaminated raw chicken breast [344]. The complete list of bacteriophages used in treating *Salmonella* infection is shown in Table 3.

While phage therapy holds promise in combatting MDR pathogens like *Salmonella*, it does come with certain limitations [345]. These limitations include the phages’ narrow host spectrum, which restricts their effectiveness to specific bacterial genera [346], the potential development of bacterial resistance through CRISPR-Cas adaptive immunity against commonly encountered bacteriophages [347], and the lack of comprehensive data on the pharmacokinetic properties of these viruses [345]. Additionally, there are concerns about the adverse effects of bacterial toxins released during the phage-mediated lysis process [348]. These factors collectively challenge the widespread adoption and efficacy of phage therapy in clinical settings [349]. Despite these disadvantages, ongoing research and development are exploring ways to harness the potential of bacteriophages and use them against bacterial pathogens like *Salmonella* for both prophylactic and therapeutic purposes [350].

**Table 3:** Different studies for the evaluation of the efficacy of bacteriophages against *Salmonella* serotypes.

Phages	Target Serotypes	PFU/mL	Phase Application	Results	References
CNPSA1, CPNSA3, CNPSA4	*S.* Enteritidis PT4 P125589	10^11^	Single oral application of phage cocktail	Decrease in the occurrence of *S*. Enteritidis strains by 3.5 logs.	[337]
F1055S, F12013S	*S.* Enteriditis	2 × 10^2^	Phage isolated and applied by aerosol spray on fertile eggs	Around 58% and 76% reduction in the cecal and visceral *Salmonella* count, respectively, without any loss in the body weight compared to the control group.	[339]
Φ st1	*S.* Typhimurium and *S.* Hadar	10^12^	Intraclocal inoculation	*Salmonella* count reduced by 2.9 log10 CFU/mL within 6 h of challenge. *S.* Typhimurium had no trace of detection after 24 h.	[351]
SPGH1, SPGH3	*S.* Typhimurium	8.3 log_10_	Spotted	*S.* Typhimurium count was significantly reduced by 4.2 log_10._	[352]
UAB_Phi20, UAB_Phi78, UAB_Phi87	*S.* Enteriditis and *S*. Typhimurium	10^11^	Oral	Cecal *Salmonella* count significantly decreased by 5.3 log upon administration of three phage cocktails one day before or after bacterial infection.	[353]
φ10, φ25, φ151	*S.* Enteritidis P125109, Hadar 18, and Typhimurium 4/74	10^9−11^	Oral	Reduced cecal colonization by *S.* Enteritidis and *S.* Typhimurium by ≥4.2 log_10_ CFU and ≥2.9 log_10_ CFU, respectively.	[354]
Wide-Host-Range bacteriophages (WHR)	*S.* Enteritidis (SE), *S*. Typhimurium (ST)	10⁹	Sprayed with 5 mL of WHR and rinsed with sterile water	No bacteria were detected in two trials and a greater than 70% reduction was seen in the other two trials.	[355]
Bacteriophages of *S*. Typhimurium and *S*. enteritidis	*S.* Enteritidis (SE), and *S.* Typhimurium (ST)	1.18 × 10^11^–1.03 × 10^2^	Oral	Moderate decrease (1 log reduction) in *Salmonella* loads 3 days post-infection (dpi), with a greater reduction of 2 log at 5 dpi and complete clearance of the bacteria at 7 dpi.	[356]
ΦCJ07	*S.* Enteritidis (SE)	10^5^, 10^7^ 10^9^	Oral	After 3 weeks of treatment, no intestinal *Salmonella* was detected in 70% of hens treated with 10^9^ PFU/g of bacteriophage.	[357]
PSE5	*S.* Enteritidis (SE)	4 × 10^7^	Immersion	Three logs reduction in *Salmonella* count was observed after 30 min of phage treatment of the contaminated eggs.	[358]
Pu20	*S.* Pullorum	10^8^ or 10^9^	Direct inoculation	The phages demonstrated 1.06 and 1.12 log reduction in *S.* Pullorum in eggs stored at 4 °C and 25 °C, respectively.	[359]
UAB_Phi 20, UAB_Phi78, and UAB_Phi87	*S*. Enteritidis (SE), *S*. Typhimurium (ST)	10^9^ and 1 × 10^10^	Soaking in suspension and spraying	One log reduction in both *S*. Enteritidis and *S*. Typhimurium in chicken breast.	[343]
SEG5, SES8, STG2, STG5, and STS9	*S.* Enteritidis, *S.* Typhimurium	3 × 10^8^	Suspension added on the surface	*S.* Enteritidis and *S.* Typhimurium reduced by 3.06 and 2.21 log CFU/piece of chicken breast, respectively,	[344]
STGO-35-1	*S.* Enteritidis	4 × 10^6^	Direct addition	Significant reduction in *Salmonella* count (by 2.5 logs) in each piece of the chicken meat.	[360]

#### 4.5.5. Small Molecules and Quorum-Sensing Inhibitors

Small molecules (SMs) are low-molecular-weight compounds that can be directed to specific cellular processes of bacterial physiology to perform a broad to narrow spectrum of activity and can be used as growth inhibitors or virulence inhibitors [361]. These molecules can be obtained from natural sources or be synthesized [362,363,364]. The natural compounds may include phytochemicals, including fruit extracts (grape seed extract), plant extracts (punicalagin), spice oils (thyme, basil, rosemary, ginger, garlic), and phenolic compounds such as Gallo tannins, coumarins (furocoumarin), benzoates, and terpenes (monoterpenes, diterpenes, and triterpenes) [365,366,367]. The synthetic compounds may include furanone, chitosan, thiophene inhibitors, and limonene nanoemulsion [368,369]. Different studies evaluating the effect of small molecules on the growth and virulence of *Salmonella* are demonstrated in Table 4. For example, Li et al. demonstrated that using Quercitrin, a flavonoid with antioxidant properties, significantly inhibited the adhesion, invasion, and survival of *Salmonella* in HeLa cell lines by about 70% [370]. Similarly, Deblais et al. and Rajashekara et al., in two different independent experiments, demonstrated that the compounds imidazole and methoxybenzylamine were able to cause complete clearance of *S.* Typhimurium in vitro in Caco-2 cells with minimal toxicity to chicken RBC [371]. Furthermore, another study conducted by Jacob et al. demonstrated that compound 7955004 can cause 55% inhibition of preformed biofilms, with complete clearance of planktonic *S.* Typhimurium in vitro [372]. Similarly, Nagy et al. demonstrated an up to 2 log reduction in the colonization of *S.* Typhimurium in the spleen and liver 48 h post-infection in mice [373]. These compounds have demonstrated their effectiveness in disrupting crucial bacterial processes, including metabolism, virulence factor inhibition, and infection prevention, which positions them as a viable alternative to antibiotics for managing *Salmonella* infections [374]. Consequently, they provide a versatile and precisely targeted strategy for controlling this bacterium [375]. However, the intricate nature of *Salmonella*, its adaptability, and its potential to develop resistance to these molecules emphasize the imperative need for ongoing research and refinement of these compounds. In summary, small molecules possess the ability to inhibit the growth and virulence of *Salmonella* without the inherent risk of developing antibiotic resistance, thus positioning them as important candidates for the development of antibiotic-alternative therapeutics.

Quorum sensing (QS) is a bacterial cell-to-cell communication process that is regulated by the production, release, and uptake of particular signal molecules known as autoinducers (AI) [376]. It is a complex inter/intra-species process that allows a bacterium to carry out colony-wide functions such as biofilm formation, bioluminescence, sporulation, conjugation, and expression of the virulence factors [377,378,379]. Gram-negative bacteria like *Salmonella* produce autoinducers called autoinducer-2 (AI-2), a furanosyl borate diester that is found in both Gram-negative and Gram-positive bacteria [380]. Unlike Gram-positive bacteria, which use the *LuxI* gene that codes with the AHL molecule to activate the transcription of the *LuxICDBAE* operon to produce bioluminescence, *Salmonella* lacks the *LuxI* gene encoded in their genome [381]. *Salmonella* instead uses the *LuxR* homolog called *SdiA* to produce AI-2 molecules and regulate the expression of virulence genes [382]. These genes serve specific functions in facilitating host invasion and colonization, developing antibiotic resistance, evading complement systems, expressing fimbriae, and producing anti-phagocytic factors [383]. Therefore, the inhibition of *LuxS* and AI-2 activity can offer a promising strategy to decrease the virulence of *Salmonella* [384]. Quorum-sensing inhibitors (QSIs), which include small molecules, natural extracts, and oils, are increasingly being considered as promising alternatives to antibiotics for salmonellosis [385]. By disrupting quorum sensing, QSIs can effectively interfere with various colony-wide bacterial activities, including the formation of biofilms, the production of virulence factors, and the development of antibiotic resistance [385]. Unlike antibiotics, the QSIs inhibit the microbial quorum or prevent forming a quorum rather than exerting selection pressure and interfering with the cellular and metabolic process, thus making the bacteria less likely to develop resistance [386]. The QSIs were also reported to show high efficacy in vitro when combined with small molecules’ growth inhibitors [387]. Several studies have been conducted on compounds that can inhibit QS and AI-2 production by downregulating QS-associated genes and inhibiting the virulence of *Salmonella* (Table 4). For example, the use of punicalagin led to a reduction in motility and the downregulation of the QS-related genes *flhC*, *sdiA*, and *srgE* in *S.* Typhimurium; carvacrol, thymol, and eugenol downregulated the genes associated with host colonization, such as *flgG*, *fimD*, *sopB*, and *invH*, as well as genes related to macrophage survival like *ssaV* and *pipB* in *S.* Enteritidis; and furanone caused significant downregulation of QS-associated genes like *sdiA* and *srgE* in *S.* Typhimurium in vitro [388,389,390,391]. Similarly, dephostatin and homocysteine thiolactone are capable of downregulating QS-regulated *spiA* genes responsible for the adhesion and invasion of *Salmonella* to the host cells [390,392]. In addition, fluorothiazinon, fusaric acid, and cytosporone B have been found to inhibit the QS-regulated Type-3 secretion system apparatus, which is responsible for host cell adhesion and invasion [393,394,395]. In summary, QSIs offer significant promise as alternative strategies for managing *Salmonella* infections in humans and animals. Their capacity to interfere with bacterial communication, hinder the establishment of infections, evade host immune responses, and cause disease without impacting bacterial growth highlights their potential as valuable targets for developing antibiotic-alternative therapeutics to combat MDR *Salmonella* infections in both human and animal populations. However, it is essential to acknowledge the challenges associated with QSIs. Achieving high specificity to target a single bacterial species without affecting the normal microbiota can be challenging. Furthermore, our understanding of QS mechanisms in various pathogenic bacteria still needs to be broadened [396]. Ensuring the bioavailability and effective delivery of QSIs to complex environments like biofilms within the host body presents a significant hurdle. Additionally, the high cost and time required for QSI development pose practical challenges in their widespread use as therapeutic agents [397].

**Table 4:** Quorum sensing and small molecule inhibitors of *Salmonella* serotypes.

Small Molecules	Action	Target Strains	Concentration/Dose	Effect on Quorum Sensing Regulatory Process/Growth Inhibition	References
Punicalagin	QSI	*S.* Typhimurium	15.6 μg/mL	Downregulation of motility (*flhC*) and QS-associated genes (*sdiA* and *srgE*)	[388]
Carvacrol, Thymol, Eugenol	QSI	*S*. Enteritidis	0.5 mM, 0.5 mM, 1.2 mM	Significant downregulation of genes related to host colonization (*flgG*, *fimD*, *sopB*, *invH*, and TTSS genes) and macrophage survival (*ssaV* and *pipB*)	[389]
Furanone	QSI	*S.* Typhimurium 14028	500 uM	Downregulation of quorum-sensing regulatory genes targets *srgE* and *lsrA* of sdiA and AI-2 followed by downregulation of genes related to flagellar biosynthesis and biofilms	[390]
M-gallate	QSI	*S.* Typhimurium	128 μg/mL	Downregulation of QS-associated genes *sdiA* and *srgE* by 92.6 and 77.7% respectively.	[398]
Berberine	QSI	*S*. Typhimurium	0.019 mg/mL	Reduction in AI-2 production by 73.5% compared to the control with exogenously supplied C4-HSL reporter molecule	[399]
Tannic acids	QSI	*S.* Typhi *S.* Paratyphi	400 μg/mL	Drastically inhibited swarming motility, a major phenotype of quorum sensing without any impact on the growth of the bacteria	[400]
Xanthones	QSI	*S.* Typhimurium 21 SL1344	100 µM	A 60–70% inhibition in Ai-2 production, effective efflux pump inhibitors	[401]
*N*-(3-oxo octanoyl) DL-homoserine lactone	QSI	*S.* Typhimurium	10 nM	*SdiA* gene downregulation and inhibition of biofilm formation	[392]
Homocysteine thiolactone	QSI	*S.* Typhimurium	10 µM	Effect on *SdiA* gene expression with no effect on bacterial growth	[402]
Dephostatin	QSI	*S.* Typhimurium	100 µM	SPI-2 virulence genes inhibitor and restoring sensitivity to the colistin	[403]
Fluorothiazinon	QSI	*S*. Typhimurium	10 mg/kg	Suppression of Type-3 secretion system of *Salmonella* in vivo	[393]
Fusaric acid	QSI	*S*. Typhimurium	100 µM	Type-3 secretion system inhibitor with anti-invasion activity	[394]
INP0007 and INP0403	QSI	*S*. Typhimurium	100 μM	Inhibition of Type-3 secretion system 1-associated virulence and invasion.	[395]
Cytosporone B	QSI	*S.* Typhimurium	25 μM	Type-3 secretion system inhibition	[404]
Quercitrin	Growth inhibitors	*S.* Typhimurium	32 μg/mL	Reduction in *Salmonella* adhesion, invasion, and survival in the HeLa cell lines by 70%. Blocks effector SipA translocation important for the invasion of the host cells	[370]
Imidazole, Methoxybenzylamine	Growth inhibitors	*S.* Typhimurium	10 µM	Complete inhibition of growth and intracellular clearance of *Salmonella* from Caco-2, HD11, and THP-1 cell lines. Clearance of biofilm-embedded bacteria at 4 µM concentration	[371]
Compound 7955004	Growth inhibitors	*S.* Typhimurium 14028	5 μM	More than 55% inhibition of preformed biofilms Complete clearance of the planktonic bacteria	[372]
SM4 (Imidazole class) SM 5 (Methoxybenzylamine class)	Growth inhibitors	*S.* Typhimurium	MIC: 10 µM and 25 µM	Bactericidal effect on WT *S.* Typhimurium with minimal toxicity on eukaryotic cell models including Caco-2, HD11, chicken macrophage cell lines, sheep or chicken RBCs, and complete clearance of internalized bacteria	[371]

#### 4.5.6. Vaccines

Vaccines are preparations of antigens or a part of the pathogen which, when administered to a host, can safely induce an immune response against infection by specific pathogens upon future exposure, thereby preventing severe infections [405,406]. Vaccines mimic the natural infection without causing severe illness, enabling the body to build immunity against diseases, helping prevent infection, reducing the severity of the infection, and lowering the risk of complications and transmission to others [406]. Vaccines help reduce the incidence of infectious diseases worldwide including fowl (avian) cholera, coronavirus, anthrax, polio, norovirus, Rift Valley fever, and rabies [407,408,409,410,411]. The mode of action of vaccines depends on their formulations [412]. For example, (1) Live attenuated vaccines contain a weakened, live version of the pathogen, capable of eliciting an immune response without causing disease [413]. The purpose of attenuation is to eliminate pathogen’s infectivity but preserve their immunogenicity [414]. These vaccines can effectively stimulate humoral and secretory antibodies, as well as activate cytotoxic T-cells [415]; (2) Killed-whole-cell vaccines are the vaccines in which the pathogens are killed but maintain their epitope structures intact to preserve their immunogenicity, while simultaneously eliminating their capacity to replicate or cause disease [416]; (3) Toxoid vaccines are a type of vaccine in which a pathogen’s toxin is purified and subjected to formalin treatment to deactivate its harmful effects but retains its immunogenicity against the associated pathogen [417]; (4) Subunit vaccines are a class of vaccines which contain fragments of the pathogen, such as polysaccharides, nucleic acids, or proteins like flagellin or synthetic peptides [418]; (5) Outer membrane vesicle (OMV) vaccines are made up of naturally discharged constituents of OMVs from the bacterial outer membrane, featuring vital antigenic elements capable of eliciting an immune response without triggering any illness [419]; (6) Protein-polysaccharide conjugate vaccines consist of bacterial polysaccharides which are bound to proteins that enable a desired immune response [420]. These vaccines induce polysaccharide-specific B-cell immunity, help prevent colonization, and block person-person transmission, thus generating herd immunity [421]; (7) Recombinant viral and bacterial vector vaccines employ non-pathogenic viruses or bacteria as vectors to deliver genetic information encoding the antigens of the pathogen into the host cells, which subsequently elicits the immune response [422]; (8) Nanovaccines are an innovative class of vaccines that employ nanoparticles (NPs) as carriers or adjuvants. These nanoparticles enable precise targeting of the specific site where the disease originated, distinguishing them from vaccines that exert systemic effects [423].

Currently, two different forms of vaccines are widely available to control typhoid and paratyphoid fever in humans [424]. These vaccines are available in the form of orally administered live-attenuated Ty21a vaccine and injectable Vi capsular polysaccharide vaccine [425]. Ty21a vaccine has an efficacy of 51% in adults and children above 5 years with high cross-protection against both *S.* Typhi and *S.* Paratyphi B [426]. The vaccine has been reported to develop IgA antibody response and mediate CD4^+^ T-cell mediated antibody-dependent cellular cytotoxicity against *S.* Typhi and *S.* Paratyphi [427]. Antibody-dependent enhanced phagocytosis of *S.* Typhi has also been reported in orally administrated Ty21a vaccines [428]. MacLennan et al. and Wahid et al. have separately reported robust production of T-cell-mediated immune stimulation, with increased production of IgA antibodies. They have also reported cross-reactive immunity against *S.* Typhi (56%) and *S.* Paratyphi (38%) [424,429]. Different from Ty21a, the Vi capsular polysaccharide vaccine acts through the activation of T-cell-independent IgG antibody production and has an efficacy of 55%, with cumulative immunity up to 3 years through a single dose [430]. The administration of a *S.* Typhi Vi polysaccharide with the tetanus toxoid conjugate vaccine (Tybar) vaccine has shown to have an efficacy of up to 85% in children under the age of 2 years, with a high increase in T-cell-independent IgG production [431]. Similarly, another Vi-polysaccharide based Vi Conjugate (Vi-CRM_197_) and Vi Conjugate (Vi-rEPA) vaccine has demonstrated up to 90% efficacy in children and a similar increase in IgG production lasting up to 4 years post-vaccination against *S.* Typhi [432,433]. In addition to this, other forms of vaccines are also available or are currently in development (Table 5). For example, Lyon et al. found that the administration of a single-dose independently attenuated deletion *S.* Typhi (Ty2ΔaroCΔssaV) ZH9 vaccine produced complete fecal clearance of *S.* Typhi 7 days post-infection, with rapid and high production of *S.* Typhi-specific IgG and IgA production [434]. Similarly, a new formulation of vaccines, Generalized Modules for Membrane Antigens (GMMA), containing bacterial surface immunogens such as LPS, has shown increased production and stimulation of peripheral blood mononuclear cells and elicit production of strong bacteriocidal anti-LPS O-antibody and IgG antibody with complete clearance of *S.* Typhimurium, *S.* Typhi, and *S.* Paratyphi A [37,40]. Furthermore, the modified live *S.* Dublin vaccine (EnterVene-d) has shown increased cell-mediated, humoral, and mucosal immunity against *S.* Dublin in cattle with antibody titer, increased by 49% in vaccinated cows and by 88.56% in calves from the vaccinated cows, demonstrating substantial horizontal transfer [435]. Several vaccines have been developed to prevent *Salmonella* infections in poultry. Renu et al. have demonstrated that the oral administration of a chitosan-adjuvanted *Salmonella* subunit nanoparticle vaccine containing outer membrane proteins (OMPs), and flagellins (F) coated with nanoparticles (NPs) can cause significant stimulation of gut mucosal immunity upregulating TLRs, Th1, and Th2 cytokine mRNA, with increased production of OMPs-specific IgY and IgA antibodies in saliva and intestine [436]. Similarly, the commercially available modified-live *S*. Typhimurium vaccine (Poulvac^®^ ST; Zoetis Inc., Parsippany, NJ, USA), one of the most effective poultry vaccines commercially available, has demonstrated an up to 50% reduction in *S.* Kentucky, *S.* Enteritidis, *S.* Heidelberg, *S.* Typhimurium, and *S*. Hadar in the liver and spleen [437]. Another trivalent but inactivated *Salmonella* enterica vaccine (Nobilis^®^ Salenvac T; Intervet International B.V., Boxmeer, The Netherlands) has shown an up to 3.9 log increase in mean antibody titer after the administration of the booster dose in chicken, along with a 2.6 log reduction in cecal shedding of *S.* Typhimurium and *S.* Enteritidis, followed by a 1.3 log reduction in *S*. Infantis [438]. Unfortunately, due to the presence of variations in the cellular structures and antigens between the typhoidal and non-typhoidal *Salmonella* species, the technology used in typhoid vaccine development has not benefited the development of a vaccine against non-typhoidal *Salmonella* species [430].

While vaccines have played a pivotal role in mitigating the severity of infections, progress in the development of vaccines against *Salmonella* has been limited. One of the primary challenges is the extensive genetic variability among *Salmonella* serovars, each possessing distinct surface antigens. This variability poses a significant obstacle to creating effective *Salmonella* vaccines [439]. Additionally, various other factors contribute to the difficulty faced in *Salmonella* vaccine development [413]. These include the risk of vaccine failure due to improper handling, the potential for live-attenuated vaccines to regain virulence after administration, the development of tolerance to toxoids when high doses are used in toxoid vaccines, and the relatively low immunogenicity of outer membrane vesicle vaccines [440]. These combined factors collectively have limited the progress in the field of *Salmonella* vaccine development [441]. Considering these limitations, it is crucial to emphasize the necessity of ongoing research in vaccine development. Vaccines have undeniably established themselves as fundamental pillars of public health, offering substantial advantages in preventing and mitigating infectious diseases. The efforts should focus on creating more effective vaccines and addressing the complexities associated with specific pathogens, variable immune responses, and the ever-evolving nature of infectious diseases. In doing so, we can harness the full potential of vaccines as vital tools in safeguarding public health, while simultaneously working to overcome their constraints.

**Table 5:** Commercially available vaccines against *Salmonella* in humans and animals.

Vaccines	Target Pathogens	Indications	Notable Observations	References
Vi Conjugate (Vi-rEPA)	*S.* Typhi	Human	Up to 90% efficacy in children between 2 and 5 years old. Rapid production of Vi-specific IgM and IgG with 2 logs reduction in shedding of the bacteria.	[432]
Modified live *S.* Dublin vaccine (EnterVene-d)	*S.* Dublin	Cattle	Stimulated cell-mediated immunity with antibody titer increased by 49% in vaccinated cows. Antibody titer increased by 88.56% in calves from the vaccinated cows, demonstrating strong horizontal transfer.	[435]
Ty21a	*S.* Typhi and *S.* Paratyphi B	Human	Cross-reactive multifunctional T-cell response with an increase in IgA production of 56% against *S.* Typhii and 38% against *S.* Paratyphi B compared to the control.	[424,429]
M01ZH09, Single dose independently attenuating deletion (*S.* Typhi (Ty2Δ*aroC*Δ*ssaV*) ZH9)	*S.* Typhi	Human	Rapid and high production of IgG and IgA with the fecal clearance of the bacteria within 7 days post-infection without any severe symptoms.	[434]
GMMA, Generalized Modules for Membrane Antigens	*S*. Typhimurium, *S.* Typhi, *S.* Paratyphi A	Human	Increased stimulation of peripheral blood mononuclear cells with increased IL-6 production. Elicit strong bacteriocidal anti-LPS O-antigen antibody and IgG production and complete clearance of the bacteria.	[37,40]
Vi Conjugate (Vi-CRM_197_)	*S.* Typhi	Human	Demonstrated 89% protective efficacy against typhoid fever and the protection lasted at least 4 years, significantly increased IgG antibody titer.	[433]
*S.* Typhi Vi polysaccharide tetanus toxoid conjugate vaccine (Tybar)	*S.* Typhi	Human	Robust anti-Vi IgG response in all age groups with significant protection across all age groups, including infants (children under the age of 2 years), with an efficacy of 85% without any side effects.	[431]
AviPro Megan Vac 1 + A12:E13	*S*. Typhimurium, *S*. Enteritidis and *S.* Heidelberg	Poultry	Complete clearance of *S.* Enteritidis by 10 days post-infection with positive cases reduced to 6% on secondary inoculation. No vertical transfer of the antibodies observed.	[442]
Chitosan-adjuvanted *Salmonella* subunit nanoparticle vaccine (OMPs-F-CS NPs)	*S.* Enteritidis	Poultry	Upregulation of TLRs and Th1 and Th2 cytokine mRNA with increased OMPs-specific IgY and IgA antibodies in saliva and intestine on oral administration. *Salmonella* shedding was reduced by 7 times compared to the mock challenge.	[436]
Inactivated trivalent *Salmonella enterica* vaccine (Nobilis^®^ Salenvac T; Intervet International B.V., Boxmeer, The Netherlands)	*S.* Typhimurium, *S.* Enteritidis and *S.* Infantis	Poultry	A 3.9 log increase in mean antibody titer upon administration of the booster dose in chicken with 2.6 log reduction in cecal shedding of *S.* Typhimurium and *S.* Enteritidis, followed by 1.3 log reduction in *S*. Infantis	[438]
Poulvac^®^ ST (Zoetis Inc. New Jersey, USA)	*S.* Typhimurium, *S.* Kentucky, *S*. Enteritidis, *S.* Heidelberg and *S*. Hadar	Poultry	A % reduction in *S.* Kentucky, *S.* Enteritidis, *S.* Heidelberg, *S.* Typhimurium, and *S.* Hadar in liver and spleen, with cross-protection between all 5 strains.	[437]
Autologous killed trivalent vaccine (Tri-Vaccine)	*S*. Typhimurium, *S*. Enteritidis and *S.* Heidelberg	Poultry	In total, 58% of the cloacal swabs from the infected birds demonstrated complete clearance of the bacteria 8 days post-infection.	[442]

#### 4.5.7. Organic Acids

Organic acids (OAs) are acidic organic compounds, primarily consisting of short-chain fatty acids (SCFAs) and medium-chain fatty acids (MCFAs) [443]. Organic acids are typically produced by the native gut microbiota residing in the intestines of animals, as well as in crops and within the ceca of birds [444]. These compounds exert antimicrobial activities, like restraining the growth and colonization of pathogenic bacteria in the gut, by reducing the pH within microbial cytoplasm, disrupting energy production and regulation, and causing the accumulation of dissociated acid ions to toxic levels inside microbial cells [445,446]. This improves digestibility, gut health, and immunity [447]. They also hold significant importance in the animal production industry [448]. Traditionally, they have been employed as fungistats in the animal food industry and have demonstrated strong antibacterial properties against foodborne pathogens such as *Salmonella* [449]. They are primarily used in the form of salts, either monocarboxylic acids like acetic, butyric, formic, and propionic acids, or based on the side chains available [450].

The short-chain fatty acids encompass organic acids like formic acid, acetic acid, propionic acid, and butyric acid, along with acids containing additional hydroxyl (OH) groups such as citric, lactic, malic, and tartaric acids [451], whereas, the medium-chain fatty acids include organic acids like caproic acid, caprylic acid, capric acid, and lauric acid [452,453]. Several studies have indicated that MCFAs can have more potent effects compared to SCFAs, but it is important to distinguish between their bactericidal and bacteriostatic activities [199]. For example, caprylic acids and capric acids, which are known as MCFAs, exhibited bactericidal properties and completely cleared *Salmonella* Enteritidis in vitro compared to other organic acids at the same concentration [454]. Similarly, MCFAs C6 and C10 had bacteriostatic effects on *S.* Enteritidis at a concentration of 25 mM, whereas the same strain showed complete resistance to 100 mM of SCFAs [455].

Organic acids can significantly impact the growth of and colonization by enteric pathogens like *Salmonella* within the host and in the host’s feed products [456]. Koyuncu et al. demonstrated a significant reduction (up to 2.5 log) in the numbers of *S. Infantis*, *S. Putten*, *S. Senftenberg*, and *S. Typhimurium* in mash and rapeseed feed containing formic acid, propionic acid, and sodium formate. Moreover, the combination of propionic acid and sodium formate was more efficacious [457]. Similarly, dietary supplementation of a combination of organic acid mixture of formic acids and sodium formate in broiler chicken showed a significant (1.5 logs) reduction in the cecal colonization of *S.* Typhimurium [458]. Furthermore, Ruhnke et al. also demonstrated that the use of formic acids (1 kg/ton), or propionic acid (5 kg/ton) as feed additives in broiler chicken can cause low cecal retention (35%) of *S.* Typhimurium compared to the control group (60%) at 6 weeks of age [459]. Moreover, a significant reduction (up to 90%) in fecal *Salmonella* shedding in pigs was observed 7 days post-exposure after supplementation with sodium butyrate, formic, and citric acid as acidifiers [460]. Additionally, the normal gut microflora and oral supplementation of probiotics and prebiotics was shown to stimulate the production of short-chain fatty acids in poultry GIT, thus limiting *Salmonella* colonization, causing complete clearance of the bacteria and modulation of gut immunity in mice [461]. It was also demonstrated that lactic acid bacteria can work synergistically in lowering the gut pH and modulating the gut immunity in 160-day-old broiler chicken [462].

While organic acids have shown promise in antimicrobial drug development and pathogen inhibition, notable concerns and limitations exist associated with their utilization [463]. The most significant limitation is the dissociation of the organic acids and their ability to reach the lower portion of the gastrointestinal tract [464]. The organic acids are digested and metabolized, and, as a result, the concentration is decreased, leading to dissociation when reaching the lower GIT, which is the primary site for *Salmonella* infection. Resistance is also possible as pathogenic species adapt to treatment [450]. Despite the limitations, it is worth noting that organic acids, indeed, are promising tools for mitigating *Salmonella* contamination in food. Their application should be considered an integral component of a comprehensive food safety strategy aimed at controlling and preventing foodborne infections, particularly those caused by antibiotic-resistant *Salmonella*.

#### 4.5.8. Essential Oils (EOs)

Essential oils (EOs), also referred to as volatile oils, are a mixture of aromatic compounds with characteristic flavors and aromas [465]. They are derived from various plant parts, including stems, flowers, fruits, buds, leaves, and even wood [466]. Essential oils consist of a wide range of compounds such as alcohol, acetones, phenolic acids, terpenes, aldehydes, and esters, which can play a significant role as antimicrobial agents or nutrient supplements [221]. For example, citrus based EOs comprises more than 2000 different types of organic compounds [466,467]. Essential oils exist in a highly bioactive vapor phase and typically do not require physical contact with the pathogen to demonstrate antimicrobial action [468]. In plants, they also play significant role in protecting against bacterial, viral, or fungal infections and help attract insects that can directly help in the pollination process [469,470]. Essential oils possess numerous therapeutic properties for humans and animals, including antimicrobial, antioxidant, anticancer, antidiabetic, spasmolytic, and insect repellent. Moreover, they have long been utilized in aromatherapy to promote relaxation, stabilize moods, and provide physical and psychological relief [471]. These compounds are regularly used in the food industry as preservatives for preventing the growth of foodborne bacteria such as *Salmonella*, *E. coli*, *Listeria*, and *Campylobacter* [472].

Essential oils have been used as antimicrobial compounds and food preservatives to control *Salmonella* [473]. It has been reported that EOs like cinnamon oils result in a 2.7 log reduction in *S.* Typhimurium and can have synergistic effects in vitro when used with antibiotics such as cefotaxime [474]. Similarly, lemongrass, cinnamon, geraniol, and palmarosa-based EOs against can cause complete clearance of *S.* Enteritidis in fruit juices [475]. Furthermore, in vitro, assessment of the antimicrobial effect of *C. zelanicum* and *S. aromaticum* against the *Salmonella enterica* serotypes Enteritidis and *S.* Typhimurium from poultry demonstrated a high inhibitory effect, with MIC ranging from 1.26 mg/mL to 0.63 mg/mL for *C. zelanicum* and 2.637 mg/mL to 0.164 mg/mL for *S. aromaticum* and an MIC of 1.289 mg/mL to 0.322 mg/mL for the mixture of both [476]. However, laurel leaves, cardamom, ginger, and rosemary based EOs had moderate inhibitory activity against different *Salmonella* serotypes isolated from humans [477]. Similarly, another in vitro study showed that thyme oil had the highest inhibitory effect (22.5–38.5 mm zone of inhibition) against *S.* Enteritidis, *S.* Montevideo, and *S.* Heidelberg, followed by clove oil and rosemary oil, whereas orange oil had no significant inhibitory effect on *S.* Heidelberg [478]. Also, oregano, thyme, clove, and arborvitae based EOs showed significant inhibitory effects (*p* < 0.001), with complete clearance of *S.* Typhimurium at 0.125% with no genotoxic effect on human embryo lung cells after 24 h of administration in vitro [479]. Different studies on evaluating the effect of EOs on the growth of *Salmonella* are shown in Table 6. 

While EOs have found diverse applications, ranging from feed additives and crop protectants to food preservatives and treatments for human ailments, it is essential to exercise caution when considering their extended utilization due to the potentially toxic effects of these compounds [480]. The widespread use of essential oils has been hindered by their adverse impact on organoleptic characteristics, limited stability under standard environmental conditions, volatility, poor solubility in water, and possible toxicity [481]. Toxicological concerns related to essential oils have been documented, including findings by Millet et al. who reported instances of neurotoxicity and tonic-clonic convulsions in humans due to the use of commercially available extracts from sage, hyssop, thuja, and cedar [482]. Similarly, severe effects, including dermatitis, hospitalizations, and, in severe cases, fatalities have also been reported following aromatherapy using lavender, peppermint, and ylang-ylang [483]. Looking forward, the integration of EOs into a comprehensive food safety strategy demands a holistic and multidisciplinary approach, and further research is essential for their production to guarantee uniform quality, efficacy, and safety.

**Table 6:** Different organic acids used for the control of *Salmonella* infection in humans and animals.

Plant	Major Components	*Salmonella* Serotype	MIC	Activity	References
*Thymus vulgaris*	Thymol (37.5%), p-cymene (14.49%), γ-terpinene (11.15%), linalool (4.71%), and carvacrol (4.62%)	*S.* Typhimurium ATCC 14028	0.25% *v*/*v*	The zone of inhibition in the agar-well diffusion assay was found to be 25.5 mm against *S.* Typhimurium, with complete clearance of the bacteria at MIC 0.25% *v*/*v*	[484]
*Origanum vulgare*	Thymol- and carvacrol-based EO	*S.* enteritidis ATCC 13076	120 μg/mL (carvacrol), 130 μg/mL (Thymol)	Complete clearance of the bacteria at 120 μg/mL (carvacrol) and 130 μg/mL (Thymol) in vitro	[485]
*Pistacia atlantica* subsp. Kurdica	α-Pinene (10.8%)	*S.* Typhimurium ATCC 14028	0.26 mg/mL	Complete clearance of *S*. Typhimurium was found to be at 0.5 mL/mL with a zone of inhibition of 22 mm	[486]
*Cinnamomum verum*	Not identified	*S*. enteritidis, *S*. Typhimurium, *S*. Heidelberg	>20 μL/mL	The zone of inhibition of all of the strains was found to be higher than 20 mm on agar well diffusion assay.	[487]
*Citrus medica* L. Var. *Sarcodactylis*	d-Limonene terpinene	*S.* Typhimurium	2.0 mg/mL	The zone of inhibition was found to be 20 mm and the inhibition of biofilm formation was found to be 90%.	[58]
*Ocimum basilicum*	linalool, 1,8-cineole, eugenol, α-terpineol, ρ-cymene, and germacrene D	*S.* Enteritidis	20 μg/mL	Two log reduction in the number of *Salmonella* when used in food products, colonization resistance was evident	[488]
*Allium sativum* (Garlic)	diallyl disulfide	*S.* Typhimurium	MIC/8 (1/512) μg/mL	Inhibition of biofilm formation by 23%, downregulation of virulence genes including *invA* and *sdiA* genes	[489]
Commercially available Essential oils.	Thymol, carvacrol, cinnamaldehyde	*S.* Enteritidis	4.6 mg/mL	Complete clearance of illeal and cecal *Salmonella* in broiler chicken at 10 dpi, improved illeal integrity, gut immunity modulation	[33]
*Aniba rosaeodora*	Linaloo	*S.* Typhimurium *S.* Pullorum	4 mg/mL 8 mg/mL	In vitro: complete clearance of the bacteria at 4 mg/mL and 8 mg/mL, respectively In vivo: complete clearance and systemic protection in chicks, modulate host inflammatory process	[490]
*Cymbopogon citrates* (Lemongrass)	Neral, Citral, Geranyl acetate	*S.* Newport	---	Significant reduction in bacterial population by 1 log CFU/g when co-cultured with *S.* Newport	[491]

## 5. Conclusions

Foodborne pathogens such as *Salmonella* pose a formidable challenge to human and animal health and significant economic loss in the healthcare and agricultural sectors worldwide. The widespread use of antibiotics in food animals for growth promotion, therapeutic applications, and preventative measures has played a significant role in the swift rise and global dissemination of AMR *Salmonella*. Furthermore, the indiscriminate use of antibiotics, particularly in sub-therapeutic doses, has significantly contributed to the rise of MDR strains. The rapid emergence and spread of MDR *Salmonella* have necessitated the development of alternative strategies for treating and controlling the infection. Addressing the issue of AMR strains calls for a multifaceted strategy involving early detection of infections and the implementation of necessary biosecurity measures to effectively contain outbreaks within the infected individual or the animal farm. Early detection of the infection allows for the timely initiation of appropriate targeted treatment and avoids the need for broad-spectrum antibiotics, which lowers the selective pressure that drives the development and spread of AMR strains in a bacterium. In addition to the early detection of the outbreak, the use of antibiotic-alternative therapeutics holds a promising role in combating AMR strains. Several research efforts have been made to limit the spread of AMR *Salmonella* by exploring various alternative intervention strategies, including the use of probiotics and prebiotics, antimicrobial peptides, phage therapy, small molecule growth inhibitors, quorum sensing/virulence inhibitors, vaccines, organic acids, and essential oils. These strategies can be used individually or in combination.

Nonetheless, each alternative approach carries its distinct advantages and limitations. It is crucial to approach them carefully, and additional research is required to ascertain their effectiveness, safety, and viability when implemented on a larger scale. As we transition to a future where traditional treatments like antibiotics could progressively lose efficacy, adopting a contemporary and enhanced strategy is crucial. These strategies involve synergistic application of these antibiotic alternatives, complemented by enhanced biosecurity protocols and judicious antibiotic use. Such a comprehensive approach will prove pivotal in controlling the dissemination of AMR-*Salmonella* infection. Furthermore, applying a One Health approach and continued collaboration between researchers, healthcare professionals, veterinarians, and policymakers in antibiotic stewardship is crucial in safeguarding public health and food safety from the threat of prevalent foodborne pathogens like MDR *Salmonella*.

## Figures and Tables

**Table 1:** *Salmonella* outbreaks in the US through the last 10 years and their source according to CDC.

Year	Number of Outbreaks	Number of Illnesses	Identified Serotypes	Source
2012	9	1217	Bredeney, Braenderup, Typhimurium, Newport, Enteritidis, Bareilly, Nchanga, Hadar, Infantis, Newport, Lille	Peanut butter, mangoes, cantaloupe, ground beef, raw scraped ground tuna product, hedgehogs, live poultry
2013	9	2278	Sandiego, Pomona, Poona, Heidelberg, Montevideo, Mbandaka, Saintpaul, Typhimurium, Infantis, Lille, and Newport	Small turtles, foster farms brand chicken, tahini sesame paste, cucumber, ground beef, live poultry
2014	8	429	Cotham, Heidelberg, Stanley, Typhimurium, Newport, Hartford, Oranienburg, and Braenderup	Bearded dragons, chicken, organic sprouted chia powder, nut butter, raw cashew cheese, frozen rodent feed, cucumbers
2015	8	1512	Enteritidis, Paratyphi B variant L (+) tartrate (+), Weltevreden, Sandiego, Poona, Hadar, Indiana, and Muenchen.	Bean sprouts, raw sprouted nut butter spreads, cucumbers, raw, frozen, stuffed chicken entrees, frozen raw tuna, live poultry, and small turtles
2016	5	114	Oranienburg, Reading, Abony, Montevideo, Senftenberg, Muenchen, Kentucky, Virchow, and Heidelberg.	Shell eggs, alfalfa sprouts, pistachios, organic shake and meal products, dairy calves, and live poultry
2017	5	2171	Agbeni, and Typhimurium	Pet turtles, live poultry, laboratory exposure.
2019	9	1632	Javiana, Dublin, Uganda, Concord, Carrau, Schwarzengrund, Oranienburg, Typhimurium	Cut fruit, ground beef, papayas, kawaran brand tahini, pre-cut melon, butterball, brand ground turkey, pet turtles, backyard poultry, and hedgehogs
2020	8	3107	Stanley, Enteritidis, Newport, Muenster, Typhimurium, Hadar	Wood ear mushrooms, peaches, onions, pet bearded dragons, pet hedgehogs, backyard poultry, and small pet turtles
2021	10	2575	Thompson, Oranienburg, Typhimurium, Weltevreden, Infantis, Enteritidis, Hadar	Seafood, pet turtles, Italian-style meats, onions, prepackaged salads, frozen cooked shrimp, raw frozen breaded stuffed chicken products, cashew brie, ground turkey, backyard poultry, wild songbirds
2022	7	1469	Typhimurium, Litchfield, Senftenberg, Stanley, and Uganda	Alfalfa sprouts, fish, peanut butter, pet bearded dragons, small turtles, poultry
2023	8	1527	Enteritidis, Thompson, Saint Paul, and Infantis	Raw cookie dough, flour, ground beef, fresh diced onion, cantaloupes, small turtles, dry dog food, and poultry

## Data Availability

Data are contained within the article.

## References

[1] Center for Disease Control and Prevention (CDC) *Salmonella* Infection. https://www.cdc.gov/healthypets/diseases/salmonella.html.

[2] Sanchez-Vargas F.M., Abu-El-Haija M.A., Gomez-Duarte O.G. (2011). *Salmonella* infections: An update on epidemiology, management, and prevention. Travel Med. Infect. Dis..

[3] Wiedemann A., Virlogeux-Payant I., Chaussé A.-M., Schikora A., Velge P. (2015). Interactions of Salmonella with animals and plants. Front. Microbiol..

[4] Helmy Y.A., El-Adawy H., Abdelwhab E.M. (2017). A Comprehensive Review of Common Bacterial, Parasitic and Viral Zoonoses at the Human-Animal Interface in Egypt. Pathogens.

[5] Baptista D., Borsoi A., Reischak D., Nascimento A., Montesino L., Camillo S., Abreu D., Pereira V. (2023). Salmonella Serovars Isolated from Poultry Breeding Flocks under the Brazilian Official Control Programme Between 2016 and 2018. Braz. J. Poult. Sci..

[6] Balasubramanian R., Im J., Lee J.S., Jeon H.J., Mogeni O.D., Kim J.H., Rakotozandrindrainy R., Baker S., Marks F. (2019). The global burden and epidemiology of invasive non-typhoidal Salmonella infections. Hum. Vaccines Immunother..

[7] Hoffmann S., Batz M.B., Morris J.G. (2012). Annual Cost of Illness and Quality-Adjusted Life Year Losses in the United States Due to 14 Foodborne Pathogens. J. Food Prot..

[8] Chlebicz A., Śliżewska K. (2018). Campylobacteriosis, salmonellosis, yersiniosis, and listeriosis as zoonotic foodborne diseases: A review. Int. J. Environ. Res. Public Health.

[9] Center for Disease Control and Prevention (CDC) Salmonella. https://www.cdc.gov/salmonella/index.html#:~:text=CDC%20estimates%20Salmonella%20bacteria%20cause,%2C%20fever%2C%20and%20stomach%20cramps.

[10] Center for Disease Control and Prevention (CDC) Drug-Resistant Nontyphoidal Salmonella. https://www.cdc.gov/drugresistance/pdf/threats-report/nt-salmonella-508.pdf.

[11] National Institute of Allergy and Infectious Diseases (NIAID). https://www.niaid.nih.gov/research/emerging-infectious-diseases-pathogens.

[12] Wei X., Long L., You L., Wang M., Wang D., Liu C., Li S., Wang J. (2023). Serotype distribution, trend of multidrug resistance and prevalence of β-lactamase resistance genes in human Salmonella isolates from clinical specimens in Guizhou, China. PLoS ONE.

[13] Keestra-Gounder A.M., Tsolis R.M., Bäumler A.J. (2015). Now you see me, now you don’t: The interaction of Salmonella with innate immune receptors. Nat. Rev. Microbiol..

[14] Hoelzer K., Moreno Switt A.I., Wiedmann M. (2011). Animal contact as a source of human non-typhoidal salmonellosis. Vet. Res..

[15] Bula-Rudas F.J., Rathore M.H., Maraqa N.F. (2015). *Salmonella* Infections in Childhood. Adv. Pediatr..

[16] Crump J.A., Sjolund-Karlsson M., Gordon M.A., Parry C.M. (2015). Epidemiology, Clinical Presentation, Laboratory Diagnosis, Antimicrobial Resistance, and Antimicrobial Management of Invasive *Salmonella* Infections. Clin. Microbiol. Rev..

[17] Giannella R.A., Baron S. (1996). Salmonella. Medical Microbiology.

[18] Cohen J.I., Bartlett J.A., Corey G.R. (1987). Extra-intestinal manifestations of salmonella infections. Medicine.

[19] Wibisono F.M., Wibisono F.J., Effendi M.H., Plumeriastuti H., Hidayatullah A.R., Hartadi E.B., Sofiana E.D. (2020). A Review of Salmonellosis on Poultry Farms: Public Health Importance. Syst. Rev. Pharm..

[20] World Health Organization (WHO) Salmonella (Non-Typhoidal). https://www.who.int/news-room/fact-sheets/detail/salmonella-(non-typhoidal).

[21] Olnood C.G., Beski S.S., Choct M., Iji P.A. (2015). Use of *Lactobacillus johnsonii* in broilers challenged with Salmonella sofia. Anim. Nutr..

[22] Tie K., Yuan Y., Yan S., Yu X., Zhang Q., Xu H., Zhang Y., Gu J., Sun C., Lei L. (2018). Isolation and identification of Salmonella pullorum bacteriophage YSP2 and its use as a therapy for chicken diarrhea. Virus Genes.

[23] Sania T., Abdul S., Muhammad H., Areeb A., Ayesha M., Shehroz A., Abdelslam Masoud Abobakr A. (2022). Salmonella in Poultry; an Overview. Int. J. Multidiscip. Sci. Arts.

[24] Velasquez C.G., Macklin K.S., Kumar S., Bailey M., Ebner P.E., Oliver H.F., Martin-Gonzalez F.S., Singh M. (2018). Prevalence and antimicrobial resistance patterns of Salmonella isolated from poultry farms in southeastern United States. Poult. Sci..

[25] Holschbach C.L., Peek S.F. (2018). *Salmonella* in Dairy Cattle. Vet. Clin. Food Anim. Pract..

[26] Chen H.-M., Wang Y., Su L.-H., Chiu C.-H. (2013). Nontyphoid *Salmonella* Infection: Microbiology, Clinical Features, and Antimicrobial Therapy. Pediatr. Neonatol..

[27] Galán J.E. (2021). Salmonella Typhimurium and inflammation: A pathogen-centric affair. Nat. Rev. Microbiol..

[28] Stoycheva M.V., Murdjeva M.A. (2006). Antimicrobial therapy of salmonelloses—Current state and perspectives. Folia Medica.

[29] Jibril A.H., Okeke I.N., Dalsgaard A., Olsen J.E. (2021). Association between antimicrobial usage and resistance in Salmonella from poultry farms in Nigeria. BMC Vet. Res..

[30] Ashurst J.V., Truong J., Woodbury B. (2022). Salmonella Typhi. StatPearls.

[31] Hailu W., Helmy Y.A., Carney-Knisely G., Kauffman M., Fraga D., Rajashekara G. (2021). Prevalence and Antimicrobial Resistance Profiles of Foodborne Pathogens Isolated from Dairy Cattle and Poultry Manure Amended Farms in Northeastern Ohio, the United States. Antibiotics.

[32] Van Panhuis W.G., Grefenstette J., Jung S.Y., Chok N.S., Cross A., Eng H., Lee B.Y., Zadorozhny V., Brown S., Cummings D. (2013). Contagious diseases in the United States from 1888 to the present. N. Engl. J. Med..

[33] Sears K.T., Galen J.E., Tennant S.M. (2021). Advances in the development of Salmonella-based vaccine strategies for protection against Salmonellosis in humans. J. Appl. Microbiol..

[34] Miletic S., Goessweiner-Mohr N., Marlovits T.C. (2020). The structure of the type III secretion system needle complex. Curr. Top Microbiol. Immunol..

[35] Gordon M.A., Graham S.M., Walsh A.L., Wilson L., Phiri A., Molyneux E., Zijlstra E.E., Heyderman R.S., Hart C.A., Molyneux M.E. (2008). Epidemics of invasive *Salmonella enterica* serovar enteritidis and *S. enterica* Serovar typhimurium infection associated with multidrug resistance among adults and children in Malawi. Clin. Infect. Dis..

[36] Milligan R., Paul M., Richardson M., Neuberger A. (2018). Vaccines for preventing typhoid fever. Cochrane Database Syst. Rev..

[37] Giannelli C., Cappelletti E., Di Benedetto R., Pippi F., Arcuri M., Di Cioccio V., Martin L.B., Saul A., Micoli F. (2017). Determination of free polysaccharide in Vi glycoconjugate vaccine against typhoid fever. J. Pharm. Biomed. Anal..

[38] De Benedetto G., Salvini L., Gotta S., Cescutti P., Micoli F. (2018). Investigation on Sugar–Protein Connectivity in Salmonella O-Antigen Glycoconjugate Vaccines. Bioconjugate Chem..

[39] Maddux J.T., Stromberg Z.R., Curtiss III R., Mellata M. (2017). Evaluation of Recombinant Attenuated Salmonella Vaccine Strains for Broad Protection against Extraintestinal Pathogenic Escherichia coli. Front. Immunol..

[40] Mancini F., Micoli F., Necchi F., Pizza M., Berlanda Scorza F., Rossi O. (2021). GMMA-Based Vaccines: The Known and The Unknown. Front. Immunol..

[41] Łojewska E., Sakowicz T. (2021). An Alternative to Antibiotics: Selected Methods to Combat Zoonotic Foodborne Bacterial Infections. Curr. Microbiol..

[42] Issenhuth-Jeanjean S., Roggentin P., Mikoleit M., Guibourdenche M., De Pinna E., Nair S., Fields P.I., Weill F.-X. (2014). Supplement 2008–2010 (no. 48) to the white–Kauffmann–Le minor scheme. Res. Microbiol..

[43] Cheng R.A., Eade C.R., Wiedmann M. (2019). Embracing Diversity: Differences in Virulence Mechanisms, Disease Severity, and Host Adaptations Contribute to the Success of Nontyphoidal Salmonella as a Foodborne Pathogen. Front. Microbiol..

[44] Townsend S.M., Kramer N.E., Edwards R., Baker S., Hamlin N., Simmonds M., Stevens K., Maloy S., Parkhill J., Dougan G. (2001). *Salmonella enterica* serovar Typhi possesses a unique repertoire of fimbrial gene sequences. Infect. Immun..

[45] Ferrari R.G., Rosario D.K., Cunha-Neto A., Mano S.B., Figueiredo E.E., Conte-Junior C.A. (2019). Worldwide epidemiology of Salmonella serovars in animal-based foods: A meta-analysis. Appl. Environ. Microbiol..

[46] Dróżdż M., Małaszczuk M., Paluch E., Pawlak A. (2021). Zoonotic potential and prevalence of Salmonella serovars isolated from pets. Infect. Ecol. Epidemiol..

[47] Rabsch W., Tschäpe H., Bäumler A.J. (2001). Non-typhoidal salmonellosis: Emerging problems. Microbes Infect..

[48] Branchu P., Bawn M., Kingsley R.A. (2018). Genome variation and molecular epidemiology of *Salmonella enterica* serovar Typhimurium pathovariants. Infect. Immun..

[49] Saraf S., Jafra B.S., Ray P., Rawat A., Verma S. (2021). Multidrug-Resistant Nontyphoidal Salmonella Associated with Invasive Disease in an Immunocompetent Child. Indian J. Pediatr..

[50] Porwollik S., Santiviago C., Cheng P., Florea L., McClelland M. (2005). Differences in gene content between *Salmonella enterica* serovar Enteritidis isolates and comparison to closely related serovars Gallinarum and Dublin. J. Bacteriol..

[51] Rabsch W., Andrews H.L., Kingsley R.A., Prager R., Tschäpe H., Adams L.G., Bäumler A.J. (2002). *Salmonella enterica* serotype Typhimurium and its host-adapted variants. Infect. Immun..

[52] Evangelopoulou G., Kritas S., Govaris A., Burriel A.R. (2013). Animal salmonelloses: A brief review of? host adaptation and host specificity? of *Salmonella* spp. Vet. World.

[53] Kingsley R.A., Bäumler A.J. (2000). Host adaptation and the emergence of infectious disease: The Salmonella paradigm. Mol. Microbiol..

[54] Langridge G.C., Fookes M., Connor T.R., Feltwell T., Feasey N., Parsons B.N., Seth-Smith H.M., Barquist L., Stedman A., Humphrey T. (2015). Patterns of genome evolution that have accompanied host adaptation in Salmonella. Proc. Natl. Acad. Sci. USA.

[55] Hansen-Wester I., Stecher B.r., Hensel M. (2002). Type III secretion of Salmonella enterica serovar Typhimurium translocated effectors and SseFG. Infect. Immun..

[56] Pucciarelli M.G., Garcia-del Portillo F. (2019). Salmonella intracellular lifestyles and their impact on host-to-host transmission. Microb. Transm..

[57] Gopinath S., Carden S., Monack D. (2012). Shedding light on Salmonella carriers. Trends Microbiol..

[58] Zhang H., Lou Z., Chen X., Cui Y., Wang H., Kou X., Ma C. (2019). Effect of simultaneous ultrasonic and microwave assisted hydrodistillation on the yield, composition, antibacterial and antibiofilm activity of essential oils from *Citrus medica* L. var. sarcodactylis. J. Food Eng..

[59] Foster N., Tang Y., Berchieri A., Geng S., Jiao X., Barrow P. (2021). Revisiting persistent Salmonella infection and the carrier state: What do we know?. Pathogens.

[60] Waldner L.L., MacKenzie K.D., Köster W., White A.P. (2012). From exit to entry: Long-term survival and transmission of Salmonella. Pathogens.

[61] Ahmer B.M., Gunn J.S. (2011). Interaction of Salmonella spp. with the intestinal microbiota. Front. Microbiol..

[62] Gonzalez-Escobedo G., Gunn J.S. (2013). Gallbladder epithelium as a niche for chronic Salmonella carriage. Infect. Immun..

[63] Dos Santos E.J.E., Azevedo R.P., Lopes A.T.S., Rocha J.M., Albuquerque G.R., Wenceslau A.A., Miranda F.R., Rodrigues D.D.P., Maciel B.M. (2020). Salmonella spp. in Wild Free-Living Birds from Atlantic Forest Fragments in Southern Bahia, Brazil. BioMed Res. Int..

[64] Marin C., Hernandiz A., Lainez M. (2009). Biofilm development capacity of Salmonella strains isolated in poultry risk factors and their resistance against disinfectants. Poult. Sci..

[65] Morton V.K., Kearney A., Coleman S., Viswanathan M., Chau K., Orr A., Hexemer A. (2019). Outbreaks of Salmonella illness associated with frozen raw breaded chicken products in Canada, 2015–2019. Epidemiol. Infect..

[66] Chanamé Pinedo L., Mughini-Gras L., Franz E., Hald T., Pires S.M. (2022). Sources and trends of human salmonellosis in Europe, 2015–2019: An analysis of outbreak data. Int. J. Food Microbiol..

[67] Laufer A.S., Grass J., Holt K., Whichard J.M., Griffin P.M., Gould L.H. (2015). Outbreaks of Salmonella infections attributed to beef —United States, 1973–2011. Epidemiol. Infect..

[68] Gutema F.D., Agga G.E., Abdi R.D., De Zutter L., Duchateau L., Gabriël S. (2019). Prevalence and Serotype Diversity of Salmonella in Apparently Healthy Cattle: Systematic Review and Meta-Analysis of Published Studies, 2000–2017. Front. Vet. Sci..

[69] Zając M., Wasyl D., Różycki M., Bilska-Zając E., Fafiński Z., Iwaniak W., Krajewska M., Hoszowski A., Konieczna O., Fafińska P. (2016). Free-living snakes as a source and possible vector of *Salmonella* spp. and parasites. Eur. J. Wildl. Res..

[70] Finley R., Ribble C., Aramini J., Vandermeer M., Popa M., Litman M., Reid-Smith R. (2007). The risk of salmonellae shedding by dogs fed Salmonella-contaminated commercial raw food diets. Can. Vet. J..

[71] Lefebvre S.L., Reid-Smith R., Boerlin P., Weese J.S. (2008). Evaluation of the risks of shedding Salmonellae and other potential pathogens by therapy dogs fed raw diets in Ontario and Alberta. Zoonoses Public Health.

[72] Younus M., Wilkins M.J., Davies H.D., Rahbar M.H., Funk J., Nguyen C., Siddiqi A.E., Cho S., Saeed M. (2010). Case-control study of disease determinants for non-typhoidal Salmonella infections among Michigan children. BMC Res. Notes.

[73] Cilia G., Turchi B., Fratini F., Bilei S., Bossù T., De Marchis M.L., Cerri D., Pacini M.I., Bertelloni F. (2021). Prevalence, Virulence and Antimicrobial Susceptibility of *Salmonella* spp., *Yersinia enterocolitica* and *Listeria monocytogenes* in European Wild Boar (Sus scrofa) Hunted in Tuscany (Central Italy). Pathogens.

[74] Navarro-Gonzalez N., Ugarte-Ruiz M., Domínguez L., Ruiz-Fons F., Jay-Russell M., Doyle M.P. (2016). A European Perspective on the Transmission of Foodborne Pathogens at the Wildlife–Livestock–Human Interface. Food Safety Risks from Wildlife: Challenges in Agriculture, Conservation, and Public Health.

[75] Gil Molino M., Garcia Sanchez A., Risco Perez D., Goncalves Blanco P., Quesada Molina A., Rey Pérez J., Martín Cano F.E., Cerrato Horrillo R., Hermoso-de-Mendoza Salcedo J., Fernandez Llario P. (2019). Prevalence of Salmonella spp. in tonsils, mandibular lymph nodes and faeces of wild boar from Spain and genetic relationship between isolates. Transbound. Emerg. Dis..

[76] Cummings K.J., Rodriguez-Rivera L.D., Grigar M.K., Rankin S.C., Mesenbrink B.T., Leland B.R., Bodenchuk M.J. (2016). Prevalence and Characterization of *Salmonella* Isolated from Feral Pigs Throughout Texas. Zoonoses Public Health.

[77] Farias L.F.P., Oliveira C.J.B., Medardus J.J., Molla B.Z., Wolfe B.A., Gebreyes W.A. (2015). Phenotypic and Genotypic Characterization of Salmonella enterica in Captive Wildlife and Exotic Animal Species in Ohio, USA. Zoonoses Public Health.

[78] Olsen A.R., Hammack T.S. (2000). Isolation of Salmonella spp. from the housefly, Musca domestica L., and the dump fly, Hydrotaea aenescens (Wiedemann) (Diptera: Muscidae), at caged-layer houses. J. Food Prot..

[79] Donoso A., Paredes N., Retamal P. (2020). Detection of Antimicrobial Resistant Salmonella enterica Strains in Larval and Adult Forms of Lesser Mealworm (Alphitobius diaperinus) From Industrial Poultry Farms. Front. Vet. Sci..

[80] Wang Y.C., Chang Y.C., Chuang H.L., Chiu C.C., Yeh K.S., Chang C.C., Hsuan S.L., Lin W.H., Chen T.H. (2011). Transmission of Salmonella between swine farms by the housefly (Musca domestica). J. Food Prot..

[81] Xu Y., Tao S., Hinkle N., Harrison M., Chen J. (2018). Salmonella, including antibiotic-resistant Salmonella, from flies captured from cattle farms in Georgia, U.S.A. Sci. Total Environ..

[82] Henzler D.J., Opitz H.M. (1992). The role of mice in the epizootiology of Salmonella enteritidis infection on chicken layer farms. Avian Dis..

[83] Lapuz R., Tani H., Sasai K., Shirota K., Katoh H., Baba E. (2008). The role of roof rats (*Rattus rattus*) in the spread of Salmonella Enteritidis and S. Infantis contamination in layer farms in eastern Japan. Epidemiol. Infect..

[84] Davies R.H., Wray C. (1995). Mice as carriers of Salmonella enteritidis on persistently infected poultry units. Vet. Rec..

[85] Meerburg B.G., Jacobs-Reitsma W.F., Wagenaar J.A., Kijlstra A. (2006). Presence of Salmonella and Campylobacter spp. in wild small mammals on organic farms. Appl. Environ. Microbiol..

[86] Pocock M.J., Searle J.B., Betts W.B., White P.C. (2001). Patterns of infection by Salmonella and Yersinia spp. in commensal house mouse (Mus musculus domesticus) populations. J. Appl. Microbiol..

[87] Le Moine V., Vannier P., Jestin A. (1987). Microbiological studies of wild rodents in farms as carriers of pig infectious agents. Prev. Vet. Med..

[88] Lee K.-H., Lee J.-Y., Roy P.K., Mizan M.F.R., Hossain M.I., Park S.H., Ha S.-D. (2020). Viability of Salmonella Typhimurium biofilms on major food-contact surfaces and eggshell treated during 35 days with and without water storage at room temperature. Poult. Sci..

[89] Elpers L., Kretzschmar J., Nuccio S.P., Bäumler A.J., Hensel M. (2020). Factors Required for Adhesion of Salmonella enterica Serovar Typhimurium to Corn Salad (*Valerianella locusta*). Appl. Environ. Microbiol..

[90] Liu H., Whitehouse C.A., Li B. (2018). Presence and Persistence of Salmonella in Water: The Impact on Microbial Quality of Water and Food Safety. Front. Public Health.

[91] Strawn L.K., Danyluk M.D., Worobo R.W., Wiedmann M. (2014). Distributions of Salmonella subtypes differ between two U.S. produce-growing regions. Appl. Environ. Microbiol..

[92] Bosilevac J.M., Guerini M.N., Kalchayanand N., Koohmaraie M. (2009). Prevalence and Characterization of Salmonellae in Commercial Ground Beef in the United States. Appl. Environ. Microbiol..

[93] Majowicz S.E., Musto J., Scallan E., Angulo F.J., Kirk M., O’Brien S.J., Jones T.F., Fazil A., Hoekstra R.M. (2010). The global burden of nontyphoidal Salmonella gastroenteritis. Clin. Infect. Dis..

[94] CDC (2023). Salmonella Outbreaks Associated with Not Ready-to-Eat Breaded, Stuffed Chicken Products—United States, 1998–2022. MMWR Morb. Mortal. Wkly. Rep. 2023.

[95] Griffith R.W., Carlson S.A., Krull A.C. (2019). Salmonellosis. Dis. Swine.

[96] Gantois I., Ducatelle R., Pasmans F., Haesebrouck F., Gast R., Humphrey T.J., Van Immerseel F. (2009). Mechanisms of egg contamination by Salmonella Enteritidis. FEMS Microbiol. Rev..

[97] Lee J.-S., Mogasale V.V., Mogasale V., Lee K. (2016). Geographical distribution of typhoid risk factors in low and middle income countries. BMC Infect. Dis..

[98] CDC (2011). Foodborne Diseases Active Surveillance Network, 2011 Surveillance Report.

[99] Center for Disease Control and Prevention (CDC) (2013). Incidence and trends of infection with pathogens transmitted commonly through food-foodborne diseases active surveillance network, 10 U.S. sites, 1996–2012. MMWR Morb. Mortal. Wkly. Rep..

[100] Jones T.F., Ingram L.A., Fullerton K.E., Marcus R., Anderson B.J., McCarthy P.V., Vugia D., Shiferaw B., Haubert N., Wedel S. (2006). A case-control study of the epidemiology of sporadic Salmonella infection in infants. Pediatrics.

[101] Bavishi C., Dupont H.L. (2011). Systematic review: The use of proton pump inhibitors and increased susceptibility to enteric infection. Aliment. Pharmacol. Ther..

[102] Nollet N., Maes D., De Zutter L., Duchateau L., Houf K., Huysmans K., Imberechts H., Geers R., de Kruif A., Van Hoof J. (2004). Risk factors for the herd-level bacteriologic prevalence of Salmonella in Belgian slaughter pigs. Prev. Vet. Med..

[103] Nielsen L.R., Schukken Y.H., Gröhn Y.T., Ersbøll A.K. (2004). Salmonella Dublin infection in dairy cattle: Risk factors for becoming a carrier. Prev. Vet. Med..

[104] Evans S., Davies R. (1996). Case control study of multiple-resistant Salmonella typhimurium DT104 infection of cattle in Great Britain. Vet. Rec..

[105] Kent E., Okafor C., Caldwell M., Walker T., Whitlock B., Lear A. (2021). Control of Salmonella Dublin in a bovine dairy herd. J. Vet. Intern. Med..

[106] Ethèves M.A., Choisis N., Alvarez S., Dalleau F., Hascoat J., Gallard V., Cardinale E. (2021). Risk factors for Salmonella enterica subsp. enterica persistence in broiler-chicken flocks on Reunion Island. Heliyon.

[107] Crump J.A., Braden C.R., Dey M.E., Hoekstra R.M., Rickelman-Apisa J.M., Baldwin D.A., De Fijter S.J., Nowicki S.F., Koch E.M., Bannerman T.L. (2003). Outbreaks of Escherichia coli O157 infections at multiple county agricultural fairs: A hazard of mixing cattle, concession stands and children. Epidemiol. Infect..

[108] Cardoso M.J., Nicolau A.I., Borda D., Nielsen L., Maia R.L., Møretrø T., Ferreira V., Knøchel S., Langsrud S., Teixeira P. (2021). Salmonella in eggs: From shopping to consumption-A review providing an evidence-based analysis of risk factors. Compr. Rev. Food Sci. Food Saf..

[109] Liu Y., Jiang J., Ed-Dra A., Li X., Peng X., Xia L., Guo Q., Yao G., Yue M. (2021). Prevalence and genomic investigation of Salmonella isolates recovered from animal food-chain in Xinjiang, China. Food Res. Int..

[110] Reddy E.A., Shaw A.V., Crump J.A. (2010). Community-acquired bloodstream infections in Africa: A systematic review and meta-analysis. Lancet Infect. Dis..

[111] Thakur R., Suri C.R., Rishi P. (2022). Contribution of typhoid toxin in the pathogenesis of Salmonella Typhi. Microb. Pathog..

[112] Patel T.A., Armstrong M., Morris-Jones S.D., Wright S.G., Doherty T. (2010). Imported enteric fever: Case series from the hospital for tropical diseases, London, United Kingdom. Am. J. Trop. Med. Hyg..

[113] Kuvandik C., Karaoglan I., Namiduru M., Baydar I. (2009). Predictive value of clinical and laboratory findings in the diagnosis of the enteric fever. New Microbiol..

[114] Galán J.E. (2016). Typhoid toxin provides a window into typhoid fever and the biology of Salmonella Typhi. Proc. Natl. Acad. Sci. USA.

[115] Crump J.A. (2019). Progress in Typhoid Fever Epidemiology. Clin. Infect. Dis..

[116] Parry C.M., Hien T.T., Dougan G., White N.J., Farrar J.J. (2002). Typhoid fever. N. Engl. J. Med..

[117] Allen J.C., Toapanta F.R., Baliban S.M., Sztein M.B., Tennant S.M. (2023). Reduced immunogenicity of a live Salmonella enterica serovar Typhimurium vaccine in aged mice. Front. Immunol..

[118] CDC National Enteric Disease Surveillance: Salmonella Annual Report. https://www.cdc.gov/nationalsurveillance/salmonella-surveillance.html.

[119] Bakhshandeh B., Sorboni S.G., Haghighi D.M., Ahmadi F., Dehghani Z., Badiei A. (2022). New analytical methods using carbon-based nanomaterials for detection of Salmonella species as a major food poisoning organism in water and soil resources. Chemosphere.

[120] Turgeon P., Ng V., Murray R., Nesbitt A. (2018). Forecasting the incidence of salmonellosis in seniors in Canada: A trend analysis and the potential impact of the demographic shift. PLoS ONE.

[121] Pulford C.V., Perez-Sepulveda B.M., Canals R., Bevington J.A., Bengtsson R.J., Wenner N., Rodwell E.V., Kumwenda B., Zhu X., Bennett R.J. (2021). Stepwise evolution of Salmonella Typhimurium ST313 causing bloodstream infection in Africa. Nat. Microbiol..

[122] Acheson D., Hohmann E.L. (2001). Nontyphoidal Salmonellosis. Clin. Infect. Dis..

[123] Scallan E., Hoekstra R.M., Angulo F.J., Tauxe R.V., Widdowson M.A., Roy S.L., Jones J.L., Griffin P.M. (2011). Foodborne illness acquired in the United States—Major pathogens. Emerg. Infect. Dis..

[124] Ajene A.N., Fischer Walker C.L., Black R.E. (2013). Enteric pathogens and reactive arthritis: A systematic review of Campylobacter, salmonella and Shigella-associated reactive arthritis. J. Health Popul. Nutr..

[125] Ehuwa O., Jaiswal A.K., Jaiswal S. (2021). Salmonella, Food Safety and Food Handling Practices. Foods.

[126] Schempp C.M., Schauer F., Huhn C.K., Venhoff N., Finzel S. (2019). Skin inflammation associated with arthritis, synovitis and enthesitis. Part 2: Rheumatoid arthritis, reactive arthritis, Reiter’s syndrome, Lyme borreliosis, dermatomyositis and lupus erythematosus. J. Dtsch. Dermatol. Ges..

[127] Tsuchiya Y., Loza E., Villa-Gomez G., Trujillo C.C., Baez S., Asai T., Ikoma T., Endoh K., Nakamura K. (2018). Metagenomics of microbial communities in gallbladder bile from patients with gallbladder cancer or cholelithiasis. Asian Pac. J. Cancer Prev..

[128] Zha L., Garrett S., Sun J. (2019). Salmonella infection in chronic inflammation and gastrointestinal cancer. Diseases.

[129] DuPont H.L. (2009). Bacterial diarrhea. N. Engl. J. Med..

[130] Serpil Kahya D., Maria Teresa M. (2017). Salmonellosis in Animals. Salmonella—A Re-emerging Pathogen.

[131] Barrow P.A., Jones M.A., Smith A.L., Wigley P. (2012). The long view: Salmonella—The last forty years. Avian Pathol..

[132] Zhou X., Kang X., Zhou K., Yue M. (2022). A global dataset for prevalence of *Salmonella Gallinarum* between 1945 and 2021. Sci. Data.

[133] Shen X., Zhang A., Gu J., Zhao R., Pan X., Dai Y., Yin L., Zhang Q., Hu X., Wang H. (2022). Evaluating Salmonella pullorum dissemination and shedding patterns and antibody production in infected chickens. BMC Vet. Res..

[134] Uzzau S., Leori G.S., Petruzzi V., Watson P.R., Schianchi G., Bacciu D., Mazzarello V., Wallis T.S., Rubino S. (2001). Salmonella enterica serovar-host specificity does not correlate with the magnitude of intestinal invasion in sheep. Infect. Immun..

[135] Habrun B., Listes E., Spicic S., Cvetnic Z., Lukacevic D., Jemersic L., Lojkic M., Kompes G. (2006). An outbreak of Salmonella Abortusovis abortions in sheep in south Croatia. J. Vet. Med. B Infect. Dis. Vet. Public Health.

[136] Vanselow B.A., Hum S., Hornitzky M.A., Eamens G.J., Quinn K. (2007). Salmonella Typhimurium persistence in a Hunter Valley dairy herd. Aust. Vet. J..

[137] Askari N., Mashhad Rafiee S., Amini K. (2020). A case control study of Salmonella SPP. infection in stray dogs in Tehran shelters and the correlation between paraclinical tests results and clinical findings. Arch. Razi Inst..

[138] Feary D.J., Hassel D.M. (2006). Enteritis and colitis in horses. Vet. Clin. North Am. Equine Pract..

[139] Burgess B.A., Noyes N.R., Bolte D.S., Hyatt D.R., van Metre D.C., Morley P.S. (2015). Rapid Salmonella detection in experimentally inoculated equine faecal and veterinary hospital environmental samples using commercially available lateral flow immunoassays. Equine Vet. J..

[140] CDC (2011). Vital signs: Incidence and trends of infection with pathogens transmitted commonly through food—Foodborne diseases active surveillance network, 10 U.S. sites, 1996–2010. MMWR Morb. Mortal. Wkly. Rep..

[141] Jackson B.R., Griffin P.M., Cole D., Walsh K.A., Chai S.J. (2013). Outbreak-associated Salmonella enterica serotypes and food Commodities, United States, 1998–2008. Emerg. Infect. Dis..

[142] Soto-Dávila M., Hossain A., Chakraborty S., Rise M.L., Santander J. (2019). Aeromonas salmonicida subsp. salmonicida Early Infection and Immune Response of Atlantic Cod (*Gadus morhua* L.) Primary Macrophages. Front. Immunol..

[143] Younes M., Aquilina G., Engel K.-H., Fowler P., Frutos Fernandez M.J., Fürst P., Gürtler R., Gundert-Remy U., Husøy T., EFSA Panel on Food Additives and Flavourings (FAF) (2019). Safety of use of Monk fruit extract as a food additive in different food categories. EFSA J..

[144] Thomas M.K., Murray R., Flockhart L., Pintar K., Pollari F., Fazil A., Nesbitt A., Marshall B. (2013). Estimates of the burden of foodborne illness in Canada for 30 specified pathogens and unspecified agents, circa 2006. Foodborne Pathog. Dis..

[145] Government of Canada (2015). National Enteric Surveillance Program (NESP) Annual Summary 2013.

[146] Hendriksen R.S., Vieira A.R., Karlsmose S., Lo Fo Wong D.M., Jensen A.B., Wegener H.C., Aarestrup F.M. (2011). Global monitoring of Salmonella serovar distribution from the World Health Organization Global Foodborne Infections Network Country Data Bank: Results of quality assured laboratories from 2001 to 2007. Foodborne Pathog. Dis..

[147] Hassan R., Buuck S., Noveroske D. (2019). Multistate outbreak of Salmonella infections linked to raw turkey products—United States, 2017–2019. MMWR Morb. Mortal. Wkly. Rep..

[148] EFSA The European Union Summary Report on Trends and Sources of Zoonoses, Zoonotic Agents and Food-Borne Outbreaks in 2017. https://efsa.onlinelibrary.wiley.com/doi/10.2903/j.efsa.2018.5500..

[149] European Centre for Disease P., Control E.F.S.A. (2022). Multi-country outbreak of monophasic Salmonella Typhimurium sequence type 34 linked to chocolate products–first update–18 May 2022. EFSA Support. Publ..

[150] Cevallos-Cevallos J.M., Gu G., Danyluk M.D., van Bruggen A.H. (2012). Adhesion and splash dispersal of Salmonella enterica Typhimurium on tomato leaflets: Effects of rdar morphotype and trichome density. Int. J. Food Microbiol..

[151] Ibarra J.A., Steele-Mortimer O. (2009). Salmonella—The ultimate insider. Salmonella virulence factors that modulate intracellular survival. Cell. Microbiol..

[152] Kumar H., Kawai T., Akira S. (2009). Pathogen recognition in the innate immune response. Biochem. J..

[153] Kage H., Takaya A., Ohya M., Yamamoto T. (2008). Coordinated regulation of expression of Salmonella pathogenicity island 1 and flagellar type III secretion systems by ATP-dependent ClpXP protease. J. Bacteriol..

[154] Lang T., Mansell A. (2007). The negative regulation of Toll-like receptor and associated pathways. Immunol. Cell Biol..

[155] Khan S.A., Everest P., Servos S., Foxwell N., Zähringer U., Brade H., Rietschel E.T., Dougan G., Charles I.G., Maskell D.J. (1998). A lethal role for lipid A in Salmonella infections. Mol. Microbiol..

[156] Raffatellu M., Chessa D., Wilson R.P., Dusold R., Rubino S., Bäumler A.J. (2005). The Vi capsular antigen of Salmonella enterica serotype Typhi reduces Toll-like receptor-dependent interleukin-8 expression in the intestinal mucosa. Infect. Immun..

[157] Bignold L.P., Rogers S.D., Siaw T.M., Bahnisch J. (1991). Inhibition of chemotaxis of neutrophil leukocytes to interleukin-8 by endotoxins of various bacteria. Infect. Immun..

[158] Jessen D.L., Osei-Owusu P., Toosky M., Roughead W., Bradley D.S., Nilles M.L. (2014). Type III secretion needle proteins induce cell signaling and cytokine secretion via Toll-like receptors. Infect. Immun..

[159] Mambu J., Virlogeux-Payant I., Holbert S., Grépinet O., Velge P., Wiedemann A. (2017). An Updated View on the Rck Invasin of Salmonella: Still Much to Discover. Front. Cell. Infect. Microbiol..

[160] Mellor K.C., Blackwell G.A., Cawthraw S.A., Mensah N.E., Reid S.W.J., Thomson N.R., Petrovska L., Mather A.E. (2022). Contrasting long-term dynamics of antimicrobial resistance and virulence plasmids in *Salmonella typhimurium* from animals. Microb. Genom..

[161] Zuo L., Zhou L., Wu C., Wang Y., Li Y., Huang R., Wu S. (2020). Salmonella spvC Gene Inhibits Pyroptosis and Intestinal Inflammation to Aggravate Systemic Infection in Mice. Front. Microbiol..

[162] Rodríguez-Beltrán J., DelaFuente J., León-Sampedro R., MacLean R.C., San Millán Á. (2021). Beyond horizontal gene transfer: The role of plasmids in bacterial evolution. Nat. Rev. Microbiol..

[163] Sengupta M., Austin S. (2011). Prevalence and significance of plasmid maintenance functions in the virulence plasmids of pathogenic bacteria. Infect. Immun..

[164] Kosarewicz A., Königsmaier L., Marlovits T.C. (2012). The blueprint of the type-3 injectisome. Philos. Trans. R. Soc. Lond B Biol. Sci..

[165] Srikanth C.V., Mercado-Lubo R., Hallstrom K., McCormick B.A. (2011). Salmonella effector proteins and host-cell responses. Cell. Mol. Life Sci..

[166] LaRock D.L., Chaudhary A., Miller S.I. (2015). Salmonellae interactions with host processes. Nat. Rev. Microbiol..

[167] Figueira R., Holden D.W. (2012). Functions of the Salmonella pathogenicity island 2 (SPI-2) type III secretion system effectors. Microbiology.

[168] Sellin M.E., Müller A.A., Felmy B., Dolowschiak T., Diard M., Tardivel A., Maslowski K.M., Hardt W.D. (2014). Epithelium-intrinsic NAIP/NLRC4 inflammasome drives infected enterocyte expulsion to restrict Salmonella replication in the intestinal mucosa. Cell Host Microbe.

[169] McGhie E.J., Brawn L.C., Hume P.J., Humphreys D., Koronakis V. (2009). Salmonella takes control: Effector-driven manipulation of the host. Curr. Opin. Microbiol..

[170] Lenders M.H.H., Weidtkamp-Peters S., Kleinschrodt D., Jaeger K.-E., Smits S.H.J., Schmitt L. (2015). Directionality of substrate translocation of the hemolysin A Type I secretion system. Sci. Rep..

[171] Li X., Bleumink-Pluym N.M.C., Luijkx Y.M.C.A., Wubbolts R.W., van Putten J.P.M., Strijbis K. (2019). MUC1 is a receptor for the Salmonella SiiE adhesin that enables apical invasion into enterocytes. PLOS Pathog..

[172] Main-Hester K.L., Colpitts K.M., Thomas G.A., Fang F.C., Libby S.J. (2008). Coordinate regulation of Salmonella pathogenicity island 1 (SPI1) and SPI4 in Salmonella enterica serovar Typhimurium. Infect. Immun..

[173] Fang L., Shen H., Tang Y., Fang W. (2015). Superoxide dismutase of Streptococcus suis serotype 2 plays a role in anti-autophagic response by scavenging reactive oxygen species in infected macrophages. Vet. Microbiol..

[174] Felmy B., Songhet P., Slack E.M., Müller A.J., Kremer M., Van Maele L., Cayet D., Heikenwalder M., Sirard J.C., Hardt W.D. (2013). NADPH oxidase deficient mice develop colitis and bacteremia upon infection with normally avirulent, TTSS-1- and TTSS-2-deficient *Salmonella typhimurium*. PLoS ONE.

[175] Tang B., Elbediwi M., Nambiar R.B., Yang H., Lin J., Yue M. (2022). Genomic Characterization of Antimicrobial-Resistant Salmonella enterica in Duck, Chicken, and Pig Farms and Retail Markets in Eastern China. Microbiol. Spectr..

[176] Wang F., Deng L., Huang F., Wang Z., Lu Q., Xu C. (2020). Flagellar Motility Is Critical for Salmonella enterica Serovar Typhimurium Biofilm Development. Front. Microbiol..

[177] Krishnakumar R., Kim B., Mollo E.A., Imlay J.A., Slauch J.M. (2007). Structural properties of periplasmic SodCI that correlate with virulence in Salmonella enterica serovar Typhimurium. J. Bacteriol..

[178] Rehman T., Yin L., Latif M.B., Chen J., Wang K., Geng Y., Huang X., Abaidullah M., Guo H., Ouyang P. (2019). Adhesive mechanism of different Salmonella fimbrial adhesins. Microb. Pathog..

[179] Kolenda R., Ugorski M., Grzymajlo K. (2019). Everything You Always Wanted to Know About Salmonella Type 1 Fimbriae, but Were Afraid to Ask. Front. Microbiol..

[180] Grzymajło K., Ugorski M., Kolenda R., Kędzierska A., Kuźmińska-Bajor M., Wieliczko A. (2013). FimH adhesin from host unrestricted Salmonella Enteritidis binds to different glycoprotein ligands expressed by enterocytes from sheep, pig and cattle than FimH adhesins from host restricted Salmonella Abortus-ovis, Salmonella Choleraesuis and Salmonella Dublin. Vet. Microbiol..

[181] Uchiya K.I., Kamimura Y., Jusakon A., Nikai T. (2019). Salmonella Fimbrial Protein FimH Is Involved in Expression of Proinflammatory Cytokines in a Toll-Like Receptor 4-Dependent Manner. Infect. Immun..

[182] Sano G., Takada Y., Goto S., Maruyama K., Shindo Y., Oka K., Matsui H., Matsuo K. (2007). Flagella facilitate escape of Salmonella from oncotic macrophages. J. Bacteriol..

[183] Wang G., Song Q., Huang S., Wang Y., Cai S., Yu H., Ding X., Zeng X., Zhang J. (2020). Effect of antimicrobial peptide microcin J25 on growth performance, immune regulation, and intestinal microbiota in broiler chickens challenged with *Escherichia coli* and Salmonella. Animals.

[184] Sokaribo A.S., Hansen E.G., McCarthy M., Desin T.S., Waldner L.L., MacKenzie K.D., Mutwiri G., Herman N.J., Herman D.J., Wang Y. (2020). Metabolic Activation of CsgD in the Regulation of Salmonella Biofilms. Microorganisms.

[185] Haiko J., Westerlund-Wikström B. (2013). The role of the bacterial flagellum in adhesion and virulence. Biology.

[186] Horstmann J.A., Zschieschang E., Truschel T., de Diego J., Lunelli M., Rohde M., May T., Strowig T., Stradal T., Kolbe M. (2017). Flagellin phase-dependent swimming on epithelial cell surfaces contributes to productive Salmonella gut colonisation. Cell. Microbiol..

[187] Liston S.D., Ovchinnikova O.G., Whitfield C. (2016). Unique lipid anchor attaches Vi antigen capsule to the surface of Salmonella enterica serovar Typhi. Proc. Natl. Acad. Sci. USA.

[188] Hart P.J., O’Shaughnessy C.M., Siggins M.K., Bobat S., Kingsley R.A., Goulding D.A., Crump J.A., Reyburn H., Micoli F., Dougan G. (2016). Differential Killing of *Salmonella enterica* Serovar Typhi by Antibodies Targeting Vi and Lipopolysaccharide O:9 Antigen. PLoS ONE.

[189] Hiyoshi H., Tiffany C.R., Bronner D.N., Bäumler A.J. (2018). Typhoidal Salmonella serovars: Ecological opportunity and the evolution of a new pathovar. FEMS Microbiol. Rev..

[190] Chong A., Lee S., Yang Y.A., Song J. (2017). The Role of Typhoid Toxin in Salmonella Typhi Virulence. Yale J. Biol. Med..

[191] Needham B.D., Trent M.S. (2013). Fortifying the barrier: The impact of lipid A remodelling on bacterial pathogenesis. Nat. Rev. Microbiol..

[192] Richards S.M., Strandberg K.L., Gunn J.S. (2010). Salmonella-regulated lipopolysaccharide modifications. Subcell. Biochem..

[193] Kong Q., Yang J., Liu Q., Alamuri P., Roland K.L., Curtiss R. (2011). Effect of deletion of genes involved in lipopolysaccharide core and O-antigen synthesis on virulence and immunogenicity of Salmonella enterica serovar typhimurium. Infect. Immun..

[194] Chen C.-Y., Tsen H.-Y., Lin C.-L., Yu B., Chen C.-S. (2012). Oral administration of a combination of select lactic acid bacteria strains to reduce the Salmonella invasion and inflammation of broiler chicks. Poult. Sci..

[195] Römling U. (2005). Characterization of the rdar morphotype, a multicellular behaviour in Enterobacteriaceae. Cell. Mol. Life Sci..

[196] Collinson S.K., Clouthier S.C., Doran J.L., Banser P.A., Kay W.W. (1996). Salmonella enteritidis agfBAC operon encoding thin, aggregative fimbriae. J. Bacteriol..

[197] Zakikhany K., Harrington C.R., Nimtz M., Hinton J.C., Römling U. (2010). Unphosphorylated CsgD controls biofilm formation in Salmonella enterica serovar Typhimurium. Mol. Microbiol..

[198] Kader A., Simm R., Gerstel U., Morr M., Römling U. (2006). Hierarchical involvement of various GGDEF domain proteins in rdar morphotype development of Salmonella enterica serovar Typhimurium. Mol. Microbiol..

[199] Van Immerseel F., Russell J.B., Flythe M.D., Gantois I., Timbermont L., Pasmans F., Haesebrouck F., Ducatelle R. (2006). The use of organic acids to combat Salmonella in poultry: A mechanistic explanation of the efficacy. Avian Pathol..

[200] Galipó E., Zoche-Golob V., Sassu E.L., Prigge C., Sjölund M., Tobias T., Rzeżutka A., Smith R.P., Burow E. (2023). Prioritization of pig farm biosecurity for control of Salmonella and hepatitis E virus infections: Results of a European expert opinion elicitation. Porc. Health Manag..

[201] Madec F. Good Practices for Biosecurity in the Pig Sector: Issues and Options in Developing and Transition Countries. https://www.fao.org/3/i1435e/i1435e00.htm.

[202] Sharma B. (2010). Poultry Production, Management and Bio-Security Measures. J. Agric. Environ..

[203] Jensen A.N., Dalsgaard A., Stockmarr A., Nielsen E.M., Baggesen D.L. (2006). Survival and transmission of Salmonella enterica serovar typhimurium in an outdoor organic pig farming environment. Appl. Environ. Microbiol..

[204] Mannion C., Leonard F.C., Lynch P.B., Egan J. (2007). Efficacy of cleaning and disinfection on pig farms in Ireland. Vet. Rec..

[205] Trampel D.W., Holder T.G., Gast R.K. (2014). Integrated farm management to prevent *Salmonella Enteritidis* contamination of eggs. J. Appl. Poult. Res..

[206] Lestari V.S., Sirajuddin S.N., Kasim K. (2011). Adoption of biosecurity measures by layer smallholders. J. Indones. Trop. Anim. Agric..

[207] Mee J.F., Geraghty T., O’Neill R., More S.J. (2012). Bioexclusion of diseases from dairy and beef farms: Risks of introducing infectious agents and risk reduction strategies. Vet. J..

[208] Renault V., Damiaans B., Sarrazin S., Humblet M.F., Dewulf J., Saegerman C. (2018). Biosecurity practices in Belgian cattle farming: Level of implementation, constraints and weaknesses. Transbound. Emerg. Dis..

[209] Tilli G., Laconi A., Galuppo F., Mughini-Gras L., Piccirillo A. (2022). Assessing Biosecurity Compliance in Poultry Farms: A Survey in a Densely Populated Poultry Area in North East Italy. Animals.

[210] Busani L., Dalla Pozza M., Bonfanti L., Toson M., Ferrè N., Marangon S. (2007). Intervention strategies for low-pathogenic avian influenza control in Italy. Avian Dis..

[211] Gelaude P., Schlepers M., Verlinden M., Laanen M., Dewulf J. (2014). Biocheck.UGent: A quantitative tool to measure biosecurity at broiler farms and the relationship with technical performances and antimicrobial use. Poult. Sci..

[212] Van Limbergen T., Dewulf J., Klinkenberg M., Ducatelle R., Gelaude P., Méndez J., Heinola K., Papasolomontos S., Szeleszczuk P., Maes D. (2018). Scoring biosecurity in European conventional broiler production. Poult. Sci..

[213] Maunsell F., Donovan G.A. (2008). Biosecurity and risk management for dairy replacements. Vet. Clin. North Am. Food Anim. Pract..

[214] Monitoring N.A.H. (2004). Dairy 2002:Animal Disease Exclusion Practices on U.S. Dairy Operations.

[215] Verwoerd D.W. (2015). Definition of a vector and a vector-borne disease. Rev. Sci. Tech..

[216] Spalding M.G. (2009). Diseases of Poultry.

[217] Meerburg B.G., Kijlstra A. (2007). Role of rodents in transmission of Salmonella and Campylobacter. J. Sci. Food Agric..

[218] Rebeca Z.-S., Andrea Molina A., Mihai M. (2017). Preharvest Salmonella Risk Contamination and the Control Strategies. Current Topics in Salmonella and Salmonellosis.

[219] Axtell R.C. (1986). Fly Management in Poultry Production: Cultural, Biological, and Chemical1. Poult. Sci..

[220] Balaraman V., Drolet B.S., Mitzel D.N., Wilson W.C., Owens J., Gaudreault N.N., Meekins D.A., Bold D., Trujillo J.D., Noronha L.E. (2021). Mechanical transmission of SARS-CoV-2 by house flies. Parasites Vectors.

[221] Azizi-Lalabadi M., Rahimzadeh-Sani Z., Feng J., Hosseini H., Jafari S.M. (2023). The impact of essential oils on the qualitative properties, release profile, and stimuli-responsiveness of active food packaging nanocomposites. Crit. Rev. Food Sci. Nutr..

[222] Ong S.Q., Ab Majid A.H., Ahmad H. (2017). Insecticide Residues on Poultry Manures: Field Efficacy Test on Selected Insecticides in Managing Musca Domestica Population. Trop. Life Sci. Res..

[223] Isman M.B. (2020). Bioinsecticides based on plant essential oils: A short overview. Z. Naturforsch. C J. Biosci..

[224] Weese J.S. (2004). Barrier precautions, isolation protocols, and personal hygiene in veterinary hospitals. Vet. Clin. N. Am. Equine Pract..

[225] Wierup M. (2000). The control of microbial diseases in animals: Alternatives to the use of antibiotics. Int. J. Antimicrob. Agents.

[226] Martelli F., Lambert M., Butt P., Cheney T., Tatone F.A., Callaby R., Rabie A., Gosling R.J., Fordon S., Crocker G. (2017). Evaluation of an enhanced cleaning and disinfection protocol in Salmonella contaminated pig holdings in the United Kingdom. PLoS ONE.

[227] Food and Agriculture Organization (FAO) (2015). Interventions for the Control of Non-Typhoidal Salmonella spp. in Beef and Pork.

[228] Braden C.R. (2006). Salmonella enterica serotype Enteritidis and eggs: A national epidemic in the United States. Clin. Infect. Dis..

[229] Jørgensen F., Bailey R., Williams S., Henderson P., Wareing D.R., Bolton F.J., Frost J.A., Ward L., Humphrey T.J. (2002). Prevalence and numbers of *Salmonella* and *Campylobacter* spp. on raw, whole chickens in relation to sampling methods. Int. J. Food Microbiol..

[230] Bhandari M., Poelstra J.W., Kauffman M., Varghese B., Helmy Y.A., Scaria J., Rajashekara G. (2023). Genomic Diversity, Antimicrobial Resistance, Plasmidome, and Virulence Profiles of *Salmonella* Isolated from Small Specialty Crop Farms Revealed by Whole-Genome Sequencing. Antibiotics.

[231] Wright G.D. (2010). Antibiotic resistance in the environment: A link to the clinic?. Curr. Opin. Microbiol..

[232] Liljebjelke K.A., Hofacre C.L., White D.G., Ayers S., Lee M.D., Maurer J.J. (2017). Diversity of antimicrobial resistance phenotypes in Salmonella isolated from commercial poultry farms. Front. Vet. Sci..

[233] Sneeringer S., MacDonald J., Key N., McBride W. (2015). Economics of Antibiotic Use in US. Livestock Production.

[234] Levy S.B., Marshall B. (2004). Antibacterial resistance worldwide: Causes, challenges and responses. Nat. Med..

[235] Gebreyes W.A., Thakur S. (2005). Multidrug-resistant *Salmonella enterica* serovar Muenchen from pigs and humans and potential interserovar transfer of antimicrobial resistance. Antimicrob. Agents Chemother..

[236] Elsayed M.M., El-Basrey Y.F.H., El-Baz A.H., Dowidar H.A., Shami A., Al-Saeed F.A., Alsamghan A., Salem H.M., Alhazmi W.A., El-Tarabily K.A. (2023). Ecological incidence, genetic diversity, multidrug resistance of Salmonella enteritidis recovered from broiler and layer chicken farms. Poult. Sci..

[237] Srednik M.E., Morningstar-Shaw B.R., Hicks J.A., Tong C., Mackie T.A., Schlater L.K. (2023). Whole-genome sequencing and phylogenetic analysis capture the emergence of a multi-drug resistant Salmonella enterica serovar Infantis clone from diagnostic animal samples in the United States. Front. Microbiol..

[238] Leon I.M., Lawhon S.D., Norman K.N., Threadgill D.S., Ohta N., Vinasco J., Scott H.M. (2018). Serotype Diversity and Antimicrobial Resistance among Salmonella enterica Isolates from Patients at an Equine Referral Hospital. Appl. Environ. Microbiol..

[239] Gebreyes W.A., Thakur S., Morrow W.M. (2006). Comparison of prevalence, antimicrobial resistance, and occurrence of multidrug-resistant Salmonella in antimicrobial-free and conventional pig production. J. Food Prot..

[240] Lynne A.M., Dorsey L.L., David D.E., Foley S.L. (2009). Characterisation of antibiotic resistance in host-adapted Salmonella enterica. Int. J. Antimicrob. Agents.

[241] Alagawany M., Abd El-Hack M.E., Farag M.R., Sachan S., Karthik K., Dhama K. (2018). The use of probiotics as eco-friendly alternatives for antibiotics in poultry nutrition. Environ. Sci. Pollut. Res..

[242] Helmy Y.A., Taha-Abdelaziz K., Hawwas H.A.E., Ghosh S., AlKafaas S.S., Moawad M.M.M., Saied E.M., Kassem I.I., Mawad A.M.M. (2023). Antimicrobial Resistance and Recent Alternatives to Antibiotics for the Control of Bacterial Pathogens with an Emphasis on Foodborne Pathogens. Antibiotics.

[243] de Melo Pereira G.V., de Oliveira Coelho B., Júnior A.I.M., Thomaz-Soccol V., Soccol C.R. (2018). How to select a probiotic? A review and update of methods and criteria. Biotechnol. Adv..

[244] Hill C., Guarner F., Reid G., Gibson G.R., Merenstein D.J., Pot B., Morelli L., Canani R.B., Flint H.J., Salminen S. (2014). The International Scientific Association for Probiotics and Prebiotics consensus statement on the scope and appropriate use of the term probiotic. Nat. Rev. Gastroenterol. Hepatol..

[245] Kulkarni R.R., Gaghan C., Gorrell K., Sharif S., Taha-Abdelaziz K. (2022). Probiotics as alternatives to antibiotics for the prevention and control of necrotic enteritis in chickens. Pathogens.

[246] Alizadeh M., Bavananthasivam J., Shojadoost B., Astill J., Taha-Abdelaziz K., Alqazlan N., Boodhoo N., Shoja Doost J., Sharif S. (2021). In ovo and oral administration of probiotic lactobacilli modulate cell-and antibody-mediated immune responses in newly hatched chicks. Front. Immunol..

[247] Dawood M.A., Koshio S., Esteban M.Á. (2018). Beneficial roles of feed additives as immunostimulants in aquaculture: A review. Rev. Aquac..

[248] Tuomola E.M., Ouwehand A.C., Salminen S.J. (1999). The effect of probiotic bacteria on the adhesion of pathogens to human intestinal mucus. FEMS Immunol. Med. Microbiol..

[249] Liu D., Jiang X.-Y., Zhou L.-S., Song J.-H., Zhang X. (2016). Effects of probiotics on intestinal mucosa barrier in patients with colorectal cancer after operation: Meta-analysis of randomized controlled trials. Medicine.

[250] Stavropoulou E., Bezirtzoglou E. (2020). Probiotics in Medicine: A Long Debate. Front. Immunol..

[251] Timmerman H.M., Koning C.J., Mulder L., Rombouts F.M., Beynen A.C. (2004). Monostrain, multistrain and multispecies probiotics—A comparison of functionality and efficacy. Int. J. Food Microbiol..

[252] Wang Y.-C., Hu S.-Y., Chiu C.-S., Liu C.-H. (2019). Multiple-strain probiotics appear to be more effective in improving the growth performance and health status of white shrimp, Litopenaeus vannamei, than single probiotic strains. Fish Shellfish Immunol..

[253] Kathayat D., Closs G., Helmy Y.A., Deblais L., Srivastava V., Rajashekara G. (2022). In Vitro and In Vivo Evaluation of Lacticaseibacillus rhamnosus GG and Bifidobacterium lactis Bb12 Against Avian Pathogenic Escherichia coli and Identification of Novel Probiotic-Derived Bioactive Peptides. Probiotics Antimicrob. Proteins.

[254] Ouwehand A.C., Invernici M.M., Furlaneto F.A., Messora M.R. (2018). Effectiveness of multi-strain versus single-strain probiotics: Current status and recommendations for the future. J. Clin. Gastroenterol..

[255] Puvanasundram P., Chong C.M., Sabri S., Yusoff M.S.M., Lim K.C., Karim M. (2022). Efficacy of Single and Multi-Strain Probiotics on In Vitro Strain Compatibility, Pathogen Inhibition, Biofilm Formation Capability, and Stress Tolerance. Biology.

[256] Mountzouris K., Balaskas C., Xanthakos I., Tzivinikou A., Fegeros K. (2009). Effects of a multi-species probiotic on biomarkers of competitive exclusion efficacy in broilers challenged with Salmonella enteritidis. Br. Poult. Sci..

[257] Thirabunyanon M., Thongwittaya N. (2012). Protection activity of a novel probiotic strain of Bacillus subtilis against Salmonella Enteritidis infection. Res. Vet. Sci..

[258] Chang C.H., Teng P.Y., Lee T.T., Yu B. (2020). Effects of multi-strain probiotic supplementation on intestinal microbiota, tight junctions, and inflammation in young broiler chickens challenged with Salmonella enterica subsp. enterica. Asian-Australas. J. Anim. Sci..

[259] Acharjee M., Hasan F., Islam T., Nur I.T., Begum N., Mazumder C., Lubna M.A., Zerin N., Shahriar A., Mahmud M.R. (2022). In-vitro antibacterial activity of commercially available probiotics on food-borne pathogens along with their synergistic effects with synthetic drugs. Metab. Open.

[260] Bhogoju S., Nahashon S., Wang X., Darris C., Kilonzo-Nthenge A. (2018). A comparative analysis of microbial profile of Guinea fowl and chicken using metagenomic approach. PLoS ONE.

[261] El-Sharkawy H., Tahoun A., Rizk A.M., Suzuki T., Elmonir W., Nassef E., Shukry M., Germoush M.O., Farrag F., Bin-Jumah M. (2020). Evaluation of Bifidobacteria and Lactobacillus Probiotics as Alternative Therapy for Salmonella typhimurium Infection in Broiler Chickens. Animals.

[262] Pascual M., Hugas M., Badiola J.I., Monfort J.M., Garriga M. (1999). Lactobacillus salivarius CTC2197 prevents Salmonella enteritidis colonization in chickens. Appl. Environ. Microbiol..

[263] Van Coillie E., Goris J., Cleenwerck I., Grijspeerdt K., Botteldoorn N., Van Immerseel F., De Buck J., Vancanneyt M., Swings J., Herman L. (2007). Identification of lactobacilli isolated from the cloaca and vagina of laying hens and characterization for potential use as probiotics to control Salmonella Enteritidis. J. Appl. Microbiol..

[264] Gil De Los Santos J., Storch O., Gil-Turnes C. (2005). Bacillus cereus var. toyoii and Saccharomyces boulardii increased feed efficiency in broilers infected with Salmonella enteritidis. Br. Poult. Sci..

[265] Okamoto A.S., Andreatti Filho R.L., Milbradt E.L., Moraes A.C.I., Vellano I.H.B., Guimarães-Okamoto P.T.C. (2018). Bacterial communication between Lactobacillus spp. isolated from poultry in the inhibition of Salmonella Heidelberg—Proof of concept. Poult. Sci..

[266] Shao Y., Zhen W., Guo F., Hu Z., Zhang K., Kong L., Guo Y., Wang Z. (2022). Pretreatment with probiotics Enterococcus faecium NCIMB 11181 attenuated Salmonella Typhimurium-induced gut injury through modulating intestinal microbiome and immune responses with barrier function in broiler chickens. J. Anim. Sci. Biotechnol..

[267] Martins F.S., Vieira A.T., Elian S.D., Arantes R.M., Tiago F.C., Sousa L.P., Araújo H.R., Pimenta P.F., Bonjardim C.A., Nicoli J.R. (2013). Inhibition of tissue inflammation and bacterial translocation as one of the protective mechanisms of Saccharomyces boulardii against Salmonella infection in mice. Microbes Infect..

[268] Shanmugasundaram R., Mortada M., Cosby D., Singh M., Applegate T., Syed B., Pender C., Curry S., Murugesan G., Selvaraj R. (2019). Synbiotic supplementation to decrease Salmonella colonization in the intestine and carcass contamination in broiler birds. PLoS ONE.

[269] Kimminau E., Karnezos T., Berghaus R., Jones M., Baxter J., Hofacre C. (2021). Combination of probiotic and prebiotic impacts Salmonella Enteritidis infection in layer hens. J. Appl. Poult. Res..

[270] Medina M., Izquierdo E., Ennahar S., Sanz Y. (2007). Differential immunomodulatory properties of Bifidobacterium logum strains: Relevance to probiotic selection and clinical applications. Clin. Exp. Immunol..

[271] Wan M.L.Y., Forsythe S.J., El-Nezami H. (2019). Probiotics interaction with foodborne pathogens: A potential alternative to antibiotics and future challenges. Crit. Rev. Food Sci. Nutr..

[272] Juricova H., Matiasovicova J., Faldynova M., Sebkova A., Kubasova T., Prikrylova H., Karasova D., Crhanova M., Havlickova H., Rychlik I. (2022). Probiotic Lactobacilli Do Not Protect Chickens against *Salmonella Enteritidis* Infection by Competitive Exclusion in the Intestinal Tract but in Feed, Outside the Chicken Host. Microorganisms.

[273] Khan I., Nawaz M., Anjum A.A., Ahmad M.-u.-D., Mehmood A., Rabbani M., Mustafa A., Ali M.A. (2020). Effect of indigenous probiotics on gut morphology and intestinal absorption capacity in broiler chicken challenged with *Salmonella enteritidis*. Pak. J. Zool..

[274] Steinberg R.S., Silva L.C., Souza T.C., Lima M.T., De Oliveira N.L., Vieira L.Q., Arantes R.M., Miyoshi A., Nicoli J.R., Neumann E. (2014). Safety and protective effectiveness of two strains of Lactobacillus with probiotic features in an experimental model of salmonellosis. Int. J. Environ. Res. Public Health.

[275] Haghighi H.R., Abdul-Careem M.F., Dara R.A., Chambers J.R., Sharif S. (2008). Cytokine gene expression in chicken cecal tonsils following treatment with probiotics and Salmonella infection. Vet. Microbiol..

[276] Casey P.G., Gardiner G.E., Casey G., Bradshaw B., Lawlor P.G., Lynch P.B., Leonard F.C., Stanton C., Ross R.P., Fitzgerald G.F. (2007). A five-strain probiotic combination reduces pathogen shedding and alleviates disease signs in pigs challenged with Salmonella enterica serovar Typhimurium. Appl. Environ. Microbiol..

[277] Truusalu K., Naaber P., Kullisaar T., Tamm H., Mikelsaar R.-H., Zilmer K., Rehema A., Zilmer M., Mikelsaar M. (2004). The influence of antibacterial and antioxidative probiotic lactobacilli on gut mucosa in a mouse model of Salmonella infection. Microb. Ecol. Health Dis..

[278] Chen Q., Tong C., Ma S., Zhou L., Zhao L., Zhao X. (2017). Involvement of microRNAs in probiotics-induced reduction of the cecal inflammation by Salmonella typhimurium. Front. Immunol..

[279] Huang Y.-F., Liu P.-Y., Chen Y.-Y., Nong B.-R., Huang I.-F., Hsieh K.-S., Chen K.-T. (2014). Three-combination probiotics therapy in children with salmonella and rotavirus gastroenteritis. J. Clin. Gastroenterol..

[280] Upadhaya S.D., Shanmugam S.K., Kang D.K., Kim I.H. (2017). Preliminary assessment on potentials of probiotic B. subtilis RX7 and B. methylotrophicus C14 strains as an immune modulator in Salmonella-challenged weaned pigs. Trop. Anim. Health Prod..

[281] Ward M., Alinovi C., Couetil L., Glickman L., Wu C. (2004). randomized clinical trial using probiotics to prevent Salmonella fecal shedding in hospitalized horses. J. Equine Vet. Sci..

[282] Sun C., Gao X., Sun M., Wang Z., Wang Y., Zhao X., Jia F., Zhang T., Ge C., Zhang X. (2022). Protective effects of E. coli Nissle 1917 on chickens infected with Salmonella pullorum. Microb. Pathog..

[283] Smialek M., Kaczorek E., Szczucińska E., Burchardt S., Kowalczyk J., Tykałowski B., Koncicki A. (2019). Evaluation of Lactobacillus spp. and yeast based probiotic (Lavipan) supplementation for the reduction of Salmonella Enteritidis after infection of broiler chickens. Pol. J. Vet. Sci..

[284] Savino F., Fornasero S., Ceratto S., De Marco A., Mandras N., Roana J., Tullio V., Amisano G. (2015). Probiotics and gut health in infants: A preliminary case–control observational study about early treatment with *Lactobacillus reuteri* DSM 17938. Clin. Chim. Acta.

[285] Pineiro M., Asp N.-G., Reid G., Macfarlane S., Morelli L., Brunser O., Tuohy K. (2008). FAO Technical meeting on prebiotics. J. Clin. Gastroenterol..

[286] Cazzola M., Pham-Thi N., Kerihuel J.-C., Durand H., Bohbot S. (2010). Efficacy of a synbiotic supplementation in the prevention of common winter diseases in children: A randomized, double-blind, placebo-controlled pilot study. Ther. Adv. Respir. Dis..

[287] Ojansivu I., Ferreira C.L., Salminen S. (2011). Yacon, a new source of prebiotic oligosaccharides with a history of safe use. Trends Food Sci. Technol..

[288] Passeron T., Lacour J.P., Fontas E., Ortonne J.P. (2006). Prebiotics and synbiotics: Two promising approaches for the treatment of atopic dermatitis in children above 2 years. Allergy.

[289] Macfarlane S., Macfarlane G.T., Cummings J.H. (2006). Review article: Prebiotics in the gastrointestinal tract. Aliment Pharmacol. Ther..

[290] De Vrese M., Schrezenmeir J. (2008). Probiotics, prebiotics, and synbiotics. Food Biotechnol..

[291] Gibson G.R., Probert H.M., Van Loo J., Rastall R.A., Roberfroid M.B. (2004). Dietary modulation of the human colonic microbiota: Updating the concept of prebiotics. Nutr. Res. Rev..

[292] Wang G., Li X., Wang Z. (2016). APD3: The antimicrobial peptide database as a tool for research and education. Nucleic Acids Res..

[293] Helmy Y.A., Closs G., Jung K., Kathayat D., Vlasova A., Rajashekara G. (2022). Effect of Probiotic *E. coli* Nissle 1917 Supplementation on the Growth Performance, Immune Responses, Intestinal Morphology, and Gut Microbes of Campylobacter jejuni Infected Chickens. Infect. Immun..

[294] Helmy Y.A., Kassem I.I., Kumar A., Rajashekara G. (2017). In Vitro Evaluation of the Impact of the Probiotic *E. coli* Nissle 1917 on Campylobacter jejuni’s Invasion and Intracellular Survival in Human Colonic Cells. Front. Microbiol..

[295] Helmy Y.A., Kassem I.I., Rajashekara G. (2021). Immuno-modulatory effect of probiotic E. coli Nissle 1917 in polarized human colonic cells against Campylobacter jejuni infection. Gut Microbes.

[296] Mawad A., Helmy Y.A., Shalkami A.G., Kathayat D., Rajashekara G.E. (2018). coli Nissle microencapsulation in alginate-chitosan nanoparticles and its effect on Campylobacter jejuni in vitro. Appl. Microbiol. Biotechnol..

[297] Al-Sheraji S.H., Ismail A., Manap M.Y., Mustafa S., Yusof R.M., Hassan F.A. (2013). Prebiotics as functional foods: A review. J. Funct. Foods.

[298] Gibson G.R., Scott K.P., Rastall R.A., Tuohy K.M., Hotchkiss A., Dubert-Ferrandon A., Gareau M., Murphy E.F., Saulnier D., Loh G. (2010). Dietary prebiotics: Current status and new definition. Food Sci. Technol. Bull. Funct. Foods.

[299] Chen T. (2003). Effect of adding chicory fructans in feed on fecal and intestinal microflora and excreta volatile ammonia. Int. J. Poult. Sci..

[300] Bailey J., Blankenship L., Cox N. (1991). Effect of fructooligosaccharide on Salmonella colonization of the chicken intestine. Poult. Sci..

[301] Choi K., Namkung H., Paik I. (1994). Effects of dietary fructooligosaccharides on the suppression of intestinal colonization of Salmonella typhimurium in broiler chickens. Korean J. Anim. Sci..

[302] Vicente J., Wolfenden A., Torres-Rodriguez A., Higgins S., Tellez G., Hargis B. (2007). Effect of a Lactobacillus species-based probiotic and dietary lactose prebiotic on turkey poult performance with or without Salmonella enteritidis challenge. J. Appl. Poult. Res..

[303] Sobotik E.B., Ramirez S., Roth N., Tacconi A., Pender C., Murugesan R., Archer G.S. (2021). Evaluating the effects of a dietary synbiotic or synbiotic plus enhanced organic acid on broiler performance and cecal and carcass Salmonella load. Poult. Sci..

[304] Petersen A., Heegaard P.M., Pedersen A.L., Andersen J.B., Sørensen R.B., Frøkiær H., Lahtinen S.J., Ouwehand A.C., Poulsen M., Licht T.R. (2009). Some putative prebiotics increase the severity of *Salmonella enterica* serovar Typhimurium infection in mice. BMC Microbiol..

[305] Ribeiro A.M.L., Vogt L.K., Canal C.W., Cardoso M.d.I., Labres R.V., Streck A.F., Bessa M.C. (2007). Effects of prebiotics and probiotics on the colonization and immune response of broiler chickens challenged with *Salmonella enteritidis*. Braz. J. Poult. Sci..

[306] Rodríguez-Sorrento A., Castillejos L., López-Colom P., Cifuentes-Orjuela G., Rodríguez-Palmero M., Moreno-Muñoz J.A., Martin-Orue S.M. (2020). Effects of Bifidobacterium longum subsp. infantis CECT 7210 and Lactobacillus rhamnosus HN001, combined or not with oligofructose-enriched inulin, on weaned pigs orally challenged with *Salmonella typhimurium*. Front. Microbiol..

[307] Murate L.S., Paião F.G., de Almeida A.M., Berchieri A., Shimokomaki M. (2015). Efficacy of prebiotics, probiotics, and synbiotics on laying hens and broilers challenged with *Salmonella* Enteritidis. J. Poult. Sci..

[308] Huan Y., Kong Q., Mou H., Yi H. (2020). Antimicrobial Peptides: Classification, Design, Application and Research Progress in Multiple Fields. Front. Microbiol..

[309] Lai Y., Villaruz A.E., Li M., Cha D.J., Sturdevant D.E., Otto M. (2007). The human anionic antimicrobial peptide dermcidin induces proteolytic defence mechanisms in staphylococci. Mol. Microbiol..

[310] Malkoski M., Dashper S.G., O’Brien-Simpson N.M., Talbo G.H., Macris M., Cross K.J., Reynolds E.C. (2001). Kappacin, a novel antibacterial peptide from bovine milk. Antimicrob. Agents Chemother..

[311] Oren Z., Shai Y. (1998). Mode of action of linear amphipathic alpha-helical antimicrobial peptides. Biopolymers.

[312] Lohner K., Prossnigg F. (2009). Biological activity and structural aspects of PGLa interaction with membrane mimetic systems. Biochim. Biophys. Acta.

[313] Mardirossian M., Pérébaskine N., Benincasa M., Gambato S., Hofmann S., Huter P., Müller C., Hilpert K., Innis C.A., Tossi A. (2018). The dolphin proline-rich antimicrobial peptide Tur1A inhibits protein synthesis by targeting the bacterial ribosome. Cell Chem. Biol..

[314] Le C.-F., Gudimella R., Razali R., Manikam R., Sekaran S.D. (2016). Transcriptome analysis of Streptococcus pneumoniae treated with the designed antimicrobial peptides, DM3. Sci. Rep..

[315] He S.-w., Zhang J., Li N.-q., Zhou S., Yue B., Zhang M. (2017). A TFPI-1 peptide that induces degradation of bacterial nucleic acids, and inhibits bacterial and viral infection in half-smooth tongue sole, Cynoglossus semilaevis. Fish Shellfish Immunol..

[316] Le C.-F., Fang C.-M., Sekaran S.D. (2017). Intracellular targeting mechanisms by antimicrobial peptides. Antimicrob. Agents Chemother..

[317] Cruz G.F., de Araujo I., Torres M.D., de la Fuente-Nunez C., Oliveira V.X., Ambrosio F.N., Lombello C.B., Almeida D.V., Silva F.D., Garcia W. (2020). Photochemically-generated silver chloride nanoparticles stabilized by a peptide inhibitor of cell division and its antimicrobial properties. J. Inorg. Organomet. Polym. Mater..

[318] Almarwani B., Phambu N., Hamada Y.Z., Sunda-Meya A. (2020). Interactions of an anionic antimicrobial peptide with Zinc (II): Application to bacterial mimetic membranes. Langmuir.

[319] Festa R., Ambrosio R.L., Lamas A., Gratino L., Palmieri G., Franco C.M., Cepeda A., Anastasio A. (2021). A study on the antimicrobial and antibiofilm peptide 1018-K6 as potential alternative to antibiotics against food-pathogen *Salmonella enterica*. Foods.

[320] Sengkhui S., Klubthawee N., Aunpad R. (2023). A novel designed membrane-active peptide for the control of foodborne *Salmonella enterica* serovar *Typhimurium*. Sci. Rep..

[321] Mangmee S., Reamtong O., Kalambaheti T., Roytrakul S., Sonthayanon P. (2021). Antimicrobial Peptide Modifications against Clinically Isolated Antibiotic-Resistant Salmonella. Molecules.

[322] Klubthawee N., Aunpad R. (2021). A Thermostable, Modified Cathelicidin-Derived Peptide With Enhanced Membrane-Active Activity Against *Salmonella enterica* serovar *Typhimurium*. Front. Microbiol..

[323] Forkus B., Ritter S., Vlysidis M., Geldart K., Kaznessis Y.N. (2017). Antimicrobial probiotics reduce *Salmonella enterica* in turkey gastrointestinal tracts. Sci. Rep..

[324] Xu Y., Wang Q., Dong M., Song H., Hang B., Sun Y., Zhang H., Hu J. (2023). Evaluation of the efficacy of the antimicrobial peptide HJH-3 in chickens infected with Salmonella Pullorum. Front. Microbiol..

[325] Yeom J.-H., Lee B., Kim D., Lee J.-k., Kim S., Bae J., Park Y., Lee K. (2016). Gold nanoparticle-DNA aptamer conjugate-assisted delivery of antimicrobial peptide effectively eliminates intracellular *Salmonella enterica* serovar *Typhimurium*. Biomaterials.

[326] Kumaresan V., Bhatt P., Ganesh M.-R., Harikrishnan R., Arasu M., Al-Dhabi N.A., Pasupuleti M., Marimuthu K., Arockiaraj J. (2015). A novel antimicrobial peptide derived from fish goose type lysozyme disrupts the membrane of *Salmonella enterica*. Mol. Immunol..

[327] Tuxpan-Pérez A., Ibarra-Valencia M.A., Estrada B.E., Clement H., Corrales-García L.L., Espino-Solis G.P., Corzo G. (2022). Antimicrobial and Immunomodulatory Effects of Selected Chemokine and Antimicrobial Peptide on Cytokine Profile during *Salmonella Typhimurium* Infection in Mouse. Antibiotics.

[328] Roque-Borda C.A., Pereira L.P., Guastalli E.A.L., Soares N.M., Mac-Lean P.A.B., Salgado D.D.A., Meneguin A.B., Chorilli M., Vicente E.F. (2021). Hpmcp-coated microcapsules containing the ctx (Ile21)-ha antimicrobial peptide reduce the mortality rate caused by resistant *Salmonella enteritidis* in laying hens. Antibiotics.

[329] Bailleul G., Guabiraba R., Virlogeux-Payant I., Lantier I., Trotereau J., Gilbert F.B., Wiedemann A., Trotereau A., Velge P., Schouler C. (2019). Systemic administration of avian defensin 7: Distribution, cellular target, and antibacterial potential in mice. Front. Microbiol..

[330] Maiti S., Patro S., Purohit S., Jain S., Senapati S., Dey N. (2014). Effective control of Salmonella infections by employing combinations of recombinant antimicrobial human β-defensins hBD-1 and hBD-2. Antimicrob. Agents Chemother..

[331] Milona P., Townes C.L., Bevan R.M., Hall J. (2007). The chicken host peptides, gallinacins 4, 7, and 9 have antimicrobial activity against Salmonella serovars. Biochem. Biophys. Res. Commun..

[332] Britannica T. (2020). Argon. Encyclopedia Britannica.

[333] Kasman L.M., Porter L.D. (2021). Bacteriophages. StatPearls.

[334] Doron S., Melamed S., Ofir G., Leavitt A., Lopatina A., Keren M., Amitai G., Sorek R. (2018). Systematic discovery of antiphage defense systems in the microbial pangenome. Science.

[335] Campbell A. (2003). The future of bacteriophage biology. Nat. Rev. Genet..

[336] Wernicki A., Nowaczek A., Urban-Chmiel R. (2017). Bacteriophage therapy to combat bacterial infections in poultry. Virol. J..

[337] Fiorentin L., Vieira N.D., Barioni W. (2005). Oral treatment with bacteriophages reduces the concentration of Salmonella Enteritidis PT4 in caecal contents of broilers. Avian Pathol..

[338] Khan M.A.S., Rahman S.R. (2022). Use of Phages to Treat Antimicrobial-Resistant *Salmonella* Infections in Poultry. Vet. Sci..

[339] Henriques A., Sereno R., Almeida A. (2013). Reducing Salmonella horizontal transmission during egg incubation by phage therapy. Foodborne Pathog. Dis..

[340] Lee S., Kwon T., Chae S.-J., Kim J.-H., Kang Yeon H., Chung Gyung T., Kim D.-W., Lee D.-Y. (2016). Complete Genome Sequence of Bacteriophage MA12, Which Infects both Campylobacter jejuni and Salmonella enterica Serovar Enteritidis. Genome Announc..

[341] Lorenzo-Rebenaque L., Malik D.J., Catalá-Gregori P., Marin C., Sevilla-Navarro S. (2021). In Vitro and In Vivo Gastrointestinal Survival of Non-Encapsulated and Microencapsulated Salmonella Bacteriophages: Implications for Bacteriophage Therapy in Poultry. Pharmaceuticals.

[342] Pelyuntha W., Sanguankiat A., Kovitvadhi A., Vongkamjan K. (2022). Broad lytic spectrum of novel Salmonella phages on ciprofloxacin-resistant Salmonella contaminated in the broiler production chain. Vet. World.

[343] Spricigo D.A., Bardina C., Cortés P., Llagostera M. (2013). Use of a bacteriophage cocktail to control Salmonella in food and the food industry. Int. J. Food Microbiol..

[344] Duc H.M., Son H.M., Honjoh K.-I., Miyamoto T. (2018). Isolation and application of bacteriophages to reduce Salmonella contamination in raw chicken meat. LWT.

[345] Hyman P., Abedon S.T. (2010). Bacteriophage host range and bacterial resistance. Adv. Appl. Microbiol..

[346] Gill J.J., Hyman P. (2010). Phage choice, isolation, and preparation for phage therapy. Curr. Pharm. Biotechnol..

[347] Callaway T.R., Edrington T.S., Brabban A.D., Anderson R.C., Rossman M.L., Engler M.J., Carr M.A., Genovese K.J., Keen J.E., Looper M.L. (2008). Bacteriophage isolated from feedlot cattle can reduce Escherichia coli O157: H7 populations in ruminant gastrointestinal tracts. Foodborne Pathog. Dis..

[348] Tetz G.V., Ruggles K.V., Zhou H., Heguy A., Tsirigos A., Tetz V. (2017). Bacteriophages as potential new mammalian pathogens. Sci. Rep..

[349] Merril C.R. (2008). Interaction of bacteriophages with animals. Bacteriophage Ecology.

[350] Kosznik-Kwaśnicka K., Podlacha M., Grabowski Ł., Stasiłojć M., Nowak-Zaleska A., Ciemińska K., Cyske Z., Dydecka A., Gaffke L., Mantej J. (2022). Biological aspects of phage therapy versus antibiotics against Salmonella enterica serovar Typhimurium infection of chickens. Front. Cell. Infect. Microbiol..

[351] Wong C.L., Sieo C.C., Tan W.S., Abdullah N., Hair-Bejo M., Abu J., Ho Y.W. (2014). Evaluation of a lytic bacteriophage, Φ st1, for biocontrol of Salmonella enterica serovar Typhimurium in chickens. Int. J. Food. Microbiol..

[352] Esmael A., Azab E., Gobouri A.A., Nasr-Eldin M.A., Moustafa M.M.A., Mohamed S.A., Badr O.A.M., Abdelatty A.M. (2021). Isolation and Characterization of Two Lytic Bacteriophages Infecting a Multi-Drug Resistant Salmonella Typhimurium and Their Efficacy to Combat Salmonellosis in Ready-to-Use Foods. Microorganisms.

[353] Bardina C., Spricigo D.A., Cortés P., Llagostera M. (2012). Significance of the bacteriophage treatment schedule in reducing Salmonella colonization of poultry. Appl. Environ. Microbiol..

[354] Atterbury R.J., Van Bergen M., Ortiz F., Lovell M., Harris J., De Boer A., Wagenaar J., Allen V., Barrow P. (2007). Bacteriophage therapy to reduce Salmonella colonization of broiler chickens. Appl. Environ. Microbiol..

[355] Bielke L.R., Higgins S.E., Donoghue A.M., Donoghue D.J., Hargis B.M., Tellez G. (2007). Use of Wide-Host-Range Bacteriophages to Reduce Salmonella on Poultry Products. Int. J. Poult. Sci..

[356] Nabil N.M., Tawakol M.M., Hassan H.M. (2018). Assessing the impact of bacteriophages in the treatment of Salmonella in broiler chickens. Infect. Ecol. Epidemiol..

[357] Lim T.H., Kim M.S., Lee D.H., Lee Y.N., Park J.K., Youn H.N., Lee H.J., Yang S.Y., Cho Y.W., Lee J.B. (2012). Use of bacteriophage for biological control of Salmonella Enteritidis infection in chicken. Res. Vet. Sci..

[358] Sonalika J., Srujana A., Akhila D., Juliet M., Santhosh K. (2020). Application of bacteriophages to control Salmonella Enteritidis in raw eggs. Iran. J. Vet. Res..

[359] Zhang Y., Ding Y., Li W., Zhu W., Wang J., Wang X. (2021). Application of a novel lytic podoviridae phage Pu20 for biological control of drug-resistant Salmonella in liquid eggs. Pathogens.

[360] Rivera D., Moreno-Switt A.I., Denes T.G., Hudson L.K., Peters T.L., Samir R., Aziz R.K., Noben J.-P., Wagemans J., Dueñas F. (2022). Novel Salmonella phage, vB_Sen_STGO-35-1, characterization and evaluation in chicken meat. Microorganisms.

[361] Lipinski C.A., Lombardo F., Dominy B.W., Feeney P.J. (1997). Experimental and computational approaches to estimate solubility and permeability in drug discovery and development settings. Adv. Drug Deliv. Rev..

[362] Maurer C.K., Lu C., Empting M., Hartmann R.W. (2014). Synthetic quorum sensing inhibitors (QSIs) blocking receptor signaling or signal molecule biosynthesis in *Pseudomonas aeruginosa*. Quorum Sensing vs Quorum Quenching: A Battle with No End in Sight.

[363] Tirkkonen H., Brown K.V., Niemczura M., Faudemer Z., Brown C., Ponomareva L.V., Helmy Y.A., Thorson J.S., Nybo S.E., Metsä-Ketelä M. (2023). Engineering BioBricks for Deoxysugar Biosynthesis and Generation of New Tetracenomycins. ACS Omega.

[364] Alshawwa S.Z., Alshallash K.S., Ghareeb A., Elazzazy A.M., Sharaf M., Alharthi A., Abdelgawad F.E., El-Hossary D., Jaremko M., Emwas A.-H. (2022). Assessment of Pharmacological Potential of Novel Exopolysaccharide Isolated from Marine Kocuria sp. Strain AG5: Broad-Spectrum Biological Investigations. Life.

[365] Ta C.A., Arnason J.T. (2015). Mini Review of Phytochemicals and Plant Taxa with Activity as Microbial Biofilm and Quorum Sensing Inhibitors. Molecules.

[366] Escobar-Muciño E., Arenas-Hernández M.M.P., Luna-Guevara M.L. (2022). Mechanisms of Inhibition of Quorum Sensing as an Alternative for the Control of E. coli and Salmonella. Microorganisms.

[367] Guo M., Gamby S., Zheng Y., Sintim H.O. (2013). Small molecule inhibitors of AI-2 signaling in bacteria: State-of-the-art and future perspectives for anti-quorum sensing agents. Int. J. Mol. Sci..

[368] Witsø I.L., Valen Rukke H., Benneche T., Aamdal Scheie A. (2016). Thiophenone attenuates enteropathogenic Escherichia coli O103: H2 virulence by interfering with AI-2 signaling. PLoS ONE.

[369] Vinothkannan R., Muthu Tamizh M., David Raj C., Adline Princy S. (2018). Fructose furoic acid ester: An effective quorum sensing inhibitor against uropathogenic Escherichia coli. Bioorganic Chem..

[370] Li Q., Wang L., Xu J., Liu S., Song Z., Chen T., Deng X., Wang J., Lv Q. (2023). Quercitrin Is a Novel Inhibitor of Salmonella enterica Serovar Typhimurium Type III Secretion System. Molecules.

[371] Deblais L., Helmy Y.A., Kathayat D., Huang H.-C., Miller S.A., Rajashekara G. (2018). Novel Imidazole and Methoxybenzylamine Growth Inhibitors Affecting Salmonella Cell Envelope Integrity and its Persistence in Chickens. Sci. Rep..

[372] Koopman Jacob A., Marshall Joanna M., Bhatiya A., Eguale T., Kwiek Jesse J., Gunn John S. (2014). Inhibition of Salmonella enterica Biofilm Formation Using Small-Molecule Adenosine Mimetics. Antimicrob. Agents Chemother..

[373] Nagy T.A., Quintana J.L.J., Reens A.L., Crooks A.L., Detweiler C.S. (2019). Autophagy Induction by a Small Molecule Inhibits Salmonella Survival in Macrophages and Mice. Antimicrob. Agents Chemother..

[374] Reens A.L., Crooks A.L., Su C.-C., Nagy T.A., Reens D.L., Podoll J.D., Edwards M.E., Yu E.W., Detweiler C.S. (2018). A cell-based infection assay identifies efflux pump modulators that reduce bacterial intracellular load. PLoS Pathog..

[375] Bakowski M.A., Braun V., Brumell J.H. (2008). Salmonella-containing vacuoles: Directing traffic and nesting to grow. Traffic.

[376] Stevens A.M., Schuster M., Rumbaugh K.P. (2012). Working together for the common good: Cell-cell communication in bacteria. J. Bacteriol..

[377] Helmy Yosra A., Kathayat D., Deblais L., Srivastava V., Closs G., Tokarski Robert J., Ayinde O., Fuchs James R., Rajashekara G. (2022). Evaluation of Novel Quorum Sensing Inhibitors Targeting Auto-Inducer 2 (AI-2) for the Control of Avian Pathogenic Escherichia coli Infections in Chickens. Microbiol. Spectr..

[378] Linciano P., Cavalloro V., Martino E., Kirchmair J., Listro R., Rossi D., Collina S. (2020). Tackling antimicrobial resistance with small molecules targeting LsrK: Challenges and opportunities. J. Med. Chem..

[379] Helmy Y.A., Deblais L., Kassem I.I., Kathayat D., Rajashekara G. (2018). Novel small molecule modulators of quorum sensing in avian pathogenic Escherichia coli (APEC). Virulence.

[380] Winzer K., Hardie K.R., Williams P. (2003). LuxS and autoinducer-2: Their contribution to quorum sensing and metabolism in bacteria. Adv. Appl. Microbiol..

[381] Sholpan A., Lamas A., Cepeda A., Franco C.M. (2021). Salmonella spp. quorum sensing: An overview from environmental persistence to host cell invasion. AIMS Microbiol..

[382] Smith D., Wang J.-H., Swatton J.E., Davenport P., Price B., Mikkelsen H., Stickland H., Nishikawa K., Gardiol N., Spring D.R. (2006). Variations on a theme: Diverse N-acyl homoserine lactone-mediated quorum sensing mechanisms in gram-negative bacteria. Sci. Prog..

[383] Johnson Jeremiah G., Yuhas C., McQuade Thomas J., Larsen Martha J., DiRita Victor J. (2015). Narrow-Spectrum Inhibitors of Campylobacter jejuni Flagellar Expression and Growth. Antimicrob. Agents Chemother..

[384] Choi J., Shin D., Ryu S. (2007). Implication of quorum sensing in Salmonella enterica serovar Typhimurium virulence: The luxS gene is necessary for expression of genes in pathogenicity island 1. Infect. Immun..

[385] Deblais L., Helmy Y.A., Kumar A., Antwi J., Kathayat D., Acuna U.M., Huang H.-c., de Blanco E.C., Fuchs J.R., Rajashekara G. (2019). Novel narrow spectrum benzyl thiophene sulfonamide derivatives to control Campylobacter. J. Antibiot..

[386] Johnson T.J., Shank J.M., Johnson J.G. (2017). Current and Potential Treatments for Reducing Campylobacter Colonization in Animal Hosts and Disease in Humans. Front. Microbiol..

[387] Helmy Y.A., Kathayat D., Closs G., Galgozy K., Fuchs J.R., Rajashekara G. (2023). Efficacy of quorum sensing and growth inhibitors alone and in combination against avian pathogenic Escherichia coli infection in chickens. Poult. Sci..

[388] Li G., Yan C., Xu Y., Feng Y., Wu Q., Lv X., Yang B., Wang X., Xia X. (2014). Punicalagin inhibits Salmonella virulence factors and has anti-quorum-sensing potential. Appl. Environ. Microbiol..

[389] Upadhyaya I., Upadhyay A., Kollanoor-Johny A., Darre M.J., Venkitanarayanan K. (2013). Effect of plant derived antimicrobials on Salmonella enteritidis adhesion to and invasion of primary chicken oviduct epithelial cells in vitro and virulence gene expression. Int. J. Mol. Sci..

[390] Janssens J.C., Steenackers H., Robijns S., Gellens E., Levin J., Zhao H., Hermans K., De Coster D., Verhoeven T.L., Marchal K. (2008). Brominated furanones inhibit biofilm formation by Salmonella enterica serovar Typhimurium. Appl. Environ. Microbiol..

[391] Birhanu B.T., Park N.-H., Lee S.-J., Hossain M.A., Park S.-C. (2018). Inhibition of Salmonella Typhimurium adhesion, invasion, and intracellular survival via treatment with methyl gallate alone and in combination with marbofloxacin. Vet. Res..

[392] Styles M.J., Blackwell H.E. (2018). Non-native autoinducer analogs capable of modulating the SdiA quorum sensing receptor in Salmonella enterica serovar Typhimurium. Beilstein J. Org. Chem..

[393] Nesterenko L.N., Zigangirova N.A., Zayakin E.S., Luyksaar S.I., Kobets N.V., Balunets D.V., Shabalina L.A., Bolshakova T.N., Dobrynina O.Y., Gintsburg A.L. (2016). A small-molecule compound belonging to a class of 2,4-disubstituted 1,3,4-thiadiazine-5-ones suppresses Salmonella infection in vivo. J. Antibiot..

[394] Song Y., Xu G., Li C., Li Z., Lu C., Shen Y. (2021). Structural optimization of natural product fusaric acid to discover novel T3SS inhibitors of Salmonella. Biochem. Biophys. Res. Commun..

[395] Hudson Debra L., Layton Abigail N., Field Terry R., Bowen Alison J., Wolf-Watz H., Elofsson M., Stevens Mark P., Galyov Edouard E. (2007). Inhibition of Type III Secretion in *Salmonella enterica* Serovar *Typhimurium* by Small-Molecule Inhibitors. Antimicrob. Agents Chemother..

[396] Defoirdt T. (2018). Quorum-sensing systems as targets for antivirulence therapy. Trends Microbiol..

[397] Krzyżek P. (2019). Challenges and Limitations of Anti-quorum Sensing Therapies. Front. Microbiol..

[398] Mechesso A.F., Yixian Q., Park S.C. (2019). Methyl gallate and tylosin synergistically reduce the membrane integrity and intracellular survival of Salmonella Typhimurium. PLoS ONE.

[399] Aswathanarayan J.B., Vittal R.R. (2018). Inhibition of biofilm formation and quorum sensing mediated phenotypes by berberine in Pseudomonas aeruginosa and Salmonella typhimurium. RSC Adv..

[400] Sivasankar C., Jha N.K., Ghosh R., Shetty P.H. (2020). Anti quorum sensing and anti virulence activity of tannic acid and it’s potential to breach resistance in Salmonella enterica Typhi/Paratyphi A clinical isolates. Microb. Pathog..

[401] Durães F., Resende D., Palmeira A., Szemerédi N., Pinto M.M.M., Spengler G., Sousa E. (2021). Xanthones Active against Multidrug Resistance and Virulence Mechanisms of Bacteria. Antibiotics.

[402] Janssens Joost C.A., Metzger K., Daniels R., Ptacek D., Verhoeven T., Habel Lothar W., Vanderleyden J., De Vos Dirk E., De Keersmaecker Sigrid C.J. (2007). Synthesis of N-Acyl Homoserine Lactone Analogues Reveals Strong Activators of SdiA, the Salmonella enterica Serovar Typhimurium LuxR Homologue. Appl. Environ. Microbiol..

[403] Tsai C.N., MacNair C.R., Cao M.P.T., Perry J.N., Magolan J., Brown E.D., Coombes B.K. (2020). Targeting Two-Component Systems Uncovers a Small-Molecule Inhibitor of *Salmonella* Virulence. Cell Chem. Biol..

[404] Li J., Lv C., Sun W., Li Z., Han X., Li Y., Shen Y. (2013). Cytosporone B, an Inhibitor of the Type III Secretion System of Salmonella enterica Serovar Typhimurium. Antimicrob. Agents Chemother..

[405] CDC Immunization: The Basics. https://www.cdc.gov/vaccines/vac-gen/imz-basics.htm.

[406] Plotkin S.A. (2020). Updates on immunologic correlates of vaccine-induced protection. Vaccine.

[407] Allen H.K., Levine U.Y., Looft T., Bandrick M., Casey T.A. (2013). Treatment, promotion, commotion: Antibiotic alternatives in food-producing animals. Trends Microbiol..

[408] Elaish M., Ngunjiri J.M., Ali A., Xia M., Ibrahim M., Jang H., Hiremath J., Dhakal S., Helmy Y.A., Jiang X. (2017). Supplementation of inactivated influenza vaccine with norovirus P particle-M2e chimeric vaccine enhances protection against heterologous virus challenge in chickens. PLoS ONE.

[409] Fawzy M., Helmy Y.A. (2019). The One Health Approach is Necessary for the Control of Rift Valley Fever Infections in Egypt: A Comprehensive Review. Viruses.

[410] Helmy Y.A., Fawzy M., Elaswad A., Sobieh A., Kenney S.P., Shehata A.A. (2020). The COVID-19 Pandemic: A Comprehensive Review of Taxonomy, Genetics, Epidemiology, Diagnosis, Treatment, and Control. J. Clin. Med..

[411] Taha-Abdelaziz K., Singh M., Sharif S., Sharma S., Kulkarni R.R., Alizadeh M., Yitbarek A., Helmy Y.A. (2023). Intervention Strategies to Control Campylobacter at Different Stages of the Food Chain. Microorganisms.

[412] Lucero M.S., Chimeno Zoth S., Jaton J., Gravisaco M.J., Pinto S., Richetta M., Berinstein A., Gómez E. (2021). Oral Immunization With Plant-Based Vaccine Induces a Protective Response Against Infectious Bursal Disease. Front. Plant Sci..

[413] Yadav D.K., Yadav N., Khurana S.M.P. (2020). Vaccines: Present status and applications. Animal Biotechnology.

[414] Tumpey T.M., Alvarez R., Swayne D.E., Suarez D.L. (2005). Diagnostic approach for differentiating infected from vaccinated poultry on the basis of antibodies to NS1, the nonstructural protein of influenza A virus. J. Clin. Microbiol..

[415] Francis M.J. (2018). Recent Advances in Vaccine Technologies. Vet. Clin. N. Am. Small Anim. Pract..

[416] Vanderslott S., Dattani S., Spooner F., Roser M. (2013). Vaccination.

[417] Parham P. (2021). The Immune System: Fifth International Student Edition with Registration Card.

[418] Lidder P., Sonnino A. (2012). Biotechnologies for the management of genetic resources for food and agriculture. Adv. Genet..

[419] Micoli F., MacLennan C.A. (2020). Outer membrane vesicle vaccines. Proceedings of the Seminars in Immunology.

[420] Rappuoli R. (2018). Glycoconjugate vaccines: Principles and mechanisms. Sci. Transl. Med..

[421] Clarke E.T., Williams N.A., Dull P.M., Findlow J., Borrow R., Finn A., Heyderman R.S. (2013). Polysaccharide–protein conjugate vaccination induces antibody production but not sustained B-cell memory in the human nasopharyngeal mucosa. Mucosal Immunol..

[422] Ura T., Okuda K., Shimada M. (2014). Developments in viral vector-based vaccines. Vaccines.

[423] Gheibi Hayat S.M., Darroudi M. (2019). Nanovaccine: A novel approach in immunization. J. Cell. Physiol..

[424] MacLennan C.A., Martin L.B., Micoli F. (2014). Vaccines against invasive Salmonella disease: Current status and future directions. Hum. Vaccines Immunother..

[425] Germanier R., Fiirer E. (1975). Isolation and characterization of Gal E mutant Ty 21a of *Salmonella typhi*: A candidate strain for a live, oral typhoid vaccine. J. Infect. Dis..

[426] Levine M.M., Ferreccio C., Black R.E., Lagos R., Martin O.S., Blackwelder W.C. (2007). Ty21a live oral typhoid vaccine and prevention of paratyphoid fever caused by *Salmonella enterica* Serovar *Paratyphi* B. Clin. Infect. Dis..

[427] Tagliabue A., Villa L., De Magistris M.T., Romano M., Silvestri S., Boraschi D., Nencioni L. (1986). IgA-driven T cell-mediated anti-bacterial immunity in man after live oral Ty 21a vaccine. J. Immunol..

[428] Lindow J.C., Fimlaid K.A., Bunn J.Y., Kirkpatrick B.D. (2011). Antibodies in action: Role of human opsonins in killing *Salmonella enterica* serovar Typhi. Infect. Immun..

[429] Wahid R., Simon R., Zafar S.J., Levine M.M., Sztein M.B. (2012). Live oral typhoid vaccine Ty21a induces cross-reactive humoral immune responses against Salmonella enterica serovar Paratyphi A and S. Paratyphi B in humans. Clin. Vaccine Immunol..

[430] Chinnasami B., Sadasivam K., Vivekanandhan A., Arunachalam P., Pasupathy S. (2015). A study on longevity of immune response after vaccination with Salmonella Typhi Vi conjugate vaccine (Pedatyph™) in children. J. Clin. Diagn. Res..

[431] Crump J.A., Oo W.T. (2021). Salmonella Typhi Vi polysaccharide conjugate vaccine protects infants and children against typhoid fever. Lancet.

[432] Jossi S.E., Arcuri M., Alshayea A., Persaud R.R., Marcial-Juárez E., Palmieri E., Di Benedetto R., Pérez-Toledo M., Pillaye J., Channell W.M. (2023). Vi polysaccharide and conjugated vaccines afford similar early, IgM or IgG-independent control of infection but boosting with conjugated Vi vaccines sustains the efficacy of immune responses. Front. Immunol..

[433] Micoli F., Rondini S., Pisoni I., Giannelli C., Di Cioccio V., Costantino P., Saul A., Martin L. (2012). Production of a conjugate vaccine for Salmonella enterica serovar Typhi from Citrobacter Vi. Vaccine.

[434] Lyon C.E., Sadigh K.S., Carmolli M.P., Harro C., Sheldon E., Lindow J.C., Larsson C.J., Martinez T., Feller A., Ventrone C.H. (2010). In a randomized, double-blinded, placebo-controlled trial, the single oral dose typhoid vaccine, M01ZH09, is safe and immunogenic at doses up to 1.7×1010 colony-forming units. Vaccine.

[435] Smith G.W., Smith F., Zuidhof S., Foster D.M. (2015). Short communication: Characterization of the serologic response induced by vaccination of late-gestation cows with a Salmonella Dublin vaccine. J. Dairy Sci..

[436] Renu S., Han Y., Dhakal S., Lakshmanappa Y.S., Ghimire S., Feliciano-Ruiz N., Senapati S., Narasimhan B., Selvaraj R., Renukaradhya G.J. (2020). Chitosan-adjuvanted Salmonella subunit nanoparticle vaccine for poultry delivered through drinking water and feed. Carbohydr. Polym..

[437] Muniz E.C., Verdi R., Leão J.A., Back A., Nascimento V.P.d. (2017). Evaluation of the effectiveness and safety of a genetically modified live vaccine in broilers challenged with Salmonella Heidelberg. Avian Pathol..

[438] Crouch C.F., Nell T., Reijnders M., Donkers T., Pugh C., Patel A., Davis P., van Hulten M.C.W., de Vries S.P.W. (2020). Safety and efficacy of a novel inactivated trivalent Salmonella enterica vaccine in chickens. Vaccine.

[439] Pollard A.J., Bijker E.M. (2021). A guide to vaccinology: From basic principles to new developments. Nat. Rev. Immunol..

[440] Moyle P.M., Toth I. (2013). Modern subunit vaccines: Development, components, and research opportunities. ChemMedChem.

[441] Tekle Y.I., Nielsen K.M., Liu J., Pettigrew M.M., Meyers L.A., Galvani A.P., Townsend J.P. (2012). Controlling antimicrobial resistance through targeted, vaccine-induced replacement of strains. PLoS ONE.

[442] Huberman Y.D., Velilla A.V., Terzolo H.R. (2019). Evaluation of different live Salmonella enteritidis vaccine schedules administered during layer hen rearing to reduce excretion, organ colonization, and egg contamination. Poult. Sci..

[443] Baranyi J., Roberts T.A. (1994). A dynamic approach to predicting bacterial growth in food. Int. J. Food Microbiol..

[444] Theron M.M., Lues J.F.R. (2007). Organic Acids and Meat Preservation: A Review. Food Rev. Int..

[445] Taylor M., Joerger R., Palou E., López-Malo A., Avila-Sosa R., Calix-Lara T. (2012). Alternatives to traditional antimicrobials for organically processed meat and poultry. Org. Meat Prod. Process..

[446] Yoon B.K., Jackman J.A., Valle-González E.R., Cho N.-J. (2018). Antibacterial free fatty acids and monoglycerides: Biological activities, experimental testing, and therapeutic applications. Int. J. Mol. Sci..

[447] Gómez-García M., Sol C., de Nova P.J.G., Puyalto M., Mesas L., Puente H., Mencía-Ares Ó., Miranda R., Argüello H., Rubio P. (2019). Antimicrobial activity of a selection of organic acids, their salts and essential oils against swine enteropathogenic bacteria. Porc. Health Manag..

[448] Ng W.-K., Koh C.-B. (2017). The utilization and mode of action of organic acids in the feeds of cultured aquatic animals. Rev. Aquac..

[449] Ricke S.C. (2003). Perspectives on the use of organic acids and short chain fatty acids as antimicrobials. Poult. Sci..

[450] Dittoe D.K., Ricke S.C., Kiess A.S. (2018). Organic Acids and Potential for Modifying the Avian Gastrointestinal Tract and Reducing Pathogens and Disease. Front. Vet. Sci..

[451] Ricke S.C., Dittoe D.K., Richardson K.E. (2020). Formic Acid as an Antimicrobial for Poultry Production: A Review. Front. Vet. Sci..

[452] Desbois A.P., Smith V.J. (2010). Antibacterial free fatty acids: Activities, mechanisms of action and biotechnological potential. Appl. Microbiol. Biotechnol..

[453] Hensel M. (2004). Evolution of pathogenicity islands of Salmonella enterica. Int. J. Med. Microbiol..

[454] Sprong R.C., Hulstein M.F., Van der Meer R. (2001). Bactericidal activities of milk lipids. Antimicrob. Agents Chemother..

[455] Immerseel F.V., Buck J.D., Smet I.D., Pasmans F., Haesebrouck F., Ducatelle R. (2004). Interactions of butyric acid–and acetic acid–treated Salmonella with chicken primary Cecal epithelial cells in vitro. Avian Dis..

[456] Durant J.A., Corrier D.E., Ricke S.C. (2000). Short-chain volatile fatty acids modulate the expression of the hilA and invF genes of Salmonella typhimurium. J. Food Prot..

[457] Koyuncu S., Andersson M.G., Löfström C., Skandamis P.N., Gounadaki A., Zentek J., Häggblom P. (2013). Organic acids for control of Salmonella in different feed materials. BMC Vet. Res..

[458] Menconi A., Reginatto A., Londero A., Pumford N., Morgan M., Hargis B., Tellez G. (2013). Effect of organic acids on Salmonella Typhimurium infection in broiler chickens. Int. J. Poult. Sci..

[459] Ruhnke I., Röhe I., Goodarzi Boroojeni F., Knorr F., Mader A., Hafeez A., Zentek J. (2015). Feed supplemented with organic acids does not affect starch digestibility, nor intestinal absorptive or secretory function in broiler chickens. J. Anim. Physiol. Anim. Nutr..

[460] Lynch H., Leonard F.C., Walia K., Lawlor P.G., Duffy G., Fanning S., Markey B.K., Brady C., Gardiner G.E., Argüello H. (2017). Investigation of in-feed organic acids as a low cost strategy to combat Salmonella in grower pigs. Prev. Vet. Med..

[461] Tsai C.-C., Hsih H.-Y., Chiu H.-H., Lai Y.-Y., Liu J.-H., Yu B., Tsen H.-Y. (2005). Antagonistic activity against Salmonella infection in vitro and in vivo for two Lactobacillus strains from swine and poultry. Int. J. Food Microbiol..

[462] Gunal M., Yayli G., Kaya O., Karahan N., Sulak O. (2006). The effects of antibiotic growth promoter, probiotic or organic acid supplementation on performance, intestinal microflora and tissue of broilers. Int. J. Poult. Sci..

[463] Warnecke T., Gill R.T. (2005). Organic acid toxicity, tolerance, and production in Escherichia coli biorefining applications. Microb. Cell Factories.

[464] Khan S.H., Iqbal J. (2016). Recent advances in the role of organic acids in poultry nutrition. J. Appl. Anim. Res..

[465] Brah A.S., Armah F.A., Obuah C., Akwetey S.A., Adokoh C.K. (2023). Toxicity and therapeutic applications of citrus essential oils (CEOs): A review. Int. J. Food Prop..

[466] Bakkali F., Averbeck S., Averbeck D., Idaomar M. (2008). Biological effects of essential oils—A review. Food Chem. Toxicol..

[467] Angioni A., Barra A., Coroneo V., Dessi S., Cabras P. (2006). Chemical composition, seasonal variability, and antifungal activity of Lavandula stoechas L. ssp. stoechas essential oils from stem/leaves and flowers. J. Agric. Food Chem..

[468] Inouye S., Abe S., Yamaguchi H., Asakura M. (2003). Comparative study of antimicrobial and cytotoxic effects of selected essential oils by gaseous and solution contacts. Int. J. Aromather..

[469] Costa D.C., Costa H., Albuquerque T.G., Ramos F., Castilho M.C., Sanches-Silva A. (2015). Advances in phenolic compounds analysis of aromatic plants and their potential applications. Trends Food Sci. Technol..

[470] Lubbe A., Verpoorte R. (2011). Cultivation of medicinal and aromatic plants for specialty industrial materials. Ind. Crops. Prod..

[471] Bansal T. (2016). Benefits of essential oil. J. Chem. Pharm. Res..

[472] O’Brien T.F. (2002). Emergence, spread, and environmental effect of antimicrobial resistance: How use of an antimicrobial anywhere can increase resistance to any antimicrobial anywhere else. Clin. Infect. Dis..

[473] Swamy M.K., Akhtar M.S., Sinniah U.R. (2016). Antimicrobial Properties of Plant Essential Oils against Human Pathogens and Their Mode of Action: An Updated Review. Evid.-Based Complement. Altern. Med..

[474] Park J.B., Kang J.H., Song K.B. (2018). Antibacterial activities of a cinnamon essential oil with cetylpyridinium chloride emulsion against Escherichia coli O157:H7 and Salmonella Typhimurium in basil leaves. Food Sci. Biotechnol..

[475] Raybaudi-Massilia R.M., Mosqueda-Melgar J., Martin-Belloso O. (2006). Antimicrobial Activity of Essential Oils on Salmonella Enteritidis, Escherichia coli, and Listeria innocua in Fruit Juices. J. Food Prot..

[476] Ebani V.V., Nardoni S., Bertelloni F., Tosi G., Massi P., Pistelli L., Mancianti F. (2019). In Vitro Antimicrobial Activity of Essential Oils Against *Salmonella enterica* Serotypes Enteritidis and Typhimurium Strains Isolated from Poultry. Molecules.

[477] Olaimat A.N., Al-Holy M.A., Abu Ghoush M.H., Al-Nabulsi A.A., Osaili T.M., Holley R.A. (2019). Inhibitory effects of cinnamon and thyme essential oils against *Salmonella* spp. in hummus (chickpea dip). J. Food Process. Preserv..

[478] Thanissery R., Kathariou S., Smith D.P. (2014). Rosemary oil, clove oil, and a mix of thyme-orange essential oils inhibit Salmonella and Campylobacter in vitro. J. Appl. Poult. Res..

[479] Puškárová A., Bučková M., Kraková L., Pangallo D., Kozics K. (2017). The antibacterial and antifungal activity of six essential oils and their cyto/genotoxicity to human HEL 12469 cells. Sci. Rep..

[480] Slameňová D., Horváthová E., Kováčiková Z., Kozics K., Hunáková Ľ. (2011). Essential rosemary oil protects testicular cells against DNA-damaging effects of H_2_O_2_ and DMNQ. Food Chem..

[481] Maurya A., Prasad J., Das S., Dwivedy A.K. (2021). Essential Oils and Their Application in Food Safety. Front. Sustain. Food Syst..

[482] Millet Y., Jouglard J., Steinmetz M., Tognetti P., Joanny P., Arditti J. (1981). Toxicity of some essential plant oils. Clinical and experimental study. Clin. Toxicol..

[483] Posadzki P., Alotaibi A., Ernst E. (2012). Adverse effects of aromatherapy: A systematic review of case reports and case series. Int. J. Risk Saf. Med..

[484] Fadil M., Fikri-Benbrahim K., Rachiq S., Ihssane B., Lebrazi S., Chraibi M., Haloui T., Farah A. (2018). Combined treatment of *Thymus vulgaris* L., *Rosmarinus officinalis* L. and *Myrtus communis* L. essential oils against *Salmonella typhimurium*: Optimization of antibacterial activity by mixture design methodology. Eur. J. Pharm. Biopharm..

[485] Barbosa L.N., Alves F.C.B., Andrade B.F.M.T., Albano M., Rall V.L.M., Fernandes A.A.H., Buzalaf M.A.R., Leite A.d.L., de Pontes L.G., dos Santos L.D. (2020). Proteomic analysis and antibacterial resistance mechanisms of *Salmonella Enteritidis* submitted to the inhibitory effect of Origanum vulgare essential oil, thymol and carvacrol. J. Proteom..

[486] Hasheminya S.-M., Dehghannya J. (2020). Composition, phenolic content, antioxidant and antimicrobial activity of *Pistacia atlantica* subsp. kurdica hulls’ essential oil. Food Biosci..

[487] Alibi S., Selma W.B., Ramos-Vivas J., Smach M.A., Touati R., Boukadida J., Navas J., Mansour H.B. (2020). Anti-oxidant, antibacterial, anti-biofilm, and anti-quorum sensing activities of four essential oils against multidrug-resistant bacterial clinical isolates. Curr. Res. Transl. Med..

[488] Rattanachaikunsopon P., Phumkhachorn P. (2010). Antimicrobial Activity of Basil (*Ocimum basilicum*) Oil against Salmonella Enteritidis in Vitro and in Food. Biosci. Biotechnol. Biochem..

[489] Morshdy A., El-Tahlawy A.S., Qari S.H., Qumsani A.T., Bay D.H., Sami R., Althubaiti E.H., Mansour A.M.A., Aljahani A.H., Hafez A.E.E. (2022). Anti-Biofilms’ Activity of Garlic and Thyme Essential Oils against *Salmonella typhimurium*. Molecules.

[490] Hu Z., Liu L., Guo F., Huang J., Qiao J., Bi R., Huang J., Zhang K., Guo Y., Wang Z. (2023). Dietary supplemental coated essential oils and organic acids mixture improves growth performance and gut health along with reduces *Salmonella* load of broiler chickens infected with *Salmonella* Enteritidis. J. Anim. Sci. Biotechnol..

[491] Moore-Neibel K., Gerber C., Patel J., Friedman M., Ravishankar S. (2012). Antimicrobial activity of lemongrass oil against Salmonella enterica on organic leafy greens. J. Appl. Microbiol..

